# Analysis of epigenetic features characteristic of L1 loci expressed in human cells

**DOI:** 10.1093/nar/gkac013

**Published:** 2022-01-31

**Authors:** Benjamin Freeman, Travis White, Tiffany Kaul, Emily C Stow, Melody Baddoo, Nathan Ungerleider, Maria Morales, Hanlin Yang, Dawn Deharo, Prescott Deininger, Victoria P Belancio

**Affiliations:** Department of Structural and Cellular Biology, Tulane University School of Medicine, Tulane Cancer Center, Tulane Center for Aging, New Orleans, LA 70112, USA; Tulane Cancer Center, Tulane Health Sciences Center, 1700 Tulane Ave, New Orleans, LA 70112, USA; Sloan Kettering Institute for Cancer Research, NY, NY 10065, USA; Tulane Cancer Center, Tulane Health Sciences Center, 1700 Tulane Ave, New Orleans, LA 70112, USA; Department of Structural and Cellular Biology, Tulane University School of Medicine, Tulane Cancer Center, Tulane Center for Aging, New Orleans, LA 70112, USA; Tulane Cancer Center, Tulane Health Sciences Center, 1700 Tulane Ave, New Orleans, LA 70112, USA; Department of Pathology, Tulane University School of Medicine, Tulane Cancer Center, New Orleans, LA 70112, USA; Department of Pathology, Tulane University School of Medicine, Tulane Cancer Center, New Orleans, LA 70112, USA; Tulane Cancer Center, Tulane Health Sciences Center, 1700 Tulane Ave, New Orleans, LA 70112, USA; Tulane Cancer Center, Tulane Health Sciences Center, 1700 Tulane Ave, New Orleans, LA 70112, USA; Department of Structural and Cellular Biology, Tulane University School of Medicine, Tulane Cancer Center, Tulane Center for Aging, New Orleans, LA 70112, USA; Tulane Cancer Center, Tulane Health Sciences Center, 1700 Tulane Ave, New Orleans, LA 70112, USA; Tulane Cancer Center, Tulane Health Sciences Center, 1700 Tulane Ave, New Orleans, LA 70112, USA; Department of Epidemiology, Tulane School of Public Health and Tropical Medicine, New Orleans, LA 70112, USA; Department of Structural and Cellular Biology, Tulane University School of Medicine, Tulane Cancer Center, Tulane Center for Aging, New Orleans, LA 70112, USA; Tulane Cancer Center, Tulane Health Sciences Center, 1700 Tulane Ave, New Orleans, LA 70112, USA

## Abstract

Only a select few L1 loci in the human genome are expressed in any given cell line or organ, likely to minimize damage done to the genome. The epigenetic features and requirements of expressed L1 loci are currently unknown. Using human cells and comprehensive epigenetic analysis of individual expressed and unexpressed L1 loci, we determined that endogenous L1 transcription depends on a combination of epigenetic factors, including open chromatin, activating histone modifications, and hypomethylation at the L1 promoter. We demonstrate that the L1 promoter seems to require interaction with enhancer elements for optimal function. We utilize epigenetic context to predict the expression status of L1Hs loci that are poorly mappable with RNA-Seq. Our analysis identified a population of ‘transitional’ L1 loci that likely have greater potential to be activated during the epigenetic dysregulation seen in tumors and during aging because they are the most responsive to targeted CRISPR-mediated delivery of trans-activating domains. We demonstrate that an engineered increase in endogenous L1 mRNA expression increases Alu mobilization. Overall, our findings present the first global and comprehensive analysis of epigenetic status of individual L1 loci based on their expression status and demonstrate the importance of epigenetic context for L1 expression heterogeneity.

## INTRODUCTION

Retrotransposons are mobile elements that have colonized the human genome for tens of millions of years (1). These elements increase their copy number in the host genome via a copy-and-paste mechanism involving an RNA intermediate. As a result, retrotransposon sequences make up almost half of the human genome (2). Long Interspersed Element-1 (L1) is the only active, autonomous retrotransposon in humans, accounting for ∼17% of the genome (2,3). These elements consist of a 5′ UTR containing a promoter sequence, two open reading frames (termed ORF1 and ORF2), and a polyA tail defining the end of their 3′ UTR (4,5). ORF1 and ORF2 encode two proteins, ORF1p and ORF2p, that are necessary for L1 retrotransposition (6–9).

In order to create a new copy, a full-length L1 element is first transcribed into L1 mRNA which is then exported to the cytoplasm (5). The L1 mRNA is translated to produce ORF1p and ORF2p, which associate with their parental mRNA in order to form the L1 ribonucleoprotein (RNP) (10–14). ORF1p trimerizes and coats the L1 mRNA, which is likely important for stabilization of the L1 RNP and has a putative role in L1 RNP nuclear import (6,7,15). ORF2p is generated at much lower levels than ORF1p (16,17). It contains endonuclease and reverse transcriptase activities (8,9) needed for the genomic insertion process. After formation in the cytoplasm, the L1 RNP localizes to the nucleus and accesses the chromatin to generate a new copy through target-primed reverse transcription (TPRT), the process in which the L1 mRNA is reverse-transcribed into DNA by ORF2p and integrated into the chromatin (18–20). This copy-and-paste mode of amplification has resulted in the accumulation of over 500,000 L1 insertions in the human genome. Approximately 5,000 of these are full-length L1 elements capable of expression from an L1 promoter. The remaining L1 elements are truncated at the 5′ end during insertion (21,22). Without the 5′ UTR containing the L1 internal promoter sequence, these truncated L1 elements are not considered active for retrotransposition as they cannot form the L1 mRNA.

Multiple mechanisms contribute to the suppression of L1 retrotransposition (23). The first step of suppression occurs at the transcriptional level, as epigenetic silencing and/or accumulation of mutations in the L1 internal promoter can suppress expression of full-length elements (21,22). The existence of deliberate epigenetic silencing of L1 expression by the host is supported by reactivation of L1 expression associated with engineered or natural loss of functions involved in epigenetic maintenance (24,25) and in pathological states associated with epigenetic remodeling such as cancers and induced cell reprogramming (23,26–29). Furthermore, recent efforts to characterize L1 mRNA expression at single locus resolution have determined that, in any given cell line, most L1 transcripts come from a few loci, including from loci of the L1Hs sub-family, the youngest and most active L1 sub-family (21,22,30–32). Studies of endogenous L1 mRNA expression in mouse organs determined that this finding is true *in vivo* because only different small sets of L1 loci are expressed in each organ (33). These data strongly support the potential that genomic environment influences which L1 loci can be expressed.

A number of epigenetic factors have been studied in connection with bulk L1 expression (23,34–36). The biggest weakness of these studies is that they analyze epigenetic marks at promoters of all L1 loci or those belonging to a specific sub-family. This was the result of previous technical limitations preventing analysis of L1 mRNA expression from individual loci.

Several typical mechanisms of transcriptional silencing have been identified in mammalian cells. Most thoroughly studied thus far are the effects of promoter methylation on repetitive elements (36–49). Methylation of CpG islands leads to stable transcriptional silencing by recruiting proteins involved in transcriptional repression and physically blocking transcription factor binding (50,51). CpG islands are overrepresented in promoter regions of the human genome (42) and are a key feature of the L1 promoter (38,39). Studies on L1 methylation highlight the importance of methylation in restricting LINE-1 elements, especially younger and more active sub-families (31). CpG demethylation has been shown to enhance L1 expression (31,36), but hypomethylation is not an absolute requirement for L1 expression (44). Recent studies that characterized promoter methylation of specific expressed loci have focused on only a handful of L1 elements. Scott *et al.* identified a hyperactive L1Hs element in somatic cells that contains a CpG mutation likely resulting in its hypomethylation (31). Tubio et al. demonstrated that a few active L1 elements, as identified by 3′ transductions events, show consistent promoter hypomethylation in a variety of tumor types (29). Sanchez-Luque *et al.* found hypomethylation of a L1 donor element in human neurons (36). Together, these studies suggest that L1 promoter methylation status may play a role in distinguishing expressed and unexpressed L1 loci on a broad scale.

Open chromatin allows for transcriptional machinery, including specific transcriptional factors, to access promoter regions. Several transcription factors, such as YY1 (52,53) and RUNX3 (54,55), have been implicated in L1 regulation either experimentally or via identification of predicted transcription factor binding sites (52–54,56–59). While co-factors involved with chromatin remodeling have been found to bind to L1 elements, a role for chromatin state in L1 transcription has not been studied directly (60,61). Chromatin state is influenced by posttranslational modifications of core histones. Histone tails can be methylated and/or acetylated to regulate activation of nearby promoters by recruiting proteins involved in transcriptional regulation (24). Chromatin Immunoprecipitation (CHIP) experiments have shown that both repressive and activating histone marks associate with L1 promoters, though their direct influence on L1 expression has not been studied because none of the published approaches were guided by locus-specific L1 mRNA expression (62–64). The activating histone marks analyzed in connection to L1 expression facilitate transcription through a variety of functions: H3K27Ac and H3K9Ac have roles in chromatin remodeling and facilitating transcriptional elongation (65–68), while H3K27Ac also associates with active enhancers (69). H3K4Me1-3 histone modifications at promoters are associated with regions of open chromatin (70,71). H4K20Me1 is associated with transcription elongation factors (72). H3K4Me3 has been found to localize nearby promoters of active L1Hs-Ta elements in MCF7 cells (32). Bulk analysis of these marks in relation to L1 loci has found that five of these marks, H3K27Ac, H3K9Ac and H3K4Me1-3, are significantly enriched in certain L1 sub-families in the mouse genome (73).

Additional complexity in regulation of expression of L1 loci may arise due to long range interactions between the L1 promoter and enhancers in the general area. Promoter-enhancer associations generally fall within larger DNA loops that are determined by CTCF binding sites (74,75). Alternative DNA loops may occur depending on where CTCF binds, allowing for different loop confirmations and dynamic promoter-enhancer interactions (76). Previous studies have found that CTCF and RNA polymerase II colocalize at the L1 5′ promoter (56). It has been postulated for many years that the L1 promoter is relatively weak (77,78), suggesting a possibility that it may be strengthened by an association with nearby enhancers (56). Interactions between enhancers and L1 elements have only been studied indirectly via enhancer-associated histone marks (73). In combination, all of these epigenetic factors have the potential to silence or activate individual L1 loci. However, whether some of them are more or less critical for efficient expression of endogenous L1 loci remains unknown.

Expression of L1 loci containing intact ORFs as well as those without could have biological implications. L1 loci capable of retrotransposition mobilize themselves and other elements. Based on the current knowledge, the biological impact of expression of L1 loci without intact ORFs could play some cellular role either due to expression or incomplete ORFs (79,80). L1 loci containing intact ORF2p have the potential to generate DNA double strand breaks even in the absence of retrotransposition [80,101]. Additionally, L1 sequences contain a bidirectional promoter, splice sites, and polyadenylation sites, all of which can contribute to generation of chimeric transcripts, interfere with normal gene expression, or drive expression of genomic regions that are otherwise not expressed (81–87).

In this manuscript, we take advantage of our ability to identify individual expressed L1 loci to evaluate epigenetic features distinguishing expressed from transcriptionally silent loci. We focus on L1 loci in MCF7 cells as this cell line has unusually high levels of L1 mRNA expression and the number of expressed L1 loci compared to a much more limited number of expressed L1 loci and low expression levels in other cell lines, such as HeLa and HEK293. We combine a series of studies on epigenetics around expressed vs. unexpressed L1 loci to develop a better understanding of the epigenetic regulation of individual loci expression. Through these efforts, we demonstrate that epigenetic factors can be used for predicting expression of poorly mapped L1Hs loci and that endogenous L1 loci expression can be manipulated in a biologically relevant manner via CRISPR/Cas9 activation and inhibition approaches.

## MATERIALS AND METHODS

### RNA Sequencing and alignment

Cytoplasmic, polyA selected, strand-specific Illumina RNA-Seq (2 × 100 bp) as previously described (22,88) was used for this study (Beijing Genomics Incorporated). Alignment to the hg19 genome and quantification of reads with BOWTIE, bedtools intersect, and manual curation were performed as previously described (22,88). Briefly, this process involves visual inspection of RNA-Seq BAM files in IGV to confirm that reads mapping to a particular L1 element originate from the L1 promoter and not from an upstream promoter. The annotation for the full-length L1 elements present in the reference genome utilized BLAST to identify loci with the L1 promoter region and that intersected with the REPEATMASKER L1 annotation for L1 elements with at least a 5500 bp concordance (22,88). Key factors in the alignment include a requirement that the reads be concordant with the genome as a pair, as the L1 mRNA involved in retrotransposition is not processed. In addition, the ‘tryhard’ setting is used to force the software to look for all possible matches in the genome prior to determining whether one is uniquely better than all others. MCF7 cell line fastq files were downloaded from: https://www.encodeproject.org/experiments/ENCSR000CTU/ as fastq files, ENCFF000HSC and ENCFF000HSK. HEK293 cell line fastq files can be found listed as SRR1275413 from the Gene Expression Omnibus (GEO) traces website. HeLa cell line fastq files are the same files used as the negative control of Figure 10. The unzipped Illumina sequencing fastq file sizes for MCF7, HeLa, and HEK293 are 80.3GB, 40.52GB and 27.54GB, respectively. The total number of mapped reads in MCF7, HeLa and HEK293 BAM files is 30 324 454, 16 581 681 and 7 419 177, respectively. To compensate for these differences, the number of mapped reads for each locus in HeLa and HEK293 were increased by 1.83 and 4.09 times, respectively. We rounded the number of mapped reads for each locus to the nearest whole number and used the adjusted totals for analyses. Expression thresholds were chosen to ensure that the expressed loci are robustly transcribed, the population of expressed loci in MCF7 cells is large enough for rigorous statistical analyses, and that we could consistently compare our analyses across the three cell lines that were sequenced to various depths. This approach was validated by downsampling of the MCF7 file which was performed using the Picard DownsampleSam tool. Biological replicates for RNA-Seq data were performed for all three cell lines using either in-house sequencing for MCF7 and HeLa or existing datasets for HEK293-SRR710092.

### L1 loci association with ATAC peaks

ATAC library preparation and PE sequencing were done by the Tulane Center for Translational Research in Infection and Inflammation NextGen Sequencing core according to their standard protocols seeding 2 million cells. Sequencing reads were mapped using BOWTIE as described for the RNA-Seq data (22,88) and broad peaks were called using MACS2 (89). L1 loci are considered positive for an ATAC-peak association if an ATAC peak overlaps within 500 bp of the L1 5′UTR start site. Biological replicates for ATAC-Seq data were performed for all three cell lines (MCF7 – SRR9684005; HeLa – SRR8171287; HEK293 – SRR6418071).

### L1 loci overlap with genes

Bedtools INTERSECT was used to determine whether each L1 locus overlapped with a gene. Gene coordinates for hg19 were downloaded from the UCSC genome browser (90).

### L1 loci association with activating histone marks

Analysis of activating histone marks associating with individual L1 loci was carried out using bed files downloaded from ENCODE (see below) for each of the CHIP-Seq experiments for activating histone marks. Bedtools INTERSECT was used to identify overlaps between the peaks from the CHIP-Seq and the 5′ end of the full-length L1 annotations. An individual locus is positive for a certain histone mark if the peak overlaps within 500 bp upstream and 300 bp downstream of the L1 5′UTR start site.

### L1 loci association with CpG Methylation

Whole Genome Bisulfite Sequencing (WGBS) using 100 bp paired-end reads was carried out by BGI Global Genomic Services using a DNBseq platform. Bismark-0.20.1 (91) and BOWTIE were used to identify and map methylated/unmethylated CpG islands. Bedtools INTERSECT was used to identify the percentage of methylated CpG islands within the first 500 bp of each L1 locus 5′UTR sequence. Biological replicates for WGBS data were performed for all three cell lines using MCF7 – SRR7707730; HeLa – ENCFF751KHK, ENCFF192ITK; HEK293 – SRR1020524. Total CpGs within the first 500 bp of each L1 5′UTR was assayed with bedtools INTERSECT and a file containing all hg19 CpGs downloaded from: https://figshare.com/articles/All_CpG_sites_for_hg19/1415416/1.

### L1 loci promoter motif sites

Promoter motifs were analyzed based on the canonical motif sequences. The YY1 motif ‘AGCCAAGATGGCCGAATAG’ and RUNX3 motif ‘TGCATTTCCATCTGAGGTA’ used for alignment were derived from the consensus active L1 element (55). All nucleotides in both the YY1 and RUNX3 binding motifs are present in >90% of the L1 loci used to form the consensus. Alignment of loci within the TTC28 gene with the consensus motifs was performed with ClustalW.

### L1 association with CTCF loops, Pol II loops, and DHS linkages

All screenshots of CTCF loops, Pol II loops, and DHS linkages are taken from the following website: http://3dgenome.fsm.northwestern.edu/chiapet.php. CTCF and Pol II loops were identified with Chromatin Interaction Analysis by Paired-End Tag Sequencing (ChIA-PET) (92). Analysis was performed on 10 L1 loci of each group. Limiting the number to 10 loci allowed us to only look at the expressed loci with the most reads and transitional loci with the strongest ATAC peaks. The remaining loci are at most 40% of the expression of the highest ranking locus in the top 10 expressed L1 loci, and therefore we would expect a gradual decrease in data quality.

### Detection of L1Hs loci in MCF7 cells

Paired-end whole genome sequencing of MCF7 cells was obtained from NCBI SRA accession number SRR8652105. The paired alignment files were aligned separately to the human L1 consensus sequence using STAR v2.3.0e and allowing one alignment per read (–outFilterMultimapNmax 1) and a maximum of 25 mismatches (-outFilterMismatchNmax 25). Alignments that occurred in the first 700 bp of the L1 consensus sequence and aligned in the reverse orientation to L1 were extracted. These reads were then used to find their pair based on matching read IDs. The opposite read pair was then aligned to the human genome (hg38) using BOWTIE v0.12.8, requiring unique alignments (-m 1) and allowing three mismatches (-v 3). Alignments in the resulting file were then parsed for read alignments that occurred within the 5′ upstream region of annotated L1Hs loci in hg38. This was done using Bedtools v2.22.0. We cross-referenced the loci detected in MCF7 cells using this method with the 4973 L1 elements from the REPEATMASKER annotation (developed by A.F.A. Smit, R. Hubley and P. Green; see http://www.repeatmasker.org/) analyzed in this manuscript and identified the 246 L1Hs loci present in MCF7 cells analyzed in Figure 8. Liftover (93) was used to convert genomic coordinates of these L1Hs loci from hg38 to hg19.

### Transactivation of L1 mRNA expression in HeLa cells

HeLa cells were cultured in MEM (HyClone) supplemented with 1% sodium pyruvate, l-glutamate, NEAA and 10% FBS as previously described (94). 1.5 × 10^5^ HeLa cells were seeded in each T75 flask 16–18 h prior to transfection. Cells were transfected with 100 ng Cas9m4-VP64 (or Cas9m4-KRAB) (95,96), 100 ng gRNA (either singly or total of a mix), 100 ng of Renilla luciferase- containing plasmid, and 100 ng of plasmid containing the L1 5′ UTR driving Firefly luciferase in the pGL3-basic plasmid (Promega) (83,97). The Renilla luciferase plasmid contains an HSV-tk promoter, a Renilla luciferase reporter, and a polyA signal (pRL-TK, Promega) as a transfection control. Both plasmids and the gRNAs used are illustrated in Figure 9A. Transfection reaction used 12 uL of plus reagent (Invitrogen) and 5 uL of lipofectamine (Invitrogen). All gRNA pools are designed to transfect the same total amount of gRNA plasmid. The exact gRNA sequences used can be found in Additional File 2. The plasmid containing the gRNA targeting AAV (gRNA_AAVS1-T2) was purchased from Addgene (98).

### Alu retrotransposition driven by endogenous L1s

For the Alu retrotransposition assay, experiments were carried out as previously described (80). 1 × 10^6^ HeLa cells were seeded in T75 flasks 16–18 h prior to transfection. Briefly, 1 ug of the Alu reporter plasmid expressing reverse-complementary neo resistance tag (99) was co-transfected with 1 ug plasmid expressing Cas9-VP64 (or Cas9-KRAB) and 1 ug plasmid expressing various gRNAs. After two weeks of G418-selection, colonies were stained and counted. As a control to assess any potential toxicity of our transfection protocols, a plasmid expressing the neo resistance tag (1 ug) was co-transfected with plasmid expressing Cas9-VP64 (or Cas9-KRAB) (1 ug) and a plasmid expressing the gRNA (1 ug). After two weeks of G418-selection, colonies were stained and counted. Transfection reaction used 12 uL of plus reagent (Invitrogen) and 5 uL of lipofectamine (Invitrogen). Workflow for both assays is shown in Supplemental Figure S8B.

### RNA-Seq following stimulation of endogenous L1 mRNA expression

HeLa cells were cultured as previously described in MEM (HyClone) supplemented with 1% sodium pyruvate, l-glutamate, NEAA and 10% FBS as previously described (94). 1 × 10^5^ HeLa cells were seeded in T75 flasks 16–18 h prior to transfection. Cells were co-transfected with 1 ug Cas9-VP64 and 1 ug of pool 2 gRNA expression plasmids using 12 uL of plus reagent (Invitrogen) and 5 uL of lipofectamine (Invitrogen) Cytoplasmic RNA was isolated, polyA-selected and used for PE sequencing as described previously (82). RNA-Seq reads were mapped to Hg19 using BOWTIE and manually curated for authentic expression as described (22,88). The total number of mapped reads from RNA-Seq BAM files in the pool 2 and control HeLa cells is 17 927 082 and 16 581 681, respectively. Biological replicates for pool 2 and control RNA-Seq data were performed.

### Identification L1 elements with intact ORF2

L1 loci with intact ORF2 were determined based on database information of ‘Human Full-Length, Intact LINE-1 Elements’ and ‘Human ORF2 Intact LINE-1 Elements’ from L1Base 2 (100)

## RESULTS

### Locus-specific L1 mRNA expression in MCF7, HeLa and HEK293 cells

**Figure 1.**
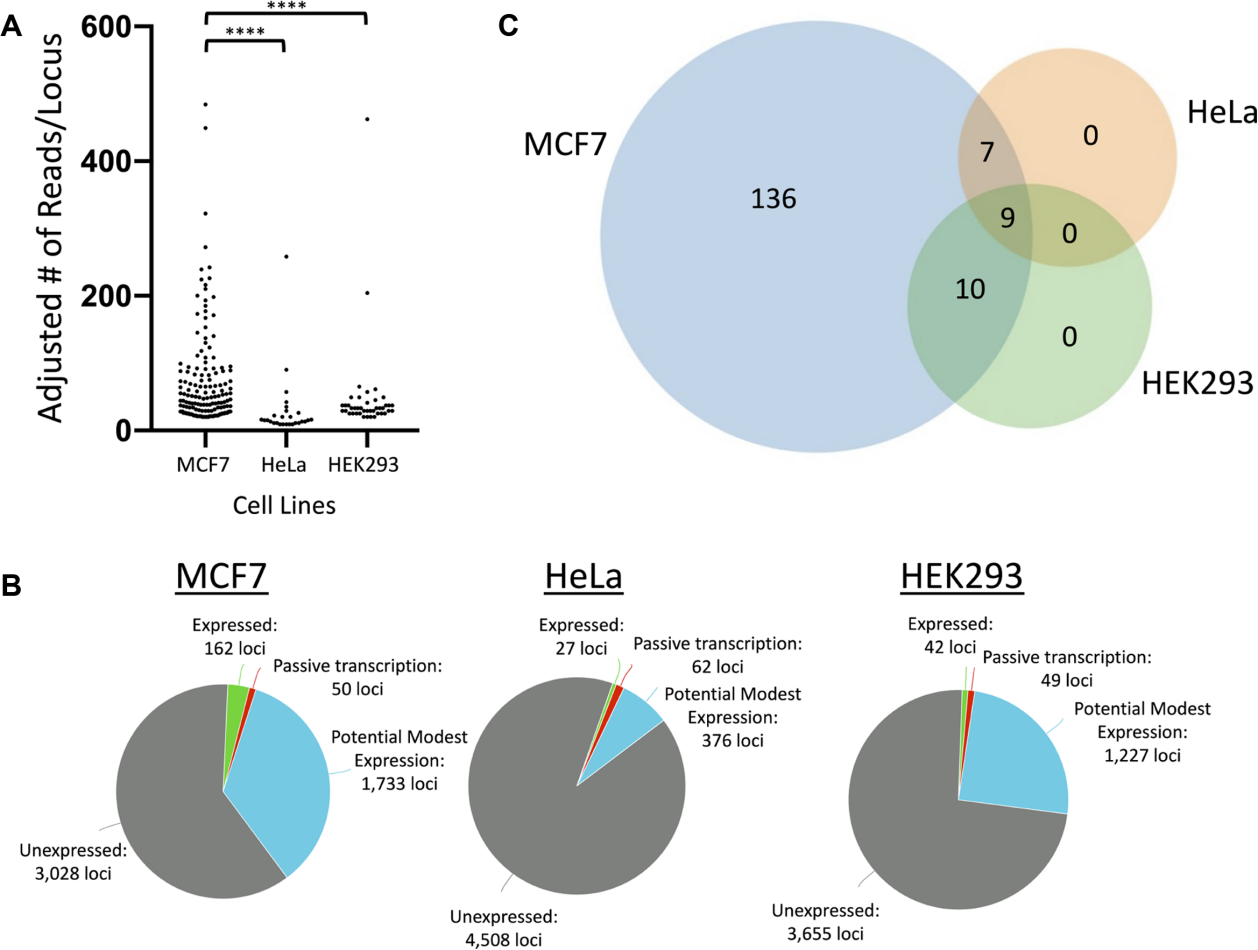
L1 mRNA expression in MCF7, HeLa, and HEK293 cells. (**A**) Reads corresponding to expressed L1 loci as determined by manual curation of PE stranded RNA-Seq are plotted for three different cell lines. The number of mapped reads for each locus in HeLa and HEK293 is adjusted to normalize the differences in total number of mapped reads from each RNA-Seq file (see Materials and Methods). L1 loci with 20 or greater mapped reads (20+) are shown for MCF7 (*n* = 162 loci) and HEK293 (n = 42 loci) cells. Too few loci met this threshold in HeLa cells (*n* = 10), so a threshold of 9+ mapped reads was used instead (*n* = 27 loci) to establish a larger cohort of expressed loci for further analysis. Chi square analysis with Yates’ correction was used to assess the significance of the number of expressed loci with 20+ (9 + in HeLa) mapped reads as a proportion of all L1 loci between cell lines (****P < 0.0001). (**B**) Expression status of L1 loci in MCF7, HeLa, and HEK293 cells as identified by stranded PE RNA-Seq followed by manual validation of resulting mapped reads. Categories of L1 loci for each cell line are: ‘Expressed’ are loci with 20+ mapped reads (9+ in HeLa) and passed manual curation, as described in (A), ‘Passive transcription’ are loci that failed manual curation due to mapped reads that may not have come from the L1 promoter, ‘Potential modest expression’ are loci with 1–19 mapped reads (1–8 in HeLa) that did not undergo manual curation, and ‘Unexpressed’ are loci with 0 mapped reads. (**C**) Expression status of loci expressed in MCF7 (*n* = 162 loci) in MCF7 (blue), HeLa (orange) and HEK293 (green) cells.

Utilizing our RNA-Seq method that involves ‘unique’ alignment of reads combined with manual curation to validate the nature of the transcript (22,88) we find that MCF7 cells have much higher levels of expressed L1 loci than other cells, including HeLa and HEK293 (Figure 1A). Using 20 mapped reads/L1 locus in MCF7 as a cutoff to identify the most robustly expressed L1 loci, we detected 162 of the 4973 full-length L1 loci as being expressed (Additional File, page 1). There were an additional 1,783 loci that either were rejected as not being generated from the L1 promoter during manual validation or whose expression was ambiguous because it was below the 20 read threshold (Figure 1B). Over 3,000 full-length L1 loci had no reads mapping in MCF7 and will be referred to as unexpressed. Downsampling of the MCF7 BAM files to match the total number of mapped reads in HeLa BAM files identified 159 of the 162 loci that are expressed in MCF7 and meeting the expression threshold used for HeLa. Downsampling of the MCF7 BAM files to match the total number of mapped reads in HEK293 BAM files identified 138 loci that are expressed in MCF7 cells meeting the expression threshold used for HEK293. Comparing normalized L1 mRNA expression (see Methods) in HeLa and HEK293 data to MCF7 showed significantly fewer expressed L1 loci in HeLa and HEK293 compared to MCF7 (HeLa: 27 loci, *P* < 0.0001; HEK293: 42 loci, *P* < 0.0001; Chi Square analysis) (Figure 1A). The average levels of reads mapped to a locus expressed in MCF7 (71.2 reads, *n* = 162 loci) was significantly higher than in HeLa (29.8 reads, *P* = 0.005, Student's *t*-test) and near-significant in HEK293 (41.4 reads, *P* = 0.06, Student's *t*-test), although the HeLa results are partially a reflection of the lower threshold of mapped reads for expression (see Methods). We then determined the expression status of the 162 MCF7-expressed loci in HeLa and HEK 293 cells, finding only 16 and 19 of these loci to be expressed in HeLa and HEK293, respectively (Figure 1C). Because of the high number of uniquely expressed loci in MCF7 cells relative to HeLa and HEK293, we proceeded to focus our analysis of epigenetic features of expressed L1 loci on the 162 loci expressed in MCF7 and use L1 loci expressed in both HeLa and HEK293 cell lines for comparative analysis. We also included analysis of total L1 loci in each cell line in our studies because it represents results obtained for bulk analysis of L1 promoters. Evaluation of the location of the 162 L1 loci relative to known genes showed that there was a modest but significant enrichment for expressed compared to unexpressed L1 loci to be within genes (*P* < 0.05, Chi square analysis) (Supplemental Figure S1). This may indicate that genes provide a potentially advantageous chromatin architecture for L1 mRNA expression.

To assess the known L1Hs elements with mutagenic potential, we determined that of the 162 loci identified to be expressed in MCF7 cells, 2 loci contain intact ORF2, both of which are L1Hs elements and includes L1-5219 (Additional File, page 1). L1 loci containing intact ORF2p have the potential to generate DNA double strand breaks even in the absence of retrotransposition (80,101). We also analyzed 38 L1 elements previously assessed for their retrotransposition potential (as identified by Rodriguez-Martin *et al.* (102)) and found three loci had 14–26 mapped reads and 4 more had only 1–6 reads and are therefore potentially background level of mapping in MCF7 (Additional File, page 4). There were also similar number of loci from these 38 loci expressed in HEK293 and HeLa cells.

### Promoters of most expressed L1 loci overlap with an ATAC peak

**Figure 2.**
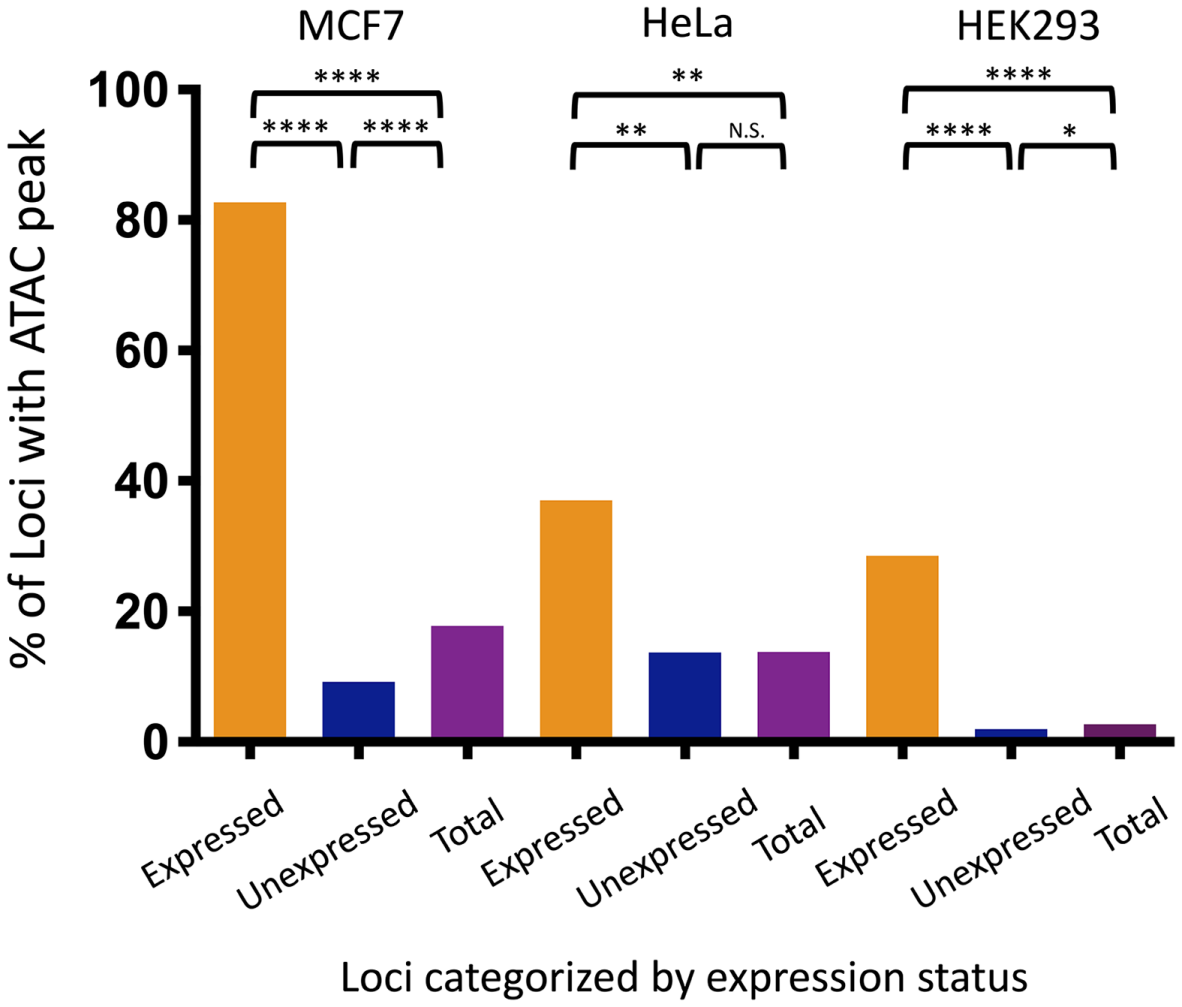
Analysis of ATAC peaks at expressed and unexpressed L1 loci. 82.72% of the 162 L1 loci expressed in MCF7 cells have ATAC peaks around their promoters as compared to 9.25% of the 3,028 unexpressed loci and 17.76% of the 4,973 total loci. 37.04% of the 27 L1 loci expressed in HeLa cells have ATAC peaks as compared to 13.71% of the 4,508 unexpressed loci and 13.75% of the 4,973 total loci. 28.57% of the 42 L1 loci expressed in HEK293 cells have ATAC peaks as compared to 1.94% of the 3,655 unexpressed loci and 2.67% of the 4973 total loci. Chi square analysis with Yates’ correction was used to determine significance (*P < 0.05; **P < 0.01; ****P < 0.0001).

Chromatin accessibility measured by ATAC-Seq (103) identified peaks at 89,503, 67,953 and 19,216 locations throughout the genomes of MCF7, HeLa and HEK293, respectively. The majority of L1 loci expressed in MCF7 had an ATAC peak overlapping with their promoter (134/162 L1 loci, 83%), while only 280 out of 3,028 unexpressed L1 loci (9%) had ATAC peaks (*P* < 0.0001, Chi square analysis) (Figure 2). Some of these 280 loci with ATAC peaks but no L1 mRNA expression may represent a transitional state that has either lost or not yet gained a key component required for detectable expression. We will refer to these L1 loci as ‘transitional’ loci. The L1 loci with mapped reads that primarily originated from a non-L1 promoter showed only 14 of 50 loci (28%) having an ATAC peak, a significant decrease in ATAC peak association as compared to the 162 expressed loci (*P* < 0.0001, Chi square analysis). It is possible that some of these loci express at a low level that is indistinguishable from the surrounding passive transcription. Analyses of ATAC peaks in HeLa and HEK293 show a significant increase in ATAC peaks detected at expressed versus unexpressed L1 loci in both HeLa (37.04% versus 13.71% of loci, *P* < 0.01, Chi square analysis) and HEK293 (28.57% versus 1.94%, *P* < 0.0001, Chi square analysis) (Figure 2). Thus, in all three cell lines, MCF7, HeLa, and HEK293, ATAC peaks are significantly more likely to overlap with expressed as opposed to unexpressed loci. When we evaluate the presence of ATAC peaks at all L1 elements regardless of their expression status, we see many shared loci, as well as cell-line specific loci (Supplemental Figure S2). Only 49 out of the 1,325 loci having an ATAC peak in at least one cell line were found in common between all three cell lines. These findings are generally consistent with the cell-line specificity of L1 expression (Figure 1).

### Activating histone marks are enriched at promoters of expressed L1 loci

**Figure 3.**
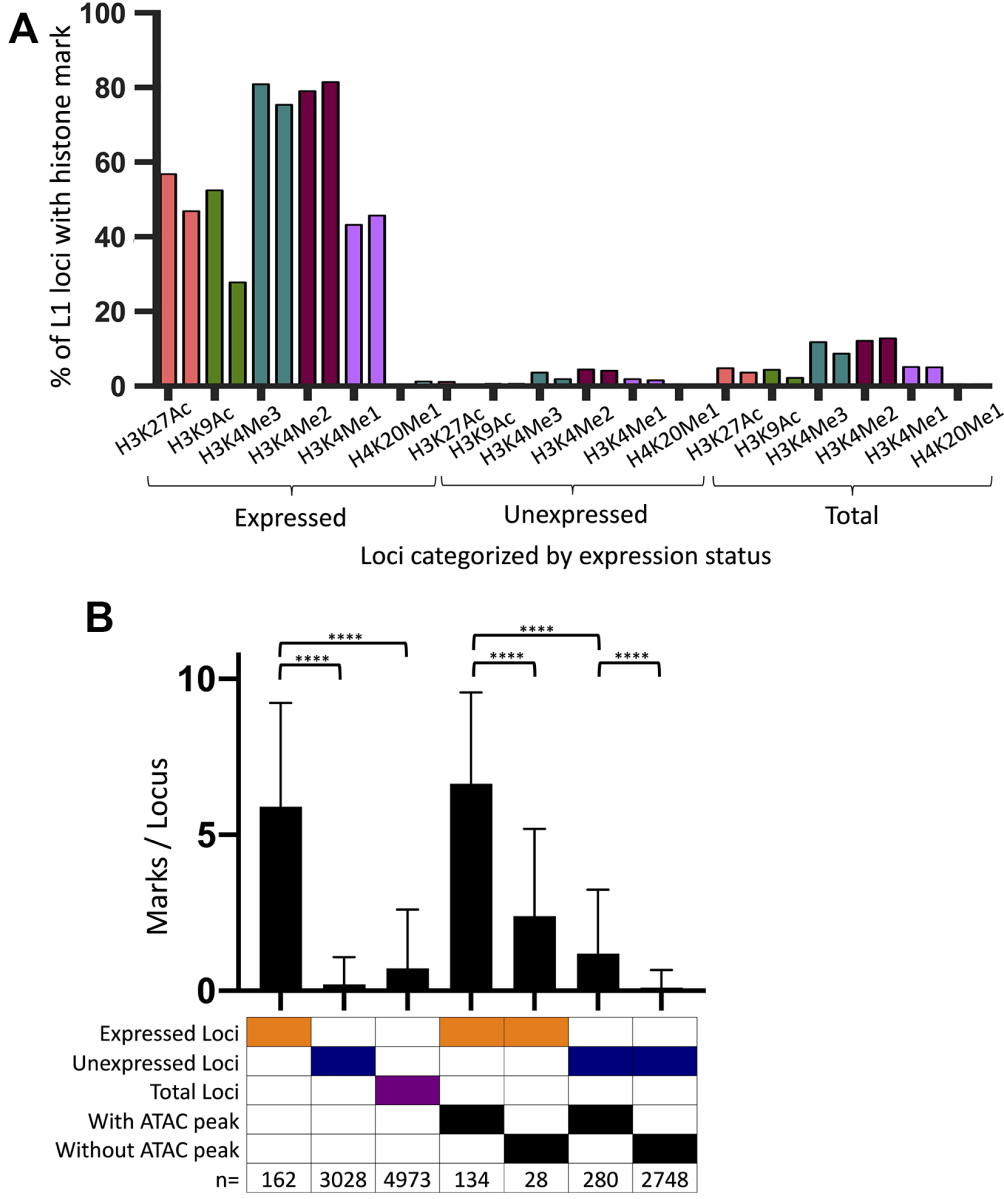
Activating histone marks present in the L1 promoter distinguish expressed from unexpressed L1 loci. (**A**) Percent of L1 loci containing peaks for each activating histone mark found in promoters of expressed (*n* = 162), unexpressed (*n* = 3,028), and total loci (*n* = 4,973) in MCF7 cells. Two experiments are shown for each histone mark. The percent of H3K27Ac, H3K9Ac, H3K4Me3, H3K4Me2 and H3K4Me1 histone marks present at expressed L1 loci is significantly higher compared to their percent at unexpressed and total L1 loci (Chi square analysis with Yates’ correction, *P* < 0.0001). (**B**) L1 loci with ATAC peaks have more activating histone marks than L1 loci without ATAC peaks in MCF7 cells. The average number of activating histone marks is calculated out of 12 marks (the 6 marks analyzed in two experiments) for expressed loci (*n* = 162), unexpressed loci (*n* = 3,028), total loci (*n* = 4,973), expressed loci with an ATAC peak (*n* = 134), expressed loci without an ATAC peak (*n* = 28), unexpressed loci with an ATAC peak (*n* = 280), unexpressed loci without an ATAC peak (*n* = 2,748). Significance between different groups of L1 loci was determined by Student's *t*-test (****p < 0.0001).

The open chromatin that is associated with transcription generally contains specific histone modifications (104,105). We analyzed expressed, unexpressed, and total L1 loci for the presence of activating histone marks H3K27Ac, H3K9Ac, H3K4Me3, H3K4Me2, H3K4Me1 and H4K20Me1 using ENCODE CHIP-Seq data sets performed in duplicate using DNA/protein extracts from MCF7, HeLa and HEK293 cells (see Materials and Methods). Only data concerning H3K27Ac, H3K4Me3 and H3K4Me1 status were available for HEK293 cells. This analysis determined that five of the six histone marks, H3K27Ac, H3K9Ac, H3K4Me3, H3K4Me2 and H3K4Me1, are significantly enriched (*P* < 0.0001, Chi square analysis) at promoters of L1 loci expressed in MCF7 cells as compared to unexpressed loci (Figure 3A). Of the six activating histone marks analyzed, the marks most commonly present at expressed L1 loci are H3K4Me3 (78.09% of loci) and H3K4Me2 (80.25%). We also analyzed activating histone marks at promoters of expressed, unexpressed, and total L1 loci in HeLa and HEK293 cells. Similar to findings in MCF7, there was a significant difference in the presence of the H3K27Ac, H3K9Ac, H3K4Me3, H3K4Me2 and H3K4Me1 histone marks at the promoters of expressed versus unexpressed loci in HeLa cells (*P* < 0.0001, Chi Square analysis) (Supplemental Figure S3A). In HEK293 cells, a significant difference was seen for all three histone marks analyzed (H3K27Ac, H3K4Me3 and H3K4Me1) between expressed versus unexpressed loci (Supplemental Figure S3B). A significant difference was also seen when comparing the number of activating histone marks per locus of expressed and unexpressed loci in MCF7 (5.91 marks versus 0.21 marks, *P* < 0.0001, Student's *t*-test) (Figure 3B), HeLa (2.85 marks versus 0.19 marks, *P* < 0.0001, Student's *t*-test), and HEK293 cells (0.9 marks versus 0.05 marks, *P* < 0.0001, Student's *t*-test) (Supplemental Figure S3C). In addition, HeLa cells have fewer overall of these activating marks per locus regardless of its expression status than do MCF7 cells (Supplemental Figure S3D, left). When analyzing only the H3K27Ac, H3K4Me3 and H3K4Me1 histone marks, both HeLa and HEK293 cells contained fewer overall of these activating marks per L1 locus regardless of its expression status than did MCF7 (Supplemental Figure S3D, right). These findings are consistent with the relatively high level of L1 mRNA expression detected in MCF7 as opposed to HeLa and HEK293 cells (Figure 1A). This is supported by the finding that in MCF7 cells there was a correlation between L1 mRNA expression levels and the number of activating histone marks that were detected at individual expressed L1 loci (*P* = 0.017, *R*^2^ = 0.035, Pearson correlation test) (Supplemental Figure S3E).

In addition to the significantly higher levels of activating histone marks associated with expressed versus unexpressed L1 loci in MCF7 cells (Figure 3A, B), we observe a significant difference between the number of activating marks per expressed L1 locus that have an ATAC peak versus the number of activating marks per expressed L1 locus without an ATAC peak (Figure 3B). There is also a subset of unexpressed L1 loci that have an ATAC peak and significantly higher number of activating histone marks than the unexpressed L1 loci with no ATAC. These are the loci that we previously designated as ‘transitional’ (Figure 2). The lower number of activating histone marks at these loci would be consistent with an incompletely assembled transcriptional apparatus.

### CpG methylation of L1 promoters is associated with lower L1 mRNA expression

**Figure 4.**
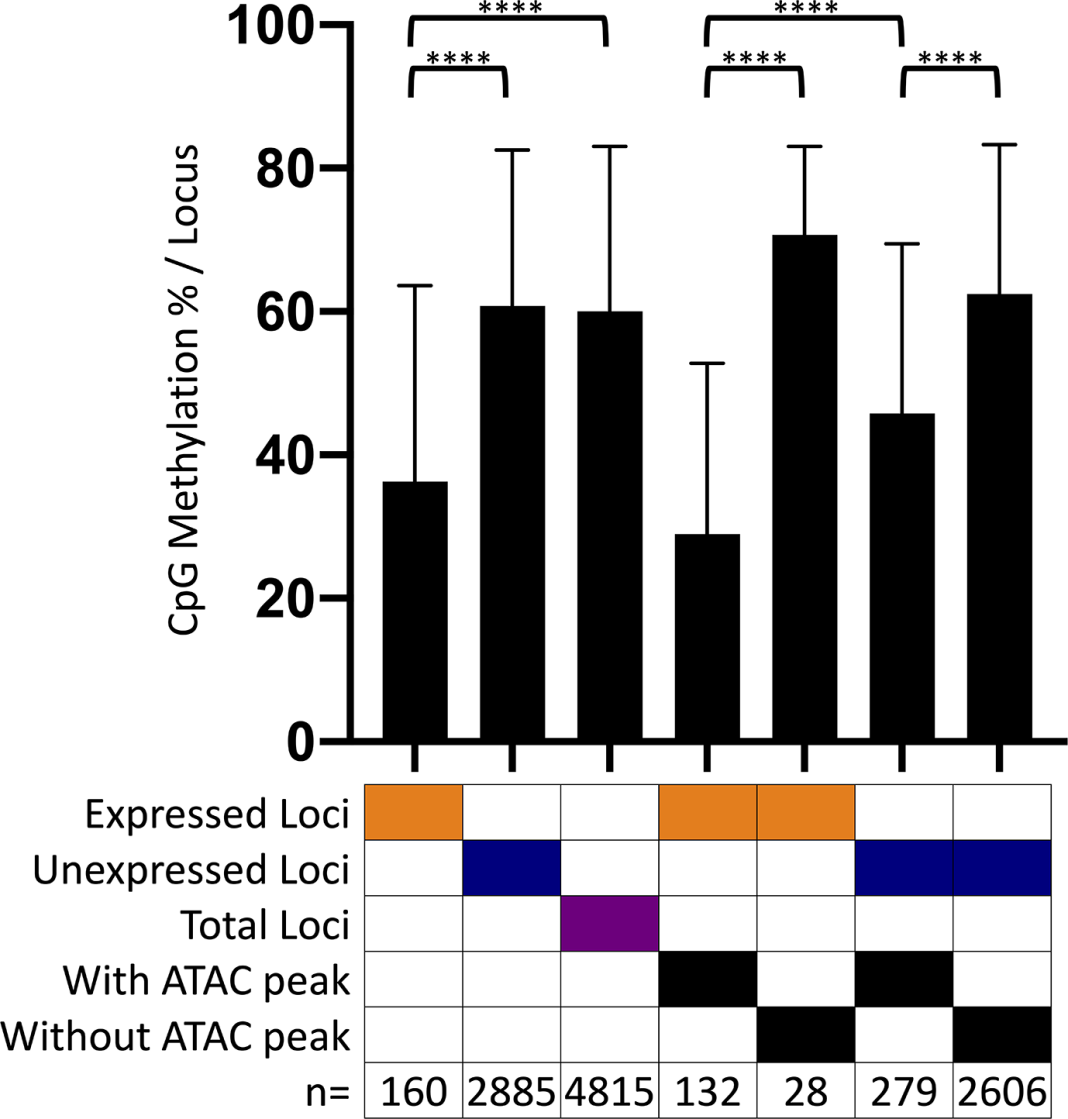
Promoters of L1 loci expressed in MCF7 cells are hypomethylated. Analysis of CpG methylation at promoters of expressed loci (*n* = 160, orange), unexpressed loci (*n* = 2,885, blue), total loci (*n* = 4,815, purple), expressed loci with an ATAC peak (*n* = 132), expressed loci without an ATAC peak (*n* = 28), unexpressed loci with an ATAC peak (*n* = 279), and unexpressed loci without an ATAC peak (*n* = 2,606). A total of 158 loci were unmapped following bisulfite sequencing and thus excluded from this analysis. Significance between different groups of L1 loci was determined by Student's *t*-test (****P < 0.0001).

We performed bisulfite sequencing using genomic DNA extracted from MCF7, HeLa, and HEK293 cells to use NGS sequencing to determine the methylation status of all CpGs in these three cell lines and specifically for L1 loci. L1 loci expressed in MCF7 cells have a lower percentage of methylated CpGs mapping to the L1 promoter region (36.26% of methylated CpGs) compared to either unexpressed loci (60.82%) or total loci (60.01%) (*P* < 0.0001, Student's *t*-test) (Figure 4). However, we found no significant difference between methylation at expressed versus unexpressed loci in HeLa (42.85% versus 45.99%, *P* = 0.55, Student's *t*-test) and HEK293 cells (41.3% versus 45.14%, *P* = 0.37, Student's *t*-test) (Supplemental Figure S4A). The significant hypomethylation status of expressed L1 loci in MCF7 cells, compared to HeLa and HEK293, contradicts the higher levels of overall methylation within the MCF7 genome compared to the other cells (Supplemental Figure S4B) as well as higher levels of L1 loci methylation in MCF7 cells when all L1 loci are considered as they are in bulk analysis of L1 methylation (Supplemental Figure S4C). Additionally, in MCF7 cells, loci with an ATAC peak have significantly lower levels of CpG methylation than loci of the same expression status without an ATAC peak (Figure 4). Of particular note is the observation that the ‘transitional’ loci (unexpressed loci with an ATAC peak) showed significantly lower methylation levels than unexpressed L1 loci without an ATAC peak (Figure 4). Thus, these transitional loci have both lower promoter methylation (Figure 4) and a higher number of activating marks than unexpressed L1 loci without ATAC peaks (Figure 3).

### Analysis of the epigenetic status of L1 loci expressed in MCF7 cells in HeLa and HEK293 cells

**Figure 5.**
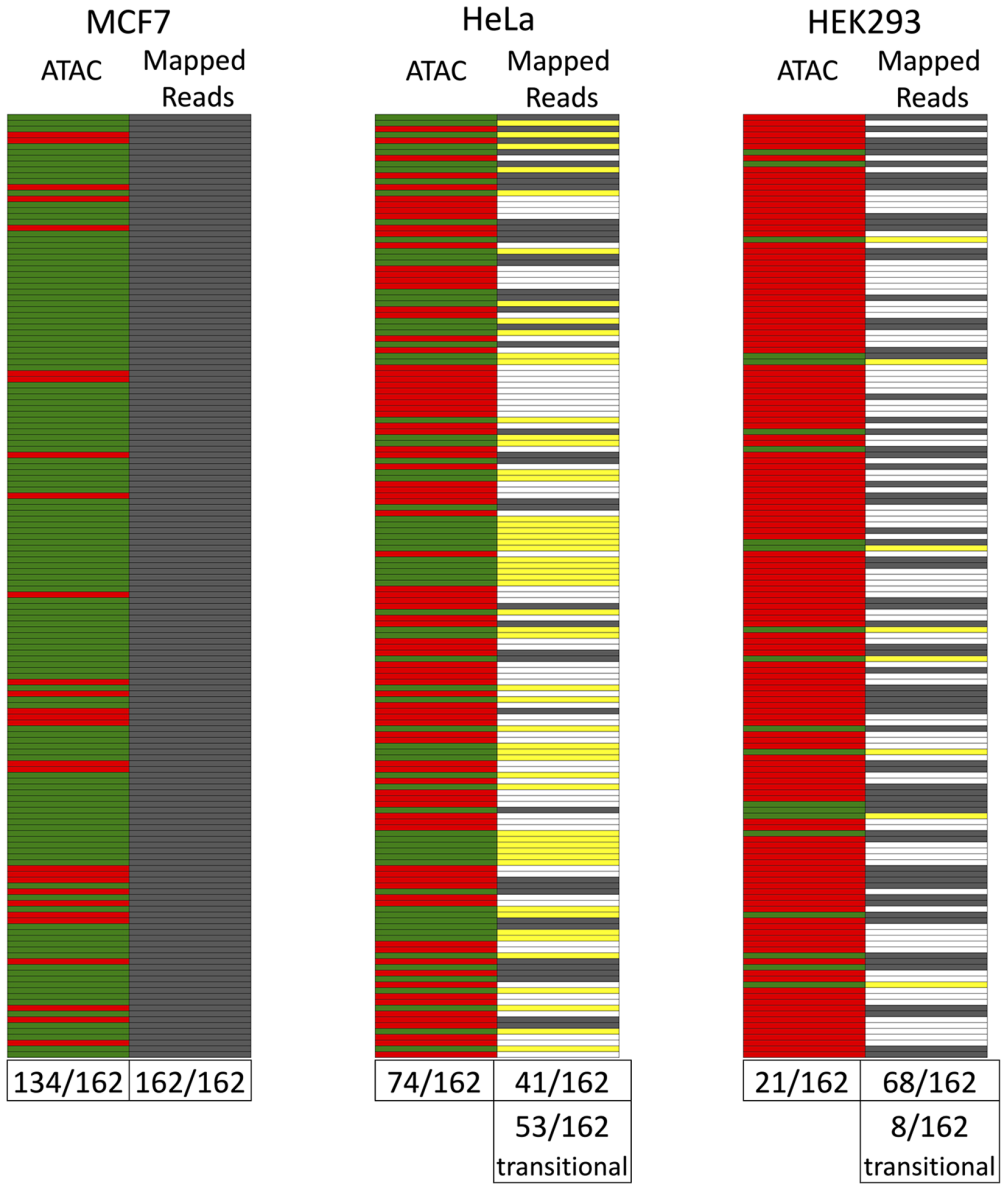
ATAC and expression status of the 162 L1 loci that are expressed in MCF7 cells within HeLa and HEK293 cells. Each locus expressed in MCF7 cells (n = 162) is evaluated for their ATAC status in MCF7 cells, and for ATAC and presence of mapped reads (1+ mapped read) status in HeLa and HEK293 cells. Presence of an ATAC peak (green) or with an absence of an ATAC peak (red) and presence (grey) or absence (white) of mapped reads at each locus is shown for each cell line. ‘Transitional’ loci (unexpressed loci with an ATAC peak) are marked in yellow. Numbers below each respective column indicate the number of loci out of the 162 loci evaluated that have an ATAC peak or have mapped. The number of loci categorized as ‘transitional’ is listed at the bottom of the ‘Mapped Reads’ column for HeLa and HEK293. Loci are shown in the same order in each cell line and ordered from highest (top) to lowest (bottom) number of mapped RNA-Seq reads as determined in MCF7 cells.

Due to the significantly different expression patterns between MCF7, HeLa and HEK293 cells (Figure 1), we hypothesized that there should be associated epigenetic changes at loci exhibiting cell-line specific expression. We used the 162 loci robustly expressed in MCF7 cells as the standard and compared whether there were any reads mapped to the same loci in HeLa or HEK293 cells (Figure 5, heat map, grey). Of these 162 loci expressed in MCF7 cells, only 41 and 68 loci had any reads mapped to them in HeLa and HEK293, respectively, with a total of 885 and 1,580 reads mapped to these 162 loci in each cell line compared to 11,534 mapped reads in MCF7 (Figure 5). We found that five of the six activating histone marks analyzed in HeLa cells (H3K27Ac, H3K9Ac, H3K4Me3, H3K4Me2 and H3K4Me1) and all three marks analyzed in HEK293 (H3K27Ac, H3K4Me3 and H3K4Me1) were able to distinguish between the loci expressed in MCF7 relative to those unexpressed in MCF7 when analyzed in HeLa and HEK293, respectively (Supplemental Figure S5A, B). The percent of loci containing these marks in Hela and HEK293 cells is significantly lower than in MCF7 cells, consistent with the finding that only some of the 162 L1 loci expressed in MCF7 cells are also expressed in HeLa and HEK293 cells (Figure 5). In HEK293 cells, methylation is significantly higher in expressed loci compared to unexpressed loci (Supplemental Figure S5C), the opposite of what was observed in MCF7 cells (Figure 4), while no significant difference is seen in HeLa cells (Supplemental Figure S5C). Similar to the consistency seen with activating histone marks, as many as 69 out of the 162 loci have shared ATAC peaks between MCF7 and HeLa, with 15 loci in common to all three cells lines (Figure 5, green; Supplemental Figure S5D). Many of the shared L1 loci with ATAC peaks fit into our definition of ‘transitional’ elements that have ATAC peaks showing some open chromatin but no mapped reads (Figure 5, yellow).

### Long-distance genomic interactions contribute to expression of L1 elements

**Figure 6.**
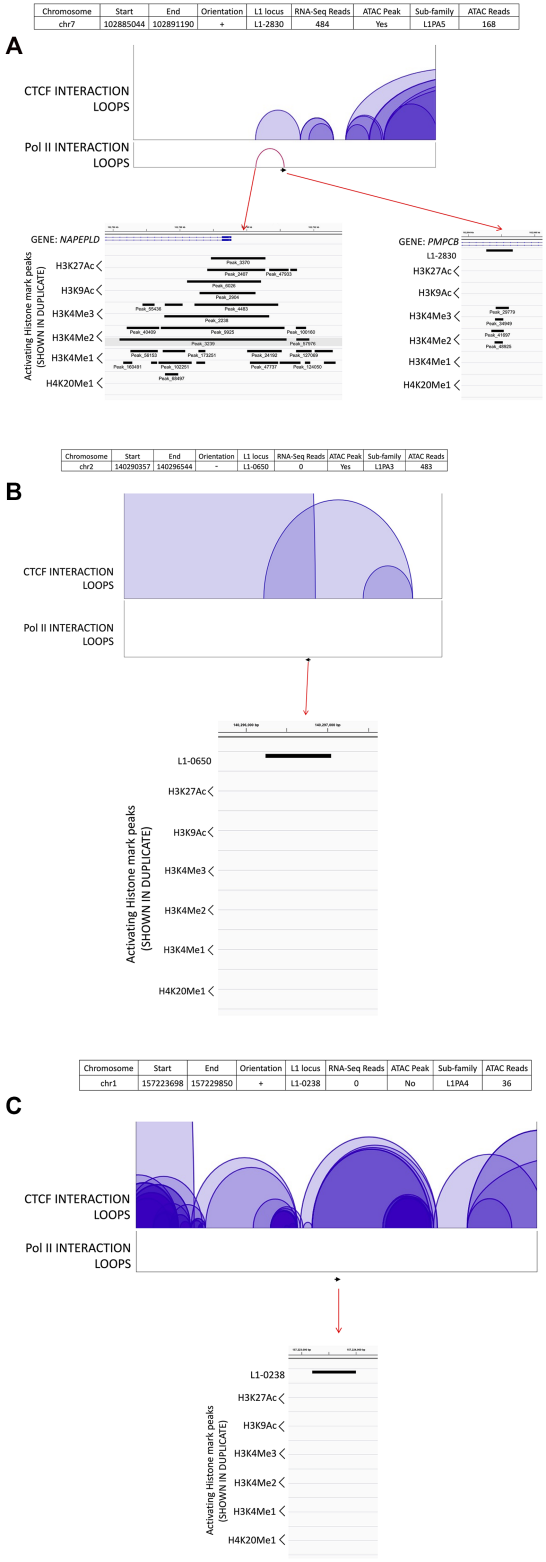
Long-distance interactions of specific L1-loci in MCF7 cells. Screenshots of Integrated Genomics Viewer of L1-2830 (**A**), L1-0650 (**B**) and L1-0238 (**C**) in MCF7 cells. L1-2830 is an expressed locus with the most mapped RNA-Seq reads in MCF7. L1-0650 is a ‘transitional’ locus, i.e. unexpressed locus with the second most ATAC-sequencing reads mapped. L1-0238 is a randomly-selected unexpressed locus. Information regarding the locus location (chromosome, start site, end site and orientation), RNA-Seq reads, presence of an ATAC peak, L1 sub-family, and number of reads from ATAC sequencing is shown in a table format at the top. Loops indicate CTCF binding sites (purple, shaded) within 500 kb of the L1 start site (black arrow). RNA polymerase II (Pol II) loops (red) are only shown if the pol II binding site overlaps within 500 bp of the L1 start site. Red arrows indicate magnification of the indicated genomic region that includes analysis of the activating histone marks from previously described CHIP-Seq data (Figure 3A). The status of each histone mark is examined in two experiments.

Three dimensional (3D) chromatin interactions can strongly influence transcriptional state and regulation (106). We utilized ChIA-Pet conformation studies in MCF7 to determine whether 3D interactions may explain why some L1 elements are expressed and others are not. The CHIA-PET data (107) analyzed 3D interactions involving RNA polymerase II (pol II) binding that was associated presumably with both the promoter and an enhancer, while also analyzing CTCF binding to determine whether cohesin/CTCF loops might establish Topologically Associated Domains (TADs) (108,109) that defined which elements are able to interact with an enhancer. We compared genomic interactions of the ten highest expressed loci in MCF7 (Table 1 and Figure 6A, Supplemental Figure S6A–I), 10 transitional loci (unexpressed loci overlapping with an ATAC peak) (Table 1 and Figure 6B, Supplemental Figure S6J–R), and ten randomly chosen unexpressed loci (Figure 6C, Supplemental Figure S6S-AA). We show that nine out of ten expressed loci have their promoter closely associated with a distant sequence via RNA pol II binding (Table 1 and Figure 6A, Supplemental Figure S6A-H). This would be consistent with the L1 promoter interacting with an enhancer (Table 1). We also see that seven of these promoter/enhancer interactions are present within a CTCF loop (Figure 6A, Supplemental Figure S6A–C, E, G, H). Analysis of the ‘transitional’ L1 loci shows that only four of these loci associate with an RNA pol II loop and that all four of these loci are within a CTCF loop (Table 1, Supplemental Figure S6J–L, Q). In contrast, of the ten unexpressed loci (Figure 6C, Supplemental Figure S6S-AA), only one locus (Supplemental Figure S6Y) shows an interaction loop with the RNA pol II.

**Table 1.** Summary of Long-distance interactions of 10 expressed, transitional, and unexpressed L1-loci in MCF7 cells. Data regarding 3D chromatin interactions for all expressed, transitional, and unexpressed L1 loci analyzed in Figure 6 and Supplemental Figure S6 is summarized. The data listed for each L1 locus (L1 locus column) includes number of mapped RNA-Seq reads (Reads column), status of an ATAC peak (ATAC peak column), position within a CTCF loop (CTCF loop column), RNA pol II association (pol II loop column), number of activating histone marks overlapping the L1 locus (histone marks on L1 column), and number of activating histone marks overlapping with the putative enhancer region (histone marks on enhancer column)

**Expressed loci**							
**Figure**	**L1 locus**	**Reads**	**ATAC peak**	**CTCF loop**	**Pol II loop**	**Histone marks on L1**	**Histone marks on enhancer**
6A	L1-2830	484	Yes	Yes	Yes	4	11
Supp. 6A	L1-0728	449	Yes	Yes	Yes	9	0
Supp. 6B	L1-3682	322	Yes	Yes	Yes	7	11
Supp. 6C	L1-1867	272	Yes	Yes	Yes	8	11
Supp. 6D	L1-2476	242	Yes	No	Yes	0	10
Supp. 6E	L1-3165	239	Yes	Yes	Yes	10	0
Supp. 6F	L1-0029	226	Yes	No	Yes	9	10
Supp. 6G	L1-3455	224	Yes	Yes	Yes	10	12
Supp. 6H	L1-1685	216	Yes	Yes	Yes	5	11
Supp. 6I	L1-3239	210	Yes	No	No	9	No Enhancer (0)
	AVERAGE	288.4	Yes (10/10)	Yes (7/10)	Yes (9/10)	7.1 (STDEV: 3.2)	7.6 (STDEV: 5.3)
**Transitional loci**							
**Figure**	**L1 locus**	**Reads**	**ATAC peak**	**CTCF loop**	**Pol II loop**	**Histone marks on L1**	**Histone marks on enhancer**
6B	L1-0650	0	Yes	Yes	No	0	No Enhancer (0)
Supp. 6J	L1-2855	0	Yes	Yes	Yes	6	10
Supp. 6K	L1-0225	0	Yes	Yes	Yes	5	12
Supp. 6L	L1-0986	0	Yes	Yes	Yes	0	3
Supp. 6M	L1-4910	0	Yes	Yes	No	5	No Enhancer (0)
Supp. 6N	L1-5151	0	Yes	Yes	No	4	No Enhancer (0)
Supp. 6O	L1-1469	0	Yes	Yes	No	1	No Enhancer (0)
Supp. 6P	L1-0482	0	Yes	Yes	No	1	No Enhancer (0)
Supp. 6Q	L1-3525	0	Yes	Yes	Yes	9	0
Supp. 6R	L1-4180	0	Yes	Yes	No	2	No Enhancer (0)
	AVERAGE	0	Yes (10/10)	Yes (10/10)	No (4/10)	3.3 (STDEV: 3)	2.5 (STDEV: 4.6)
**Unexpressed Loci**							
**Figure**	**L1 locus**	**Reads**	**ATAC Peak**	**CTCF loop**	**Pol II loop**	**Histone marks on L1**	**Histone marks on Enhancer**
Fig. 6C	L1-0238	0	No	Yes	No	0	No Enhancer (0)
Supp. 6S	L1-0518	0	No	Yes	No	0	No Enhancer (0)
Supp. 6T	L1-1501	0	No	Yes	No	0	No Enhancer (0)
Supp. 6U	L1-1960	0	No	Yes	No	0	No Enhancer (0)
Supp. 6V	L1-4228	0	No	Yes	No	0	No Enhancer (0)
Supp. 6W	L1-4249	0	No	Yes	No	0	No Enhancer (0)
Supp. 6X	L1-4821	0	No	Yes	No	0	No Enhancer (0)
Supp. 6Y	L1-4938	0	No	Yes	Yes	0	0
Supp. 6Z	L1-5330	0	No	Yes	No	0	No Enhancer (0)
Supp. 6AA	L1-5742	0	No	Yes	No	0	No Enhancer (0)
	AVERAGE	0	No (0/10)	Yes (10/10)	No (1/10)	0 (STDEV: 0)	0 (STDEV: 0)

Analysis of active histone marks shows that seven of the ten expressed L1 loci discussed above are associated with enhancer-like genomic regions that contain high levels of activating histone marks (Table 1, Figure 6A, Supplemental Figure S6B–D,F–H). All but one of these loci have significant levels of activating histone marks on their promoter as well (Table 1, Figure 6A, Supplemental Figure S6B,C,F–H). Thus, all of the expressed loci have significant levels of activating histone marks associated either through their promoter or their enhancer and most have both (Figure 6A, Supplemental Figure S6A–I). Of the ten transitional loci analyzed, only three associate with a potential enhancer containing histone marks through RNA pol II (Table 1, Supplemental Figure S6J-L) and generally have much lower levels of histone activation on both their promoter and enhancer regions (Expressed: 7.1 and 7.6 marks, respectively; Transitional: 3.3 and 2.5 marks, respectively) (Table 1). These loci contrast with the ten random, unexpressed loci where only one has a putative RNA pol II-mediated loop (Supplemental Figure S6Y), but that enhancer and none of the promoters are associated with activating histone marks (Table 1, Figure 6C, Supplemental Figure S6S-AA).

### Selective expression of the L1-5219 locus located within the *TTC28* gene

**Figure 7.**
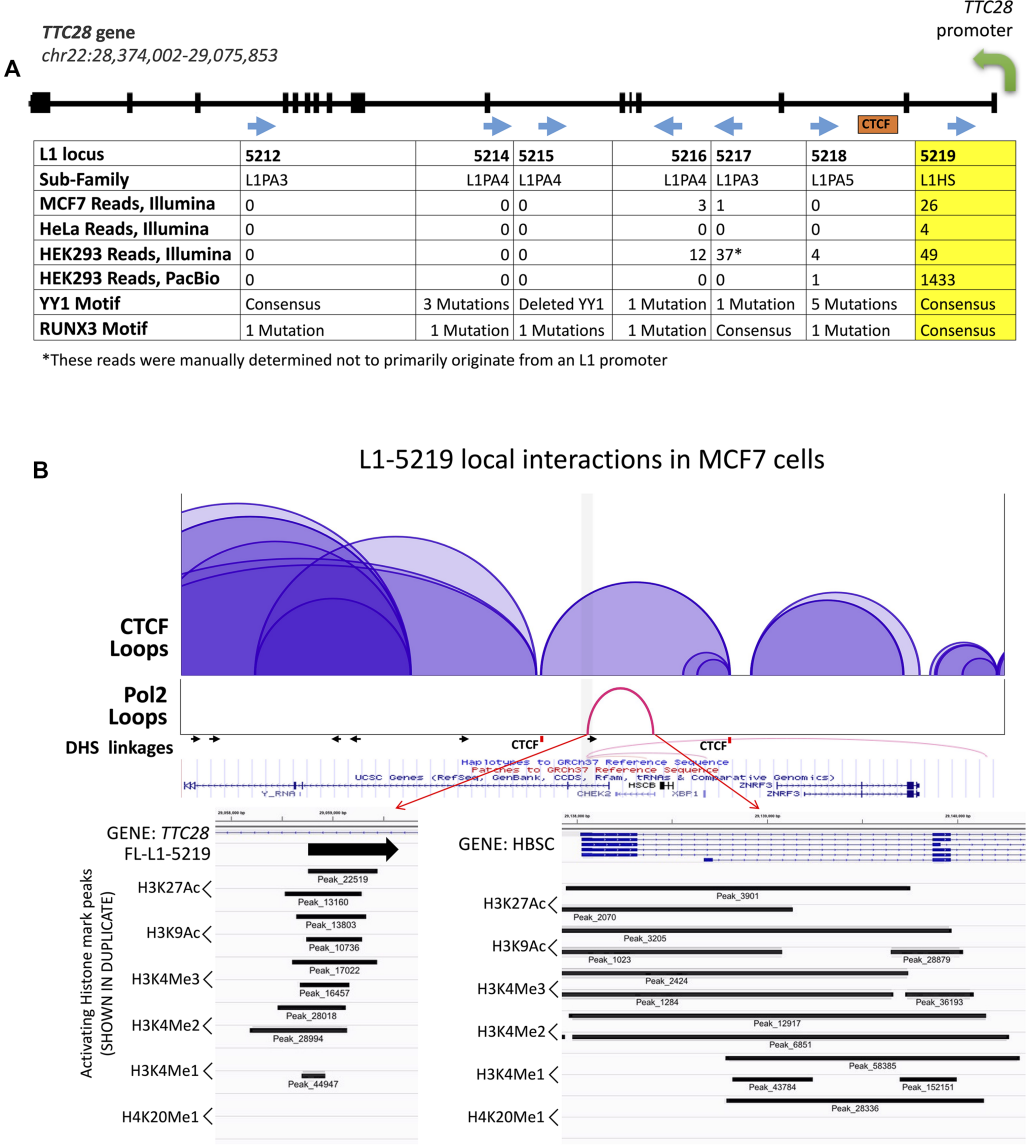
Comparative analysis of full-length L1 loci identified within the *TTC28* gene. (**A**) Schematic of the *TTC28* gene (black) and exons (heavy black bars) transcription start site (green arrow), seven full-length L1 loci (blue arrows reflecting L1 orientation relative to the gene), and a CTCF binding site (orange box). The chart below the schematic contains information regarding each of the seven loci: L1-5212, L1-5214, L1-5215, L1-5216, L1-5217, L1-5218 and L1-5219. The information provided is L1 sub-family, RNA-Seq reads for expressed loci (Illumina or PacBio technologies (22)), and sequence status of YY1 and RUNX3 binding sites (55). Reads mapped to L1-5217 in HEK293 cells were manually determined not to primarily originate from an L1 promoter. (**B**) 3D interactions and epigenetic marks identified at L1 loci within the *TTC28* gene in MCF7 cells. Loops indicate CTCF binding sites (purple, shaded) within 500 kb of the L1-5219 start site (black arrow). RNA polymerase II (Pol II) loops (red) are only shown if the pol II binding site directly overlaps with an L1 start site. DNAse Hypersensitivity Site (DHS) linkages (light red lines) and the surrounding genomic sequences (blue) are shown as well (92). A CTCF binding site 5′ of the L1-5219 is marked in red. Red arrows indicate magnification of the L1-5219 element and the associated enhancer region, respectively, showing the presence of activating histone marks at each genomic region as determined by previously described CHIP-Seq data (Figure 3A). The status of each histone mark is shown from two experiments.

Analysis of L1 loci within the *TTC28* gene allowed exploration of reasons underlying differential expression of L1 loci that are present near one another in the human genome. This gene is located on chromosome 22 and harbors seven intronic, full-length L1 elements (Figure 7A). One locus, L1-5219, has been previously characterized as an unusually active locus that is expressed in multiple cancers (21,22,29,30,32,64,110). L1-5219 shows consistent mRNA expression across multiple cancer cell lines with 26 mapped reads in MCF7 cells, 4 mapped reads in HeLa cells, and 49 mapped reads in HEK293 cells, despite the very low mappability of this element. The higher expression of L1-5219 as compared to the other 6 loci within *TTC28*, which all show little or no authentic expression, makes them a suitable model for identification of factors affecting their expression.

L1-5219 is the only member of the L1Hs-Ta sub-family, the youngest and most retrotranspositionally active L1 subfamily in this gene (Figure 7A). Analysis of the promoter sequences of all seven loci showed that one locus, L1-5215, has a deleted YY1 motif and only two loci, L1-5212 and L1-5219, contains the consensus relative to the motif from the consensus active L1 sequence (55). Analysis of the RUNX3 binding motif determined that two L1 loci, L1-5219 and L1-5217, have the consensus sequence, while the other five contain one mutation in this site (Figure 7A) (55). The impact that these mutations may have on a specific L1 locus expression is unknown.

Chromosomal locations (hg19) and analysis of the seven L1 loci in the TTC28 gene for chromatin accessibility, activating histone marks, and percentage of methylated CpGs (Supplemental Table S1) determined that the promoter region of L1-5219 associates with an ATAC peak in all three cell lines (MCF7, HeLa, and HEK 293) while the other loci do not overlap with an ATAC peak in any cell line. Additionally, despite having the highest number of CpGs (31) in its promoter sequence, L1-5219 has the lowest percentage of methylated CpGs in MCF7 (0%), HeLa (14.29%), and HEK293 cells (18.33%). L1-5219 has a variable percentage of activating histone marks: 33.33% in HEK293, 75% in HeLa and 75% in MCF7 cells. Some of the lack of histone activation mark detection may represent poor mappability in the vicinity of the L1-5219 promoter.

3D interactions in the *TTC28* region using CHIA-PET data show direct RNA pol II interactions only with L1-5219 promoter that connects this locus with a putative enhancer (Figure 7B). The associated enhancer is approximately 75 kb away, between two other genes. This RNA pol II loop is fully within a CTCF loop mediated by a CTCF binding site located within the intronic region of *TTC28* 62 kb upstream of L1-5219. None of the other TTC28 L1 elements fall within the same loop, possibly insulating them from the influence of the enhancer. Additionally, numerous DNase I hypersensitive site (DHS) linkages, a marker for open chromatin, overlap specifically with L1-5219.

### Epigenetic analysis of L1Hs loci may help identify actively expressed unmappable or poorly mappable L1 elements

**Figure 8.**
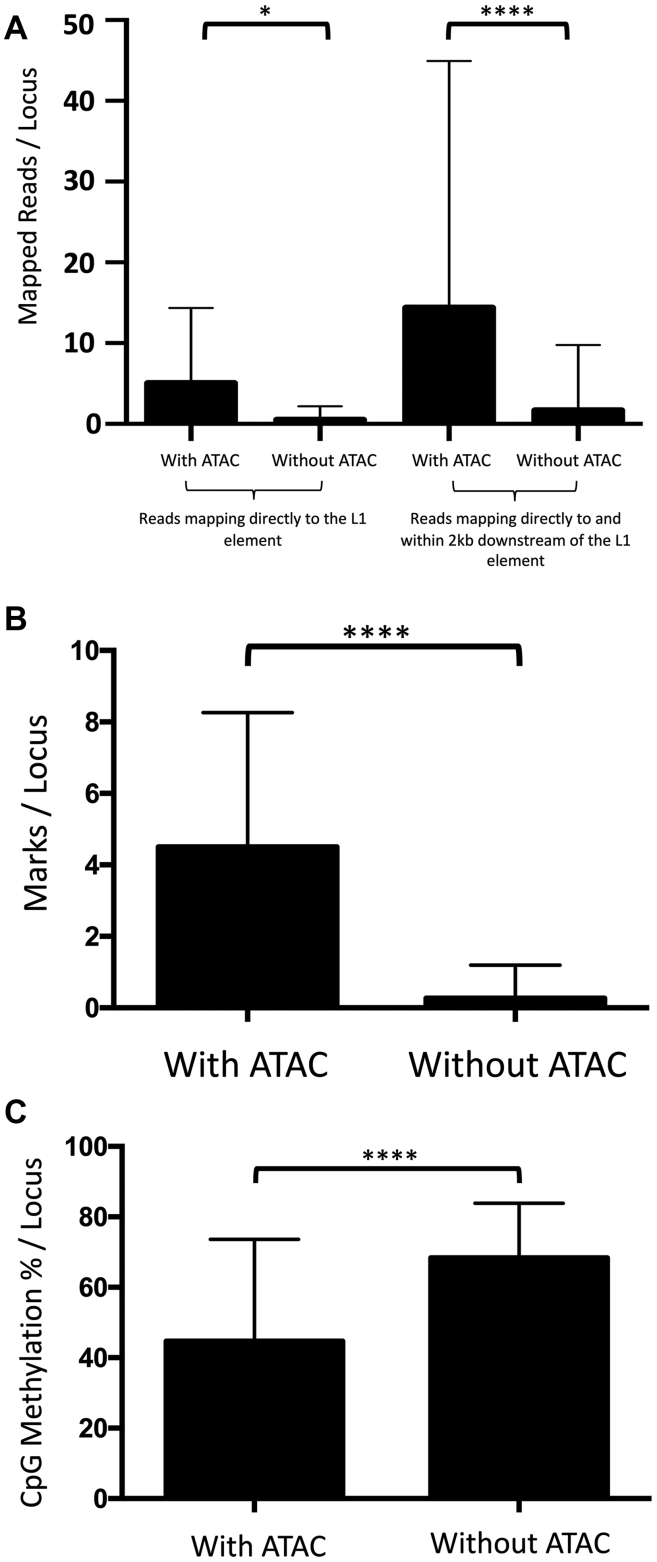
Epigenetic analysis identifies a subset of poorly mappable L1Hs loci that are likely expressed. (**A**) Reads mapping directly to the L1 element: RNA-Seq reads uniquely mapped to annotated L1Hs loci identified in MCF7 cells with and without an ATAC peak in MCF7 (AVG = 5.1 reads, *n* = 18 versus AVG = 0.5 reads, *n* = 228; *P< 0.05). Reads mapping directly to and within 2 kb downstream of the L1 element: Number of RNA-Seq mapped reads per locus with and without an ATAC peak when including 2 kb downstream of each locus is shown (AVG = 14.4 reads, *n* = 18 versus AVG = 1.7 reads, *n* = 228; ****, *P*< 0.0001). Student's *t*-test was used to analyze for significance. (**B**) Number of activating histone marks per L1Hs locus with and without an ATAC peak is shown (AVG = 4.5 marks, *n* = 18 versus AVG = 0.3 marks, *n* = 228; ****, *P*< 0.0001,). Student's *t*-test was used to analyze for significance. (**C**) Percentage of CpG methylation per L1Hs locus with and without an ATAC peak is shown (AVG = 44.7%, *n* = 18 versus AVG = 68.4%, *n* = 228; ****, *P*< 0.0001). Student's *t*-test was used to analyze for significance.

A major shortcoming associated with mapping RNA-Seq reads uniquely to L1 loci is that many repetitive elements are often indistinguishable from one another (22,32,111). Furthermore, their expression may not be clearly established when these loci are located in the same orientation as an expressed gene (22). These almost identical L1 loci tend to be the youngest L1 elements, L1Hs, that have the strongest potential for retrotransposition (21). In an effort to determine which of these unmappable L1 loci are most likely expressed by using the knowledge gained from our epigenetic analysis of mappable L1 loci, we analyzed the epigenetic features of 246 L1Hs loci present in the MCF7 genome that are also annotated in the human genome. We determined that the L1 loci associating with an ATAC peak (*n* = 18) have a significantly higher number of mapped reads that could map uniquely than loci without an ATAC peak (*n* = 228) (5.1 reads versus 0.5 reads, *P* < 0.05, Student's *t*-test) (Figure 8A). Additionally, as some L1 loci generate longer transcripts resulting from a read-through of the poly-A tail into a more mappable downstream region (112), we also found a significant difference in the number of mapped reads per locus between loci with and without an ATAC peak when including reads mapped within the 2 kb downstream of each L1 locus (14.4 reads versus 1.7 reads, *P* < 0.0001, Student's *t*-test). We also observed significant differences between loci with and without an ATAC peak when comparing both number of activating histone marks (4.5 marks versus 0.3 marks, *P* < 0.0001) and percent of methylated CpGs (44.7% versus 68.4%, *P* < 0.0001) (Figure 8B and C). Of the 18 L1Hs loci that overlap with an ATAC peak in MCF7 cells, three loci are expressed (above our threshold level), three loci have only 1–19 mapped reads and are therefore likely to be expressed at some level, and 12 loci have 0 mapped reads within the L1, although six of these have a few reads mapping downstream that may suggest some level of transcription with readthrough at the 3′ end. The six loci with no signs at all of transcription are consistent with the ‘transitional’ definition. By comparing the epigenetic context of individual loci, we are able to identify a subset of poorly mappable L1Hs L1 loci that are likely to be expressed in MCF7 cells based on favorable epigenetic conditions.

L1-0521 is an example of a poorly mappable L1 locus that is likely expressed in MCF7 cells based on its epigenetic features (Supplemental Figure S7A). This locus has only 3 mapped reads (11 when including 2 kb downstream) due to its sequence being similar to the L1 Hs consensus. Based on the few number of reads, this locus cannot be unambiguously identified as expressed during manual curation. However, we are able to identify this locus as likely expressed due to its positive association with an ATAC peak, high level of activating histone marks (10/12 marks), and low percentage of methylated CpGs (1.33%). Additionally, this locus is located within a CTCF loop and directly associates with 5 pol II loops (Supplemental Figure S7B). Although none of these pol II loops connect to an enhancer region containing a high level of activating histone marks, the L1 locus contains many activating histone marks (Supplemental Figure S7B). These results demonstrate that we are able to use the epigenetic context of L1Hs loci to predict which of these poorly mappable L1 elements are likely expressed.

### Endogenous L1 elements can be transiently transactivated to lead to an increase in exogenous Alu mobilization

**Figure 9.**
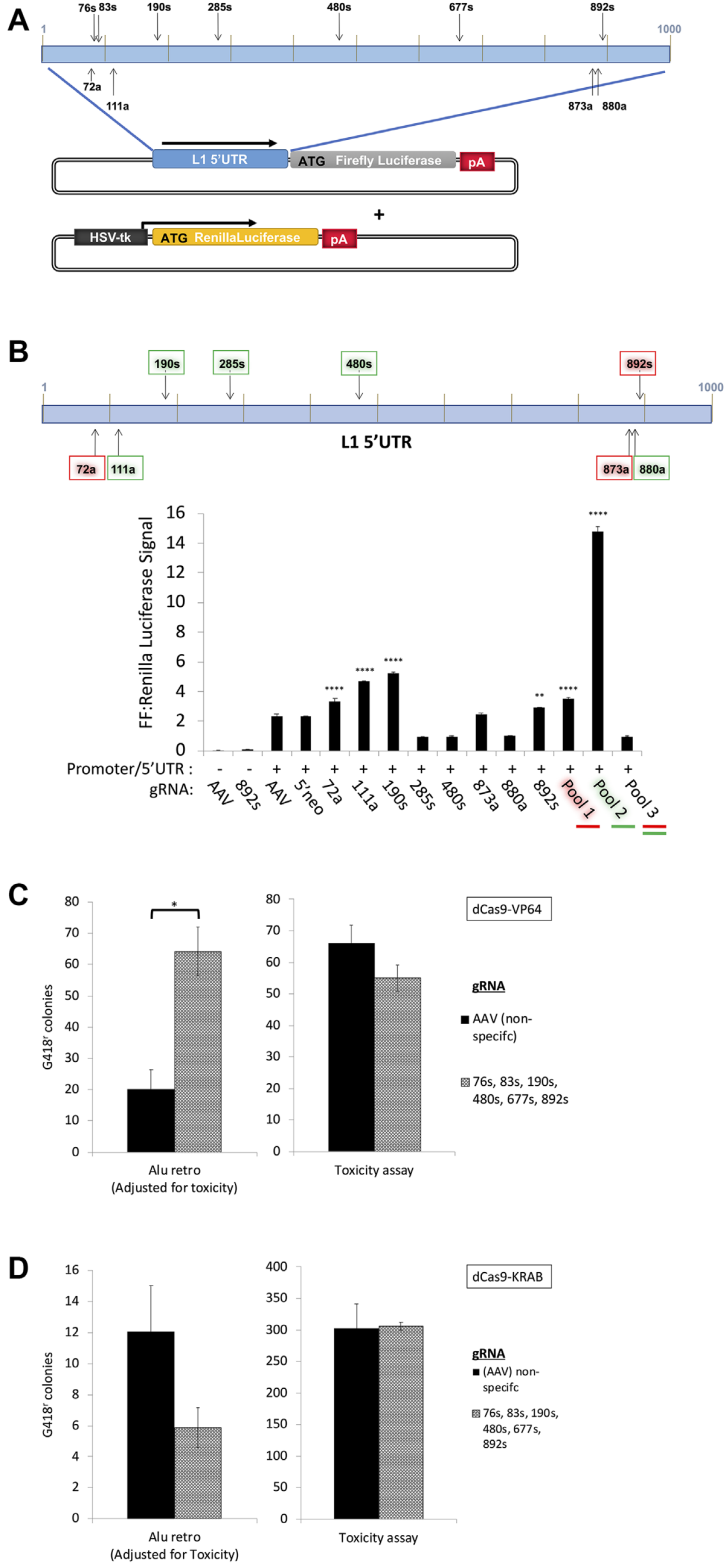
A CRISPR/Cas9a-based system targeting the L1 promoter alters expression of endogenous L1 loci in a biologically relevant manner. (**A**) A schematic of the plasmids used with CRISPR/Cas9a-based activating system targeting the L1 5′UTR. Experimental plasmid contains L1 5′UTR driving expression of the Firefly (FF) luciferase (83,97). Relative positions of sense (s) or antisense (a) gRNAs are indicated by vertical arrows in the enlarged schematic of the 5′UTR. A schematic of a control plasmid expressing Renilla luciferase under control of the HSV-tk promoter (pRL-TK) is shown. (**B**) Reporter gene approach shows that the human L1 5′ UTR can be activated by targeting gRNAs and CRISPR/Cas9a-based activating system. Schematic of the L1 5′ UTR shows the positions of the sense (s) or antisense (a) gRNA target sites (vertical arrows). gRNAs shown in red, green, or both were used in Pool 1, Pool 2 or Pool 3, respectively. HeLa cells were transfected with individual gRNAs or pools of gRNAs (marked as red and/or green). Each condition marked as + below the graph includes the plasmid with L1 Promoter/5′UTR-driven Firefly luciferase. The non-specific gRNAs targeting AAV and 5′neo (neither of which have homologous sequences in the human genome) were used as negative controls. Significance determined by comparison of Firefly:Renilla luciferase ratio to the negative control with the L1 Promoter plasmid and gRNAs targeting AAV (**P < 0.01; ****P < 0.0001, Student's *t*-test) (*n* = 2). (**C**) Results of Alu retrotransposition assay in the presence of CRISPR/Cas9a-based system (gRNAs targeting the 5′ UTR of endogenous L1 elements). Schematics for the Alu and toxicity assays are shown in Supplemental Figure S8B. HeLa cells were transfected with an Alu-insertion reporter plasmid (99), a plasmid expressing CAS9-VP64 domain, and a plasmid expressing gRNAs targeting either AAV or the L1 5′UTR. Significance was determined by Student's *t*-test (*P < 0.05) (*n* = 3). (**D**) Results of Alu retrotransposition assay in the presence of CRISPR/Cas9i-based system (gRNAs targeting the 5′ UTR of endogenous L1 elements). Schematics for the Alu and toxicity assays are shown in Supplemental Figure S8B. HeLa cells were transfected with Alu reporter plasmid, plasmid expressing CAS9-KRAB domain, and a plasmid expressing gRNAs targeting either AAV or the L1 5′UTR. A non-significant trend was determined by Student's *t*-test (*P*= 0.112) (*n* = 3).

In order to determine whether expression of L1 loci could be manipulated by altering chromatin state without changing DNA sequence, we utilized a CRISPR/Cas9 activator- (CRISPR/Cas9a) and CRISPR/Cas9 inhibitor- (CRISPR/Cas9i) based gene regulation system (113,114) with guide RNAs (gRNAs) targeting multiple locations on both strands within the L1 5′ UTR (Figure 9A). The CRISPR/Cas9a approach combines the guide RNAs, with CRISPR expression constructs fused to VP64 activating domain, while the CRISPR/Cas9i approach fuse the CRISPR with the KRAB repressor domain. Several guide RNAs complementary to the L1 5′UTR nucleotide positions 76, 83, 190, 285, 480, 677 and 892 in the sense and positions 72, 111, 366, 551, 765, 873 and 880 in the antisense orientation were tested alone, or as pools, for their ability to alter expression of the firefly luciferase reporter mRNA transcribed from the L1 5′ UTR in HeLa cells. While individual gRNAs were variably effective at specifically stimulating transcription of the firefly luciferase, pool 2 (containing five gRNAs targeting positions 111, 190, 285, 480 and 880, Figure 9B) was the most effective relative to the negative control transfection of a L1 5′ UTR reporter plasmid and gRNA targeting AAV (*P* < 0.01, *P* < 0.0001, Student's *t*-test). When replacing the Cas9-VP64 fusion with a Cas9-KRAB fusion in this system (CRISPR/Cas9i), we see a significant reduction in the L1 5′ UTR-driven reporter-gene expression when co-transfecting a pool of gRNAs as opposed to a negative control gRNA targeting AAV (*P* < 0.001, Student's *t*-test) (Supplemental FigureS 8A).

As a first approach to determine the influence of CRISPR/Cas9a on stimulation of expression of endogenous L1 elements, we used a biologically relevant Alu retrotransposition assay to measure mobilization of an exogenous Alu element by endogenously expressed L1s as previously described (21,115,116) (Supplemental Figure S8B). Alu elements are completely dependent on L1 ORF2p expression for retrotransposition (99). G418-resistant colonies generated in the absence of transfected L1 are produced by Alu retrotransposition driven by endogenously expressed L1 elements. In parallel, any toxicity from the treatment was measured by replacing the Alu reporter plasmid with a constitutive G418 resistance (neo^R^) selectable marker. Co-transfection of the Alu reporter plasmid, Cas9-VP64 plasmid, and a pool of gRNAs targeting the L1 5′UTR resulted in 3.2 times as many colonies as were observed in the presence of control gRNA targeting AAV (*P* = 0.0359, Student's *t*-test) (Figure 9C). In contrast, when a Cas9-KRAB fusion plasmid was used in this system, we observed a non-significant decrease in the number of G418-resistant colonies (*P* = 0.112) (Figure 9D) and in neither experiment did we detected any increased cellular toxicity (Figure 9C,D).

### Only a subset of endogenous L1 loci can be transactivated by the CRISPR/Cas9a approach

In order to identify which endogenous L1 loci are transactivated by the CRISPR/Cas9a system and the gRNAs targeting L1 sequences using RNA-Seq, we transfected HeLa cells with the CRISPR/Cas9a-expression plasmid along with the pool 2 plasmids expressing gRNAs shown in Figure 9B. As a control, transfection with an empty gRNA plasmid was performed in parallel. Paired-end, stranded RNA-Seq was performed using cytoplasmic RNA isolated from each of these transfected cell lines. Alignment of RNA-Seq reads, manual curation, and quantitation of reads mapped to individual full-length L1 loci was carried out as previously described (22,88). Transfecting HeLa cells with the pool 2 of gRNAs resulted in an approximately 1.5X increase in reads corresponding to expressed L1 loci, from 138.9 reads per million detected in the control to 198.5 reads per million upon activation.

**Figure 10.**
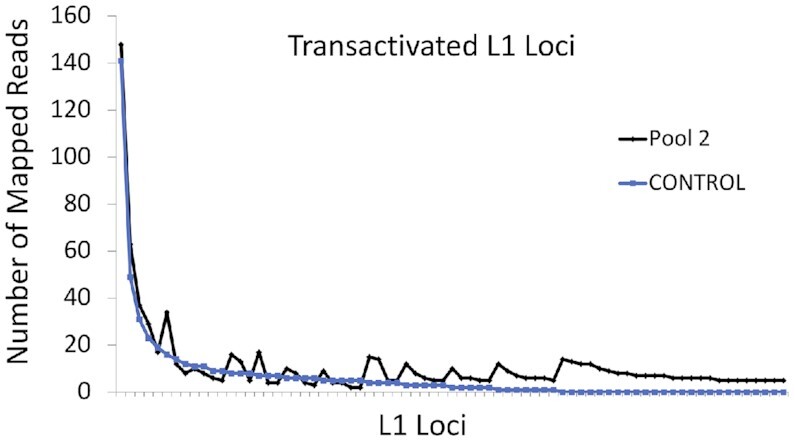
A CRISPR/Cas9a-based regulatory system activates endogenous unexpressed L1 elements. HeLa cells were transfected with a plasmid expressing CAS9-VP64 domain as well as plasmid expressing either pool 2 gRNAs (Figure 9A) (Pool 2) or empty gRNA control (CONTROL). PE RNA-Seq was performed and all L1 loci with 5+ mapped reads identified by RNA-Seq pipeline and manually validated as expressed in either the pool 2 (black) or control (blue) samples are displayed in the order from high to low number of reads as determined for Pool 2. HeLa cells transfected with pool 2 gRNAs had 65 loci with 5+ mapped reads and HeLa cells transfected with empty control gRNAs had 27 loci with 5 + mapped reads. Detailed information regarding these loci can be found on page 2 of the Additional File. The total number of mapped reads from RNA-Seq BAM files in the pool 2 and control HeLa cells is 17,927,082 and 16,581,681, respectively.

To determine which endogenous L1 loci were stimulated under these experimental conditions, we manually curated the L1 loci with 5 + mapped reads in both the CRISPR/Cas9a-treated cells and the empty gRNA plasmid control cells according to their individual expression level. All L1 loci with expression levels identified as 5+ reads in either the CRISPR/Cas9a-treated or control samples that pass manual curation are used for this comparison. We reported the expression level for each of these loci in both the activated and control cells (Figure 10, the loci expressed in the control cells (blue) versus the loci expressed in the CRISPR/Cas9a-treated cells (black; Additional File, page 2). The L1 loci with the highest levels of mRNA expression in the control sample exhibit similarly high levels of expression in the CRISPR/Cas9a- treated sample. Twenty-five L1 loci were expressed in the CRISPR/Cas9a-treated samples that had no expression (0 mapped reads) in the control. In contrast, no loci were found to be expressed in the control that had no expression in the CRISPR-Cas9a cells. These data demonstrate that the better expressed L1 loci are less likely to be stimulated to higher levels of expression than unexpressed loci. The vast majority of the nearly 5,000 full-length loci are unexpressed in both samples (0 mapped reads, *n* = 3638), demonstrating that only some L1 loci that may be somehow marked for expression can be stimulated through the recruitment of transcriptional activators.

We wondered whether any of the epigenetic marks studied here may help to explain which L1 loci are most susceptible to stimulation in our CRISPR/Cas9a experiments (Supplemental Figure S9A–C). We analyzed L1 loci shown in Figure 10 and categorized them into two groups: L1 loci that are activated from 0 reads to 5+ reads (*n* = 25) and loci that are unexpressed (0 mapped reads) in both the CRISPR/Cas9a-VP64-treated cells and control cells (*n* = 3638). We found a significant enrichment for ATAC peaks for the group of L1 loci that were subject to stimulation (36% versus 12.81%, *P* = 0.0017, Chi square analysis) (Supplemental Figure S9A). Additionally, we found a significant difference in the level of activating histone marks (1.12 marks versus 0.15 marks, *P* < 0.0001, Student's *t*-test) (Supplemental Figure S9B), but not methylation of CpGs (36.28% versus 46.01%, *P* = 0.087, Student's *t*-test) (Supplemental Figure S9C) between these two groups. Thus, the L1 loci we categorized as transitional (open chromatin near the promoter with no L1 mRNA expression) that associate with activating histone marks respond to transcriptional stimulation.

## DISCUSSION

Previous studies have determined that each cell type expresses only a small and fairly unique subset of genomic L1 loci (21,64,116). A strict, organ-specific expression of L1 loci is also observed *in vivo* (33). The number of expressed L1 loci is typically <1% of full-length L1 loci in most cell lines (22). Even in a cell line like MCF7 that supports some of the highest levels of L1 mRNA expression, only 3% of L1 loci express moderately (Figure 1B). Based on the finding that methylation can suppress L1 promoter function (38,39), a large number of previous studies have often assumed that global hypomethylation and histone activating marks at L1 promoters correlate with L1 expression (36,38,48,64,117–119). Our findings demonstrate that the sensitivity of those studies is often insufficient to correlate epigenetic marks with L1 expression because they were investigating the state of the small group of expressed L1 elements (∼50–100) pooled together with a large set of thousands of unexpressed L1 loci. For instance, we find that both the percent of global CpG methylation (Supplemental Figure S4B) and percent of CpG methylation per L1 locus (Supplemental Figure S4C) that are significantly higher in MCF7 than in HeLa and HEK293 cells do not correlate with the highest level of L1 mRNA expression detected in MCF7 cells (Figure 1A). However, significant hypomethylation of L1 promoters is detected when only expressed L1 loci are considered in our study (Figure 4). This finding highlights that global analysis of epigenetic state of L1 promoters does not sensitively reflect L1 mRNA expression. Thus, there is still a relatively poor understanding of what epigenetic features are characteristic of expressed L1 loci and studies focusing on analysis of expressed and unexpressed L1 loci as separate groups are needed to understand how L1 expression may be regulated during development, differentiation, and transformation.

We have focused our studies on individual highly expressed L1 loci in MCF7 cells. We find that there is a strong correlation between L1 locus expression status and their promoter's association with epigenetic signals that are typically found at the promoters of expressed cellular genes. Specifically, the presence of an ATAC peak over the L1 promoter, histone activating marks around the L1 promoter region, and promoter hypomethylation all show significant enrichment in L1 loci expressed in MCF7 cells compared to unexpressed L1 loci (Figures 2–4). In HeLa and HEK293 cells, we also find enrichment for ATAC peaks (Figure 2) and activating histone marks (Supplemental Figure S3A-C) at promoters of the expressed L1 loci. In contrast to the findings in MCF7 cells, we were unable to detect hypomethylation at L1 loci in expressed HeLa and HEK293 cells (Supplemental Figure S4A), probably reflecting that these cells have relatively few and poorly expressed loci (Figure 1). When comparing the epigenetic status of loci expressed and unexpressed in MCF7 cells within HeLa and HEK293 cells, we find that loci expressed in MCF7 retain their associations with activating histone marks (Supplemental Figure S5A,B) and ATAC peaks (Supplemental Figure S5D) as a group in both HeLa and HEK293. This is likely due to the fact that 41 of the 162 L1 loci are expressed in HeLa cells and 53 of the 162 L1 loci are ‘transitional’ (Figure 5). As expected, within this group of 162 L1 loci expressed in MCF7 cells, there are fewer L1 loci that have activating histone marks in HeLa cells (Figure 3A and Supplemental Figure S5A), a likely reflection of the L1 loci that are not expressed in HeLa cells. In contrast to ATAC and activating histone marks, methylation status of the 162 L1 loci in HeLa and HEK 293 cells does not distinguish them from the unexpressed L1 loci in the manner it does in MCF7 cells (Supplemental Figure S5C and Figure 4). These findings indicate a role for open chromatin, activating histone marks, and CpG methylation in contributing to cell-line specific expression of individual L1 loci.

ATAC, Chip-Seq, and bisulfite sequencing analyses of expressed and unexpressed L1 loci determined that no single epigenetic factor is sufficient to clearly predict expressed L1 loci (Figures 2–4). However, these approaches have identified a subset of L1 loci that are not expressed, but have an ATAC peak (Figure 2), often contain histone activating marks (Figure 3B), and are relatively hypomethylated (Figure 4). We refer to these as ‘transitional’ L1 loci, which we consider to have some of the necessary features for expression, but still are missing at least one of the necessary factors to be expressed. This consideration is based on the finding that stimulation of endogenous L1 mRNA expression using the CRISPR/Cas9a system results in the upregulation of the poorly expressed and ‘transitional’ loci (Supplemental Figure S9), suggesting that these loci are susceptible to reaching the critical mass of activating factors to launch their expression.

In addition to identifying differences in the local epigenetic factors at the promoters of expressed and unexpressed L1 loci, we discovered that promoters of expressed L1 loci appear to associate with an active transcription enhancer. This is best seen using the three-dimensional ChIA-PET data that demonstrate an RNA polymerase II-driven association between the promoters of expressed L1 loci and an enhancer with strong histone activating marks (Table 1, Figure 6, Supplemental Figure S6). This seems to be one of the major factors that distinguishes the expressed loci from the ‘transitional’ L1 loci, although there are exceptions (Table 1). It seems likely that the three-dimensional L1 promoter association with an enhancer is also influenced by the formation of specific CTCF-mediated interactions. This is supported by the finding that L1 elements near a highly expressed L1 element, like those nearby L1-5219, are not expressed because they are not localized within the CTCF/cohesin loop(s) that also contains RNA Pol II-mediated loops with strong enhancers (Figure 7B). This is further supported by the CRISPR/Cas9a experiments demonstrating that a targeted recruitment of pol II to ‘transitional’ L1 loci results in their expression (Figure 10, Supplemental Figure S9).

Our results highlight the benefit of analyzing individual expressed L1 loci as a group to demonstrate the complexity and variability of factors involved in expression of different L1 loci. In addition, they highlight the power of epigenetic measures to guide and confirm expression analysis of individual L1 loci. For instance, ATAC analysis alone limits the number of L1 loci to be considered for potential expression by ∼10-fold (from 5,000 to ∼500). Combining ATAC or activating histone marks with RNA polymerase II association with an enhancer sequence in MCF7 cells provides even more power (Table 1). Building on our findings, epigenetic analysis may help identify which L1Hs loci are expressed but cannot be unambiguously detected with RNA-Seq due to poor mappability. Our data demonstrate that the 18 (out of 246) L1Hs loci present in MCF7 cells that have an ATAC peak contain a higher number of activating histone marks and lower levels of CpG methylation compared to L1Hs loci without an ATAC peak (Figure 8, Supplemental Figure S7). As only three of these 18 loci are expressed above our threshold in MCF7 cells (Additional File, page 3), we predict that some, but not all, of the other 15 loci are likely expressed at some level due to their favorable epigenetics conditions below threshold levels of expression. Consistent with ATAC performance on mappable L1 loci, it provides a greater than 10-fold reduction in the number of L1Hs loci to be considered for expression. As L1Hs loci are younger elements that are more likely to be highly expressed and retrotranspositionally-competent (21,120,121), being able to identify which of these loci are expressed may have important prognostic implications regarding the mutagenic effects of L1 expression (122,123).

Our analysis of epigenetic marks at individual expressed and unexpressed L1 loci establishes a roadmap for manipulation of L1 loci expression either through a CRISPR/Cas9a system or knockout of specific sequences, as well as for studies of normal changes occurring at L1 promoters in different biological settings. We would predict that the ‘transitional’ loci in a tissue would be most prone to activation during development or through the oncogenic transformation. In fact, combined with cell type specific L1 mRNA expression (5), our findings demonstrate that any biological process associated with perturbations of epigenetic and/or three-dimensional genomic landscape, such as cancer, aging, environmental exposures, and even normal developmental processes, would be expected to involve epigenetic features that are identified in this study to alter L1 expression patterns. Cancer cells, for instance, typically have much higher levels of L1 expression than do normal tissues (27,124–126). One previously used explanation was that hypomethylation of L1 promoters that may occur in the tumor could have a broad impact on L1 loci expression. However, as expression of individual L1 elements is highly dependent on cellular context, as seen in various mouse tissues (33) and human cell lines (22,32) (Figure 1), our data demonstrate that a wide array of cancer-specific changes, including sequence rearrangements repositioning CTCF binding sites, individual interactions between L1 elements and their enhancers, or changes in RNA Pol II recruitment through transcriptional factors, influence expression of individual L1 loci.

Perhaps the most general finding in our studies is that the L1 element by itself is probably insufficient for significant expression without help from its local genomic environment. Some level of open chromatin, allowing detection of an ATAC signal at the promoter, as well as activating histone marks is clearly necessary, but not sufficient for L1 expression as demonstrated by the presence of ‘transitional’ L1 loci. A dominant feature of expressed L1 loci also seems to be the presence of a three-dimensional interaction between the L1 promoter and a strong enhancer. Unlike a typical gene whose entire genomic environment evolves as a unit or a virus that brings its own enhancer, a new L1 insert is placed in a completely unique sequence and epigenetic environment. The distinct features of individual environments will immediately have specific, but likely different, influences on expression for newly inserted L1 loci. Thus, following integration, each L1 locus will evolve as a completely independent gene. This is supported by the results of our CRISPRa stimulation of L1 expression (Figures 9 and 10). The unique feature of this approach is that essentially the same promoter is stimulated at different locations with different results depending on those environments. This is a unique situation compared to genes. It is also an important technical observation in that it shows investigators a working approach to stimulate endogenous L1 expression to look for the influence of changes in L1 expression in a manner that is much more physiological than transfecting in an exogenous L1. Based on these findings, it might be expected that a new L1 insert that landed in the genomic environment that is permissive of its expression to high levels in many cell types (i.e. similar to a housekeeping gene) would experience high negative evolutionary pressure and be more likely to be eliminated by selection. This is consistent with the finding that full-length L1 loci are deleterious and have been under purifying selection (127–129). This may well explain the strong cell-type and tissue-specific expression patterns from individual L1 loci (22,32,130) because expressing an L1 locus ubiquitously would be more deleterious.

Overall, in this study we identify key local (chromatin state, methylation status, activating histone marks at individual L1 promoters) and long-distance (PolII interactions, CTCF loops, activating histone marks at relevant enhancers) epigenetic features that are different between expressed and unexpressed L1 loci. We demonstrate their relative power in distinguishing expressed from unexpressed L1 loci. We demonstrate that our discoveries can be used for predicting expression of poorly mapped, but the most disease relevant, L1Hs loci, expression of which cannot be unambiguously determined by RNA-Seq alone. Based on their epigenetic and expression status we identify a new category of L1 loci we term ‘transitional’ because they have several epigenetic features unique to expressed L1 loci even though they are not expressed. Using L1-specific CRISPR/Cas activation and inhibition approaches, we demonstrate as a proof-of-principle that expression of individual endogenous L1 loci can be manipulated, but the three classes of L1 loci (expressed, unexpressed and transitional) respond differently to this manipulation with transitional L1 loci being the most responsive to the induced activation. Finally, we demonstrate that activation of endogenous L1 mRNA expression is biologically relevant and measurable because we observe a significant increase in Alu retrotransposition in response to L1 activation.

## DATA AVAILABILITY

All data will be made available to the public upon publication. The manuscript's metadata is available for viewing via SRA at https://dataview.ncbi.nlm.nih.gov/object/PRJNA724674?reviewer = hs0mv88e2vgn8br01t2pbm139r.

Publicly-available data can be accessed at the following accession numbers:

- RNA-Seq:
  - MCF7: ENCFF000HSC and ENCFF000HSK (ENCODE)
  - HeLa: Data can be found at link provided in AVAILABILITY section (SRA)
  - HEK293: SRR1275413 (GEO)
- ATAC-Seq: Data for MCF7, HeLa and HEK293 can be found at link provided in AVAILABILITY section (SRA)
- WGBS: Data for MCF7, HeLa and HEK293 can be found at link provided in AVAILABILITY section (SRA)
- Histone Marks CHIP-Seq (ENCODE):
- MCF7
- H3K27Ac
- ENCSR752UOD
- H3K9Ac
- ENCSR056UBA
- H3K4Me3
- ENCSR985MIB
- H3K4Me2
- ENCSR875KOJ
- H3K4Me1
- ENCSR493NBY
- H4K20Me1
- ENCSR639RHG
- HeLa
- H3K27Ac
- ENCSR000AOC
- H3K9Ac
- ENCSR000AOH
- H3K4Me3
- ENCSR340WQU
- H3K4Me2
- ENCSR000AOE
- H3K4Me1
- ENCSR000APW
- H4K20Me1
- ENCSR000AOI
- HEK293
- H3K27Ac
- ENCSR000FCH
- H3K4Me3
- ENCSR000DTU
- H3K4Me1
- ENCSR000FCG

Additional information regarding data used in this manuscript can be found in the Materials and Methods section.

## Supplementary Material

gkac013_Supplemental_Files
