# Supplementary material for: Analysis of epigenetic features characteristic of L1 loci expressed in human cells
Source: Nucleic Acids Res. 2022 Jan 31;50(4):1888–907. doi: 10.1093/nar/gkac013 (PMC8887483; doi:10.1093/nar/gkac013)
Supplement: gkac013_Supplemental_Files [file gkac013_supplemental_files.zip › Additional Files 1-2 legends.docx]

**Additional File 1. Detailed information on specific L1 loci in MCF7, HeLa, and/or HEK293 cells.** Page 1 contains information regarding the 162 loci expressed in MCF7 cells. Page 2 contains information regarding the 73 L1 loci plotted in the CRISPRa experiment depicted in Figure 10. Page 3 contains information regarding the 18 L1Hs loci with ATAC peaks in MCF7 cells. Page 4 contains information regarding the 38 L1 elements described in Rodriguez-Martin et al. [100].

**Additional File 2. Sequences of gRNAs used to target the L1 promoter.** Plasmids containing these gRNA sequences were used in the CRISPRa (Figure 9B,C, Figure 10) and CRISPRi (Figure 9D, Supplemental Figure 8A) experiments. The plasmid containing the gRNA targeting AAV (gRNA_AAVS1-T2) was purchased from Addgene [97].
