# Supplementary material for: Analysis of epigenetic features characteristic of L1 loci expressed in human cells
Source: Nucleic Acids Res. 2022 Jan 31;50(4):1888–907. doi: 10.1093/nar/gkac013 (PMC8887483; doi:10.1093/nar/gkac013)
Supplement: gkac013_Supplemental_Files [file gkac013_supplemental_files.zip › Freeman-et-al-Supplemental Figures.docx]

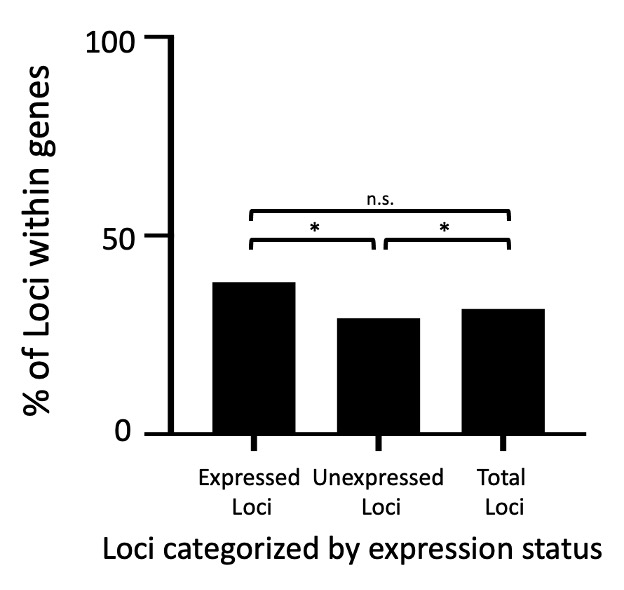


**Supplemental Figure 1. Percent of expressed, unexpressed, and total loci that are present within genes in MCF7 cells.** Percentage of loci located within genes is shown for expressed (38.3% of loci), unexpressed (29.2%), and total loci (31.5%) in MCF7 cells. Significance determined by Chi Square analysis with Yates’ correction (n.s., not significant; *, *p* < .05).


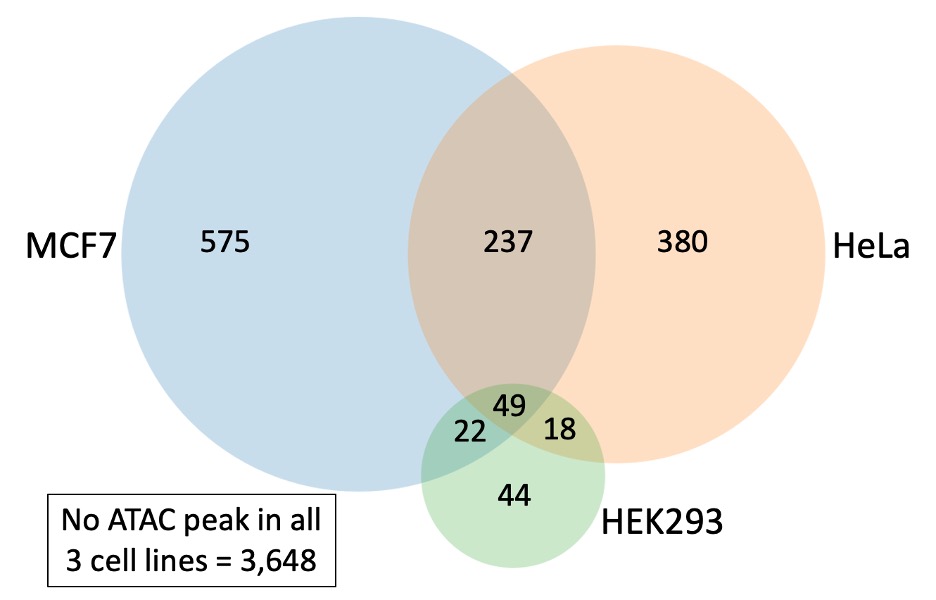


**Supplemental Figure 2. Shared and unique L1 loci with or without ATAC peaks in MCF7, HeLa, and HEK293 cells.** The Venn diagram depicts shared and unique L1 loci based on their positive ATAC status among MCF7 (blue), HeLa (orange), and HEK293 (green) cell lines out of 4,973 loci . The majority of loci (3,648 loci) do not have an ATAC peak in any of the three cell lines analyzed.


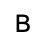

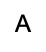

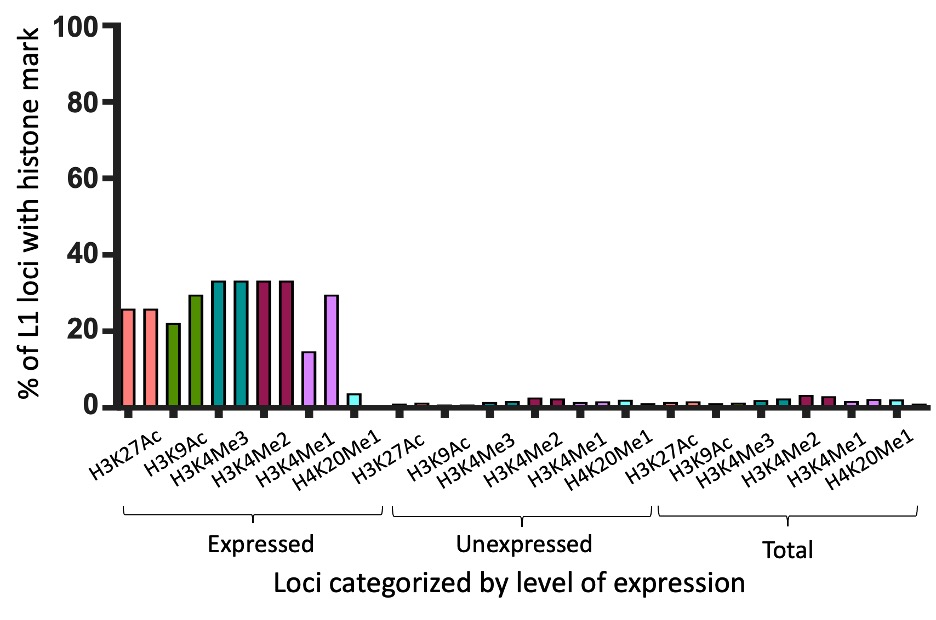


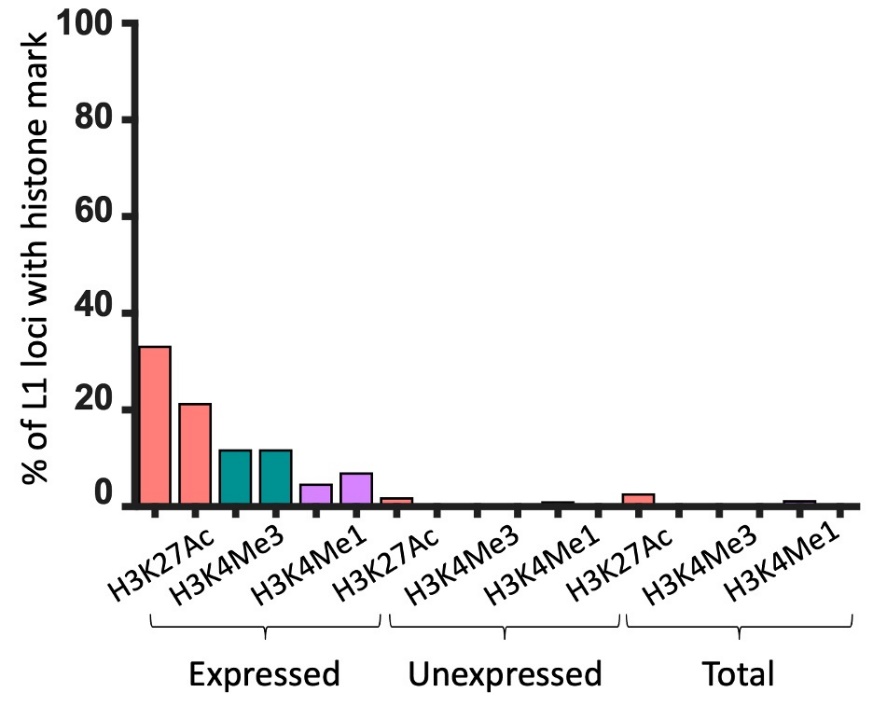


**Supplemental Figure 3. Characterization of activating histone marks at promoters of expressed, unexpressed, and total L1 loci in HeLa and HEK293 cells. (A)** Analysis of six activating histone marks at promoters of expressed (n = 27), unexpressed (n = 4,508), and total loci (n = 4,973) in HeLa cells (*p* < .0001). For five of the histone marks, H3K27Ac, H3K9Ac, H3K4Me3, H3K4Me2, and H3K4Me1, the percent of histone marks present at expressed loci is significantly higher compared to unexpressed and total loci as determined by Chi Square analysis with Yates’ correction (*p* < .0001). **(B)** Analysis of three activating histone marks at promoters of expressed (n = 42), unexpressed (n = 3,655), and total loci (n = 4,973) in HEK293 cells. In HEK293 cells, data for only 3 activating histone marks, H3K27Ac, H3K4Me3, and H3K4Me1, were available for analysis. For all three histone marks, the percent of histone marks present at expressed loci is significantly higher compared to unexpressed and total loci as determined by Chi Square analysis with Yates’ correction (*p* < .0001).


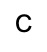

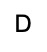

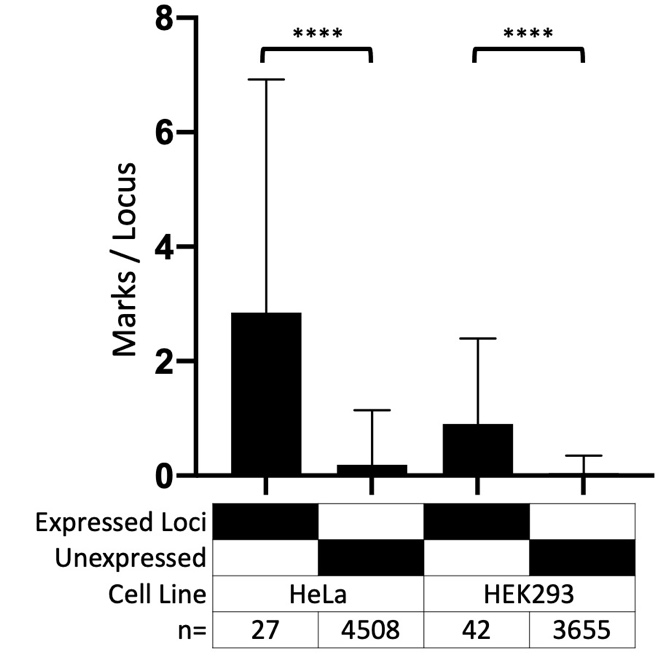


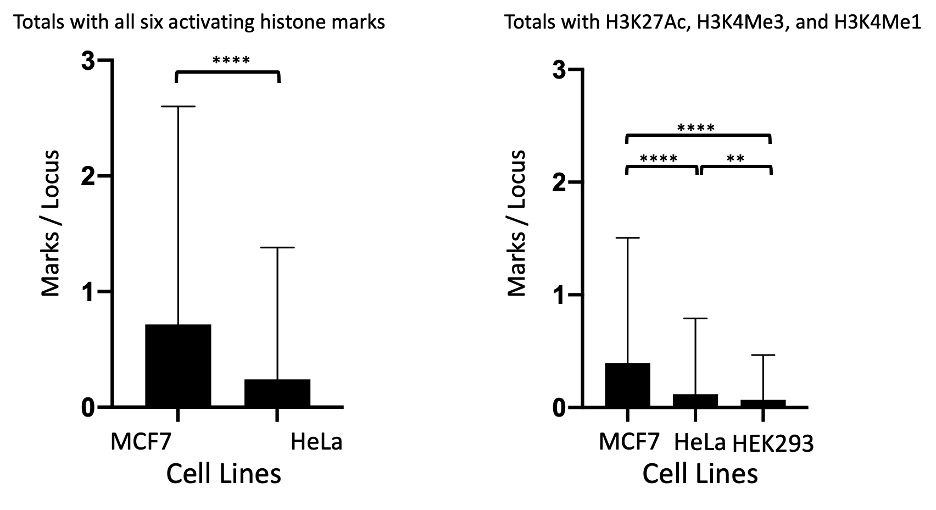


**Supplemental Figure 3. Characterization of activating histone marks at promoters of expressed, unexpressed, and total L1 loci in HeLa and HEK293 cells.** **(C)** Analysis of activating histone marks per locus of expressed and unexpressed loci in HeLa (2.85 marks vs. .19 marks, *p* < .0001) and HEK293 (.9 marks vs. .05 marks, *p* < .0001) cells. In HEK293 cells, data for only 3 activating histone marks, H3K27Ac, H3K4Me3, and H3K4Me1, were analyzed. The average number of marks is determined out of 12 (HeLa) or 6 (HEK293) to account for each of these marks being analyzed in two experiments. Significance difference was determined by student’s t-test (****, *p* < .0001). **(D)** Left: MCF7 cells show a higher average number of activating histone marks per locus (.72 marks) as compared to HeLa cells (.24 marks) when all L1 loci are considered for analysis. Average number of activating histone marks is calculated out of 12 to account for each mark being analyzed in two experiments. Right: In HEK293 cells, only 3 activating histone marks, H3K27Ac, H3K4Me3, and H3K4Me1, were available for this analysis. Using data only from these three marks, MCF7 cells show a higher average number of activating histone marks per locus (.39 marks) as compared to HeLa (.12 marks) and HEK293 (.07 marks) cells. All 4,973 loci were used in this analysis for each cell line. Average number of activating histone marks is calculated out of 6 to account for each mark being analyzed in two experiments. Significance between cell lines was determined by student’s t-test (**, *p <* .01; ****, *p* < .0001).


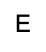


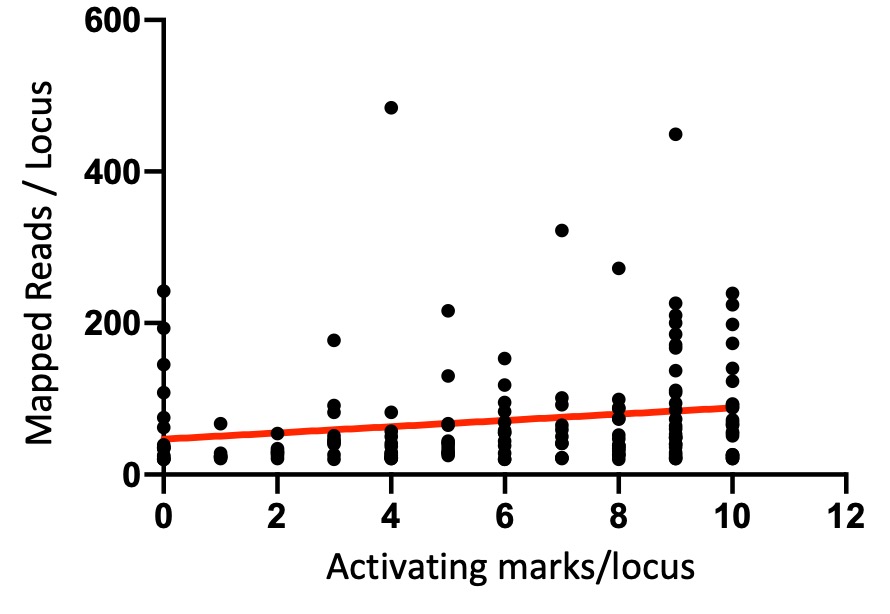


**Supplemental Figure 3. Characterization of activating histone marks at promoters of expressed, unexpressed, and total L1 loci in HeLa and HEK293 cells.** **(E)** A relationship between the number of mapped reads and the number of activating histone marks per L1 locus is examined for L1 loci expressed in MCF7 cells. Average number of activating histone marks is calculated out of 12 to account for each mark being analyzed in two experiments. A positive correlation is determined by Pearson correlation test (*, *p* = .017, R squared = .035).


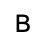

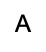

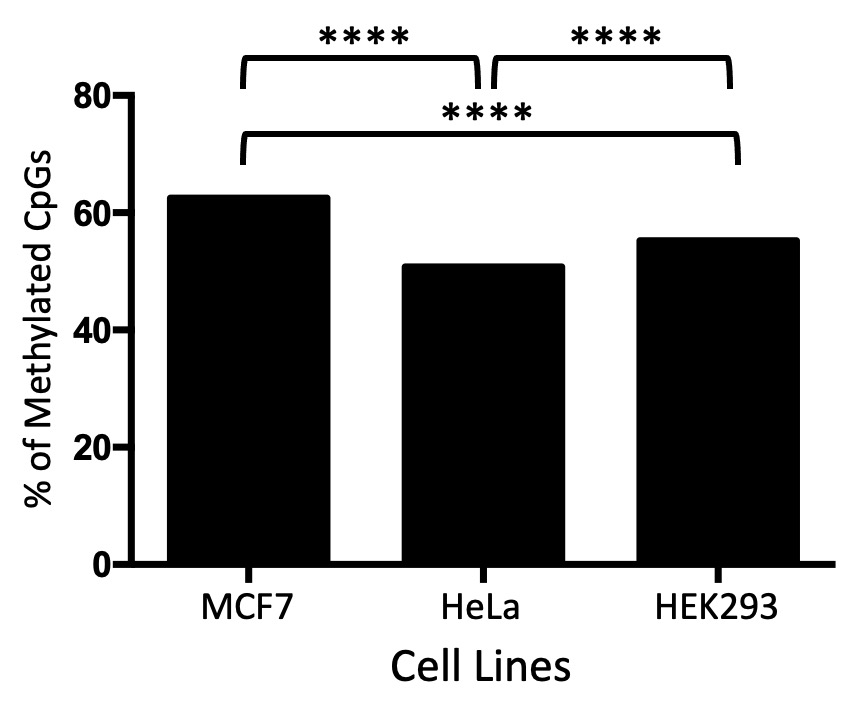

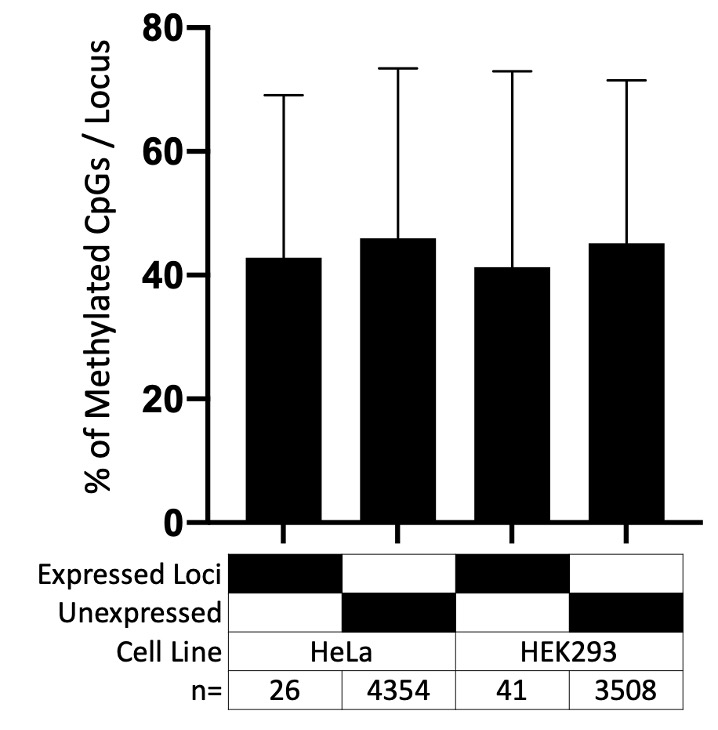


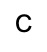


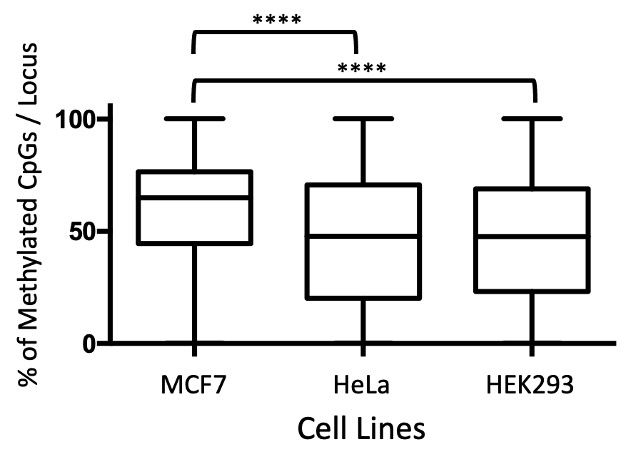


**Supplemental Figure 4. CpG methylation of expressed and unexpressed L1 loci in HeLa and HEK293 cells. (A)** Percent of CpG methylation is determined for each L1 locus expressed and unexpressed in HeLa cells (expressed, 42.85%, n = 26; unexpressed, 45.99%, n = 4354; *p* = .55) and HEK293 (expressed, 41.3%, n = 41; unexpressed, 45.14%, n = 3,508; *p* = .37). A total of 159 and 156 loci in HeLa and HEK293 cells, respectively, were unmapped following bisulfite sequencing and thus excluded from this analysis. Significance between expressed and unexpressed loci in each cell line was determined by student’s t-test. **(B)** Global analysis of methylation status of all CpGs in MCF7, HeLa, and HEK293 cells. The total number of CpGs in the genome of each cell line are: MCF7 = 100.3x10^6 CpGs, HeLa = 98.3x10^6, and HEK293 = 94.1x10^6. Significance was determined by Chi Square analysis with Yates’ correction (****, *p* < .0001). **(C)** Analysis of CpG methylation is performed as in A, but including all L1 loci in MCF7, HeLa, and HEK293 cells regardless of their expression status (n = 4,973). A total of 158, 159, and 156 loci in MCF7, HeLa, and HEK293 cells, respectively, were unmapped following bisulfite sequencing and thus excluded from this analysis. Significance determined by student’s t-test (****, *p* < .0001).


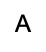

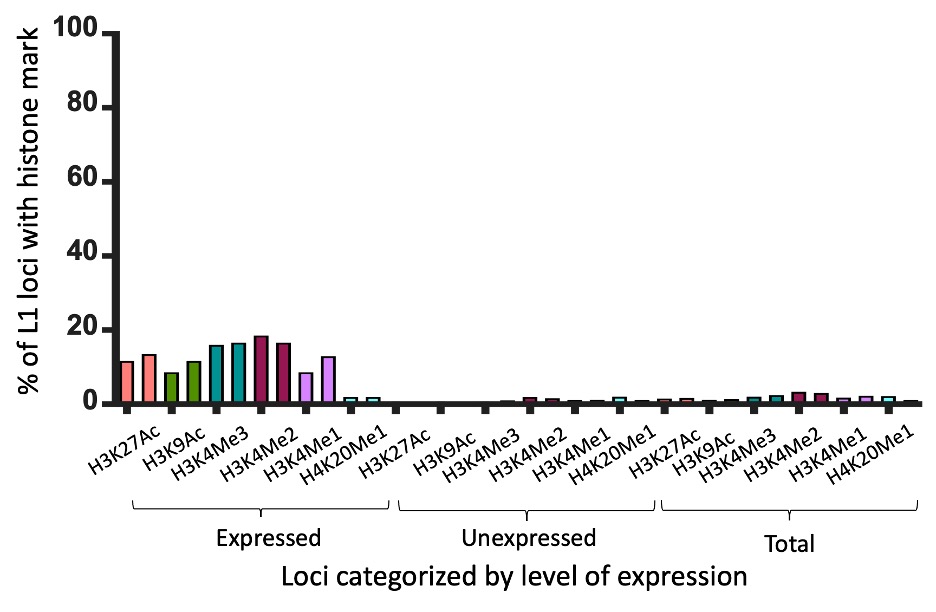


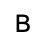


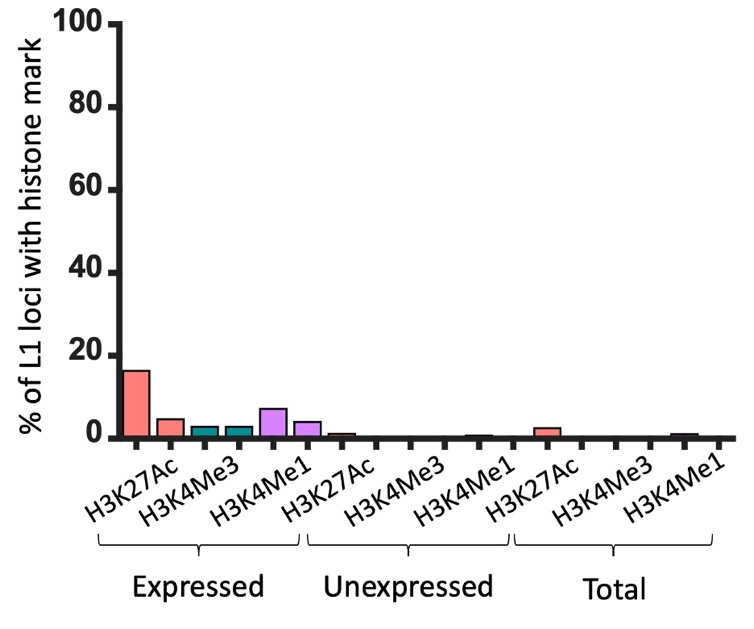


**Supplemental Figure 5. Epigenetic status of the 162 loci expressed in MCF7 cells within HeLa and HEK293 cells. (A-B)** Activating histone marks at promoters of L1 loci determined to be expressed (n = 162) and unexpressed (n = 3,028) in MCF7 cells, along with total loci (n = 4,973), are evaluated in HeLa cells **(A)** and HEK293 cells **(B)**. Each of the six activating histone marks was evaluated in HeLa cells. Data for only 3 activating histone marks was available in HEK293 cells. For five of the six histone marks in HeLa cells **(A)**, H3K27Ac, H3K9Ac, H3K4Me3, H3K4Me2, and H3K4Me1, the percent of histone marks present at expressed loci is significantly higher compared to unexpressed and total loci as determined by Chi Square analysis with Yates’ correction (*p* < .0001). For all three histone marks in HEK293 cells **(B)**, the percent of histone marks present at expressed loci is significantly higher compared to unexpressed and total loci as determined by Chi Square analysis with Yates’ correction (*p* < .0001).


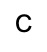


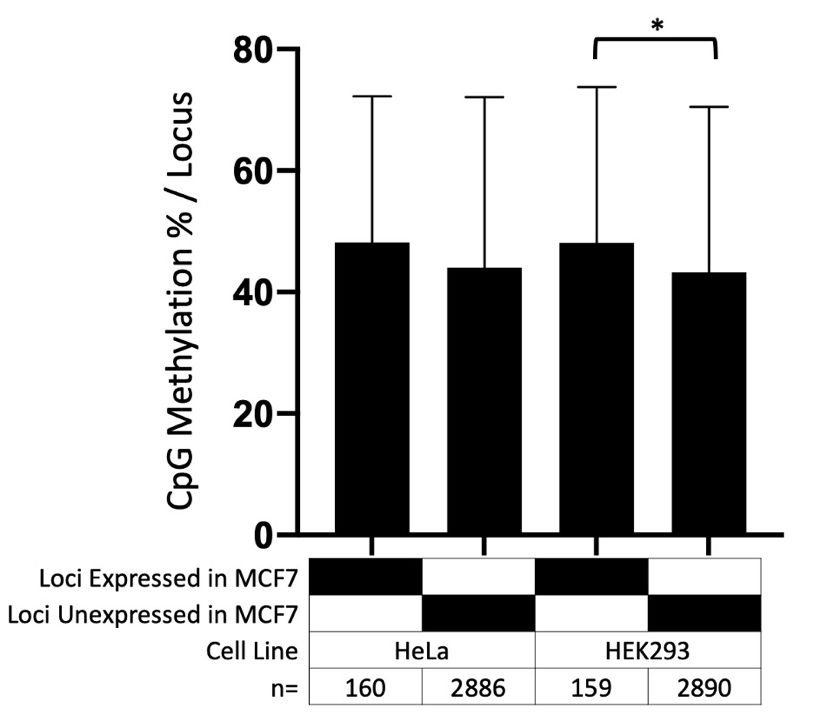


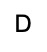

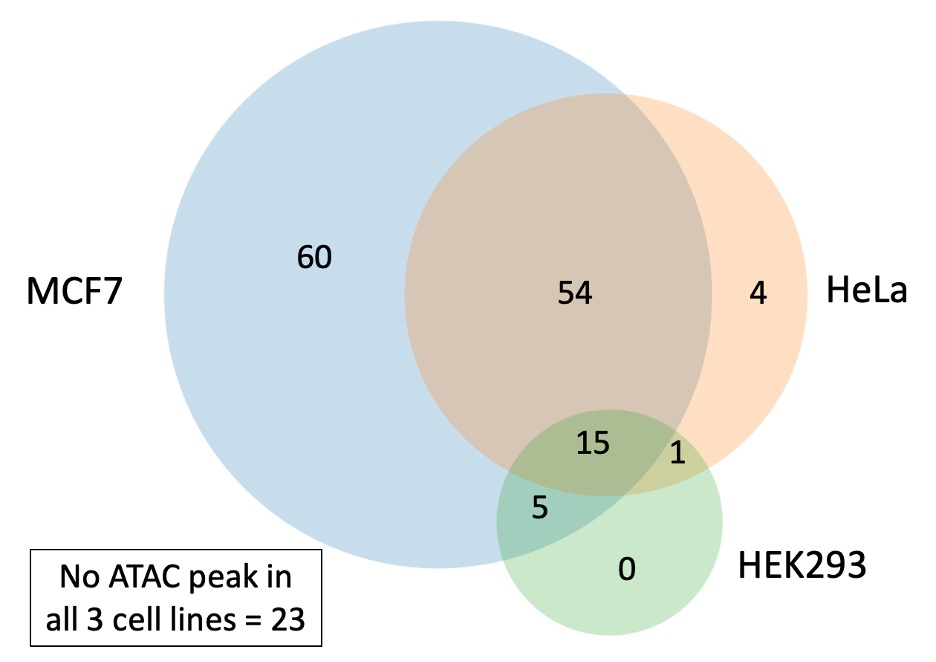


**Supplemental Figure 5. Epigenetic status of the 162 loci expressed in MCF7 cells within HeLa and HEK293 cells. (C)** Percent of CpG methylation at promoters of the 162 L1 loci expressed and 3,028 L1 loci unexpressed in MCF7 cells is analyzed in HeLa and HEK293 cells. In HeLa cells, two expressed loci and 142 unexpressed loci were unmapped following mapping of bisulfite sequencing reads and thus excluded from this analysis. In HEK293, three expressed loci and 138 unexpressed loci were unmapped following mapping of bisulfite sequencing reads and thus excluded from this analysis. **(D)** Venn diagram depicts the number of shared and unique L1 loci based on their positive ATAC status out of the 162 loci that are categorized as expressed in MCF7 cells in all 3 cell lines.


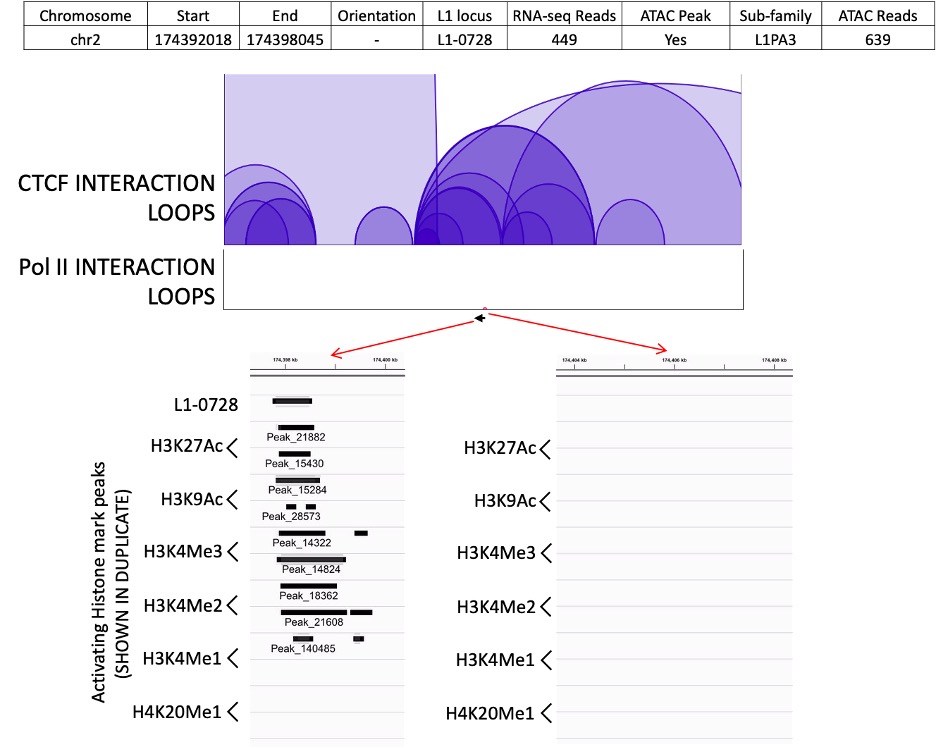


**Supplemental Figure 6A**

**Supplemental Figure 6. Long-distance interactions of individual L1-loci in MCF7 cells. (A-AA)** Diagrams of L1-0728 **(A)**, L1-3682 **(B)**, L1-1867 **(C)**, L1-2476 **(D)**, L1-3165 **(E)**, L1-0029 **(F)**, L1-3455 **(G)**, and L1-1685 **(H)**, and L1-3239 **(I)** represent nine of the ten highest expressed loci in MCF7 cells. Diagrams of L1-2855 **(J)**, L1-0225 **(K)**, L1-0986 **(L)**, L1-4910 **(M)**, L1-5151 **(N)**, L1-1469 **(O)**, L1-0482 **(P)**, L1-3525 **(Q)**, and L1-4180 **(R)** represent nine of the ten transitional loci (unexpressed loci overlapping with an ATAC peak) with the largest peaks produced by mapping of ATAC sequencing reads. Diagrams of L1-0518 **(S)**, L1-1501 **(T)**, L1-1960 **(U)**, L1-4228 **(V)**, L1-4249 **(W)**, L1-4821 **(X)**, L1-4938 **(Y)**, L1-5330 **(Z)**, and L1-5742 **(AA)** represent nine of ten random unexpressed loci in MCF7 cells. Random loci were selected to ensure representation from each L1PA1-8 subfamily. One transitional L1Hs locus, L1-1337, was excluded from this analysis due to poor mappability. Each diagram includes information regarding the locus location (chromosome, start site, end site, and orientation), RNA-seq reads, presence of an ATAC peak, L1 sub-family, and number of reads from ATAC sequencing. Loops indicate CTCF binding sites (purple, shaded) within 500 kb of the L1 start site (black arrow). RNA polymerase II (Pol II) loops (red) are only shown if the pol II binding site overlaps within 500bp of the L1 start site. The CTCF loops are shown at a scale of 500kb upstream and downstream of the L1 start site. Red arrows indicate magnification of the indicated genomic region, which contains information regarding the presence of the activating histone marks from previously described CHIP-seq data (Figure 3A). Results of analysis of all histone marks are shown from two experiments for both the L1 promoter and putative enhancer ends of the RNA polymerase II loops.


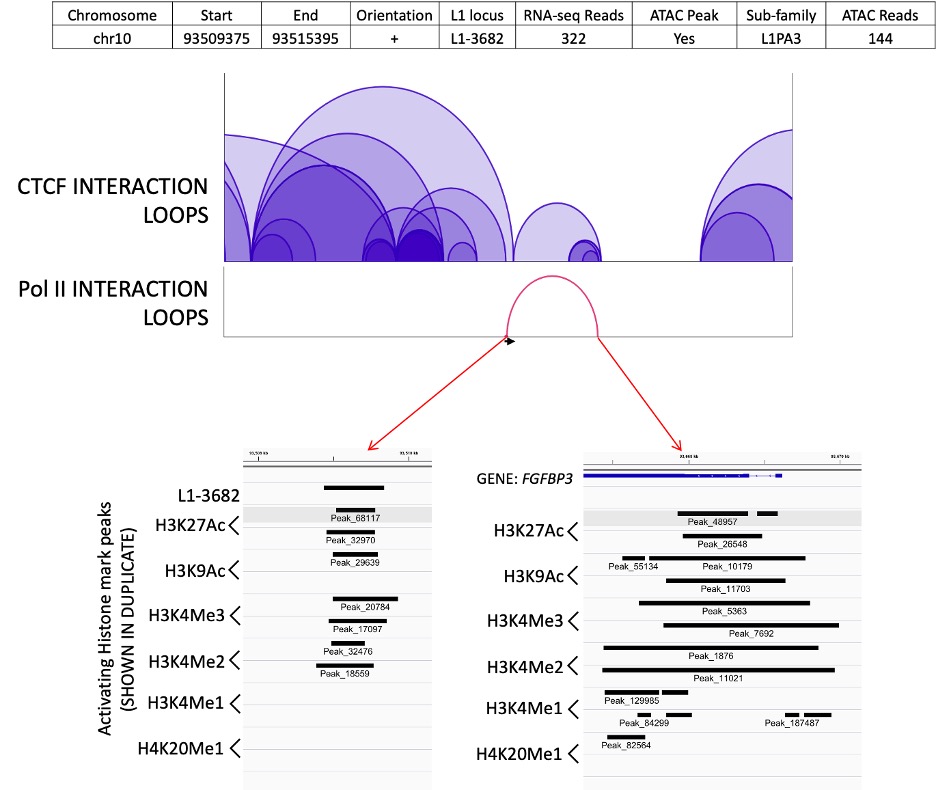


**Supplemental Figure 6B**

**Supplemental Figure 6. Long-distance interactions of individual L1-loci in MCF7 cells. (A-AA)** Diagrams of L1-0728 **(A)**, L1-3682 **(B)**, L1-1867 **(C)**, L1-2476 **(D)**, L1-3165 **(E)**, L1-0029 **(F)**, L1-3455 **(G)**, and L1-1685 **(H)**, and L1-3239 **(I)** represent nine of the ten highest expressed loci in MCF7 cells. Diagrams of L1-2855 **(J)**, L1-0225 **(K)**, L1-0986 **(L)**, L1-4910 **(M)**, L1-5151 **(N)**, L1-1469 **(O)**, L1-0482 **(P)**, L1-3525 **(Q)**, and L1-4180 **(R)** represent nine of the ten transitional loci (unexpressed loci overlapping with an ATAC peak) with the largest peaks produced by mapping of ATAC sequencing reads. Diagrams of L1-0518 **(S)**, L1-1501 **(T)**, L1-1960 **(U)**, L1-4228 **(V)**, L1-4249 **(W)**, L1-4821 **(X)**, L1-4938 **(Y)**, L1-5330 **(Z)**, and L1-5742 **(AA)** represent nine of ten random unexpressed loci in MCF7 cells. Random loci were selected to ensure representation from each L1PA1-8 subfamily. One transitional L1Hs locus, L1-1337, was excluded from this analysis due to poor mappability. Each diagram includes information regarding the locus location (chromosome, start site, end site, and orientation), RNA-seq reads, presence of an ATAC peak, L1 sub-family, and number of reads from ATAC sequencing. Loops indicate CTCF binding sites (purple, shaded) within 500 kb of the L1 start site (black arrow). RNA polymerase II (Pol II) loops (red) are only shown if the pol II binding site overlaps within 500bp of the L1 start site. The CTCF loops are shown at a scale of 500kb upstream and downstream of the L1 start site. Red arrows indicate magnification of the indicated genomic region, which contains information regarding the presence of the activating histone marks from previously described CHIP-seq data (Figure 3A). Results of analysis of all histone marks are shown from two experiments for both the L1 promoter and putative enhancer ends of the RNA polymerase II loops.


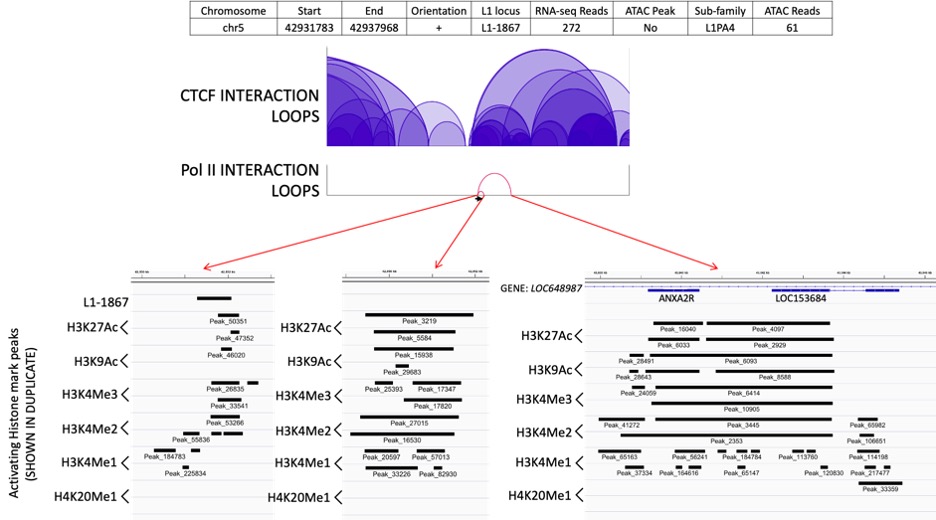


**Supplemental Figure 6C**

**Supplemental Figure 6. Long-distance interactions of individual L1-loci in MCF7 cells. (A-AA)** Diagrams of L1-0728 **(A)**, L1-3682 **(B)**, L1-1867 **(C)**, L1-2476 **(D)**, L1-3165 **(E)**, L1-0029 **(F)**, L1-3455 **(G)**, and L1-1685 **(H)**, and L1-3239 **(I)** represent nine of the ten highest expressed loci in MCF7 cells. Diagrams of L1-2855 **(J)**, L1-0225 **(K)**, L1-0986 **(L)**, L1-4910 **(M)**, L1-5151 **(N)**, L1-1469 **(O)**, L1-0482 **(P)**, L1-3525 **(Q)**, and L1-4180 **(R)** represent nine of the ten transitional loci (unexpressed loci overlapping with an ATAC peak) with the largest peaks produced by mapping of ATAC sequencing reads. Diagrams of L1-0518 **(S)**, L1-1501 **(T)**, L1-1960 **(U)**, L1-4228 **(V)**, L1-4249 **(W)**, L1-4821 **(X)**, L1-4938 **(Y)**, L1-5330 **(Z)**, and L1-5742 **(AA)** represent nine of ten random unexpressed loci in MCF7 cells. Random loci were selected to ensure representation from each L1PA1-8 subfamily. One transitional L1Hs locus, L1-1337, was excluded from this analysis due to poor mappability. Each diagram includes information regarding the locus location (chromosome, start site, end site, and orientation), RNA-seq reads, presence of an ATAC peak, L1 sub-family, and number of reads from ATAC sequencing. Loops indicate CTCF binding sites (purple, shaded) within 500 kb of the L1 start site (black arrow). RNA polymerase II (Pol II) loops (red) are only shown if the pol II binding site overlaps within 500bp of the L1 start site. The CTCF loops are shown at a scale of 500kb upstream and downstream of the L1 start site. Red arrows indicate magnification of the indicated genomic region, which contains information regarding the presence of the activating histone marks from previously described CHIP-seq data (Figure 3A). Results of analysis of all histone marks are shown from two experiments for both the L1 promoter and putative enhancer ends of the RNA polymerase II loops.


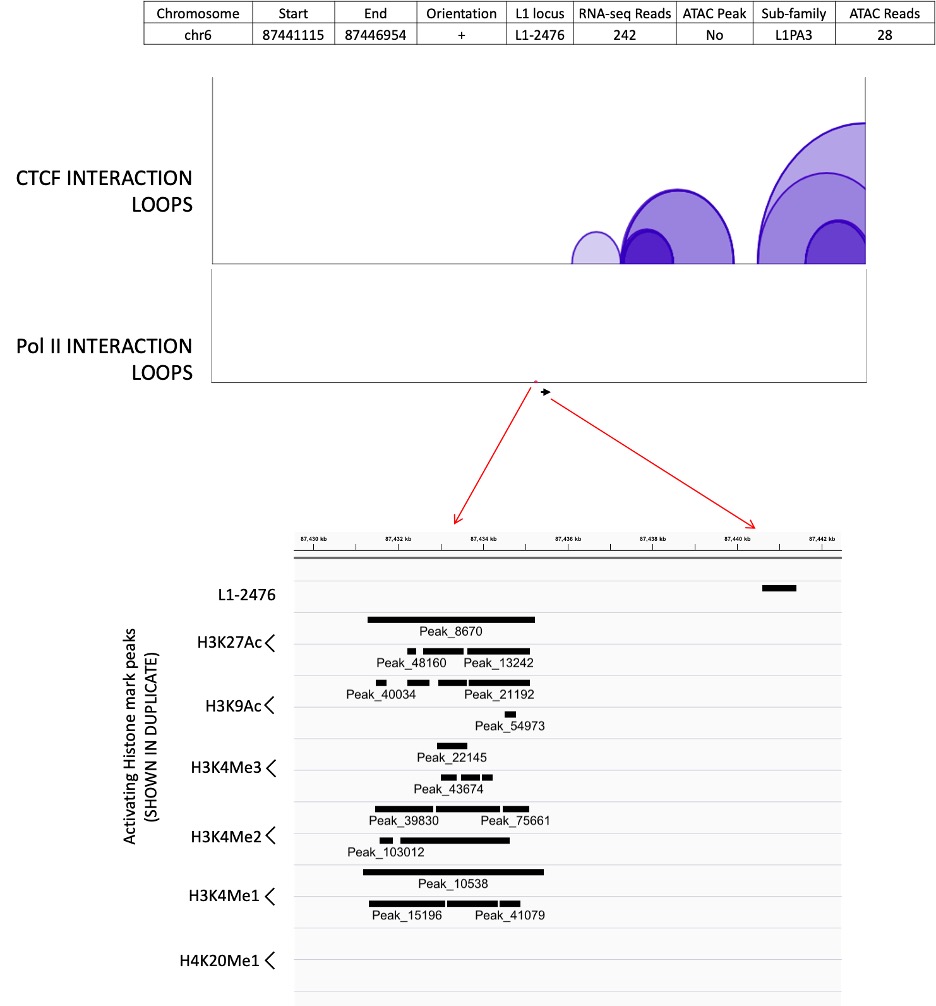


**Supplemental Figure 6D**

**Supplemental Figure 6. Long-distance interactions of individual L1-loci in MCF7 cells. (A-AA)** Diagrams of L1-0728 **(A)**, L1-3682 **(B)**, L1-1867 **(C)**, L1-2476 **(D)**, L1-3165 **(E)**, L1-0029 **(F)**, L1-3455 **(G)**, and L1-1685 **(H)**, and L1-3239 **(I)** represent nine of the ten highest expressed loci in MCF7 cells. Diagrams of L1-2855 **(J)**, L1-0225 **(K)**, L1-0986 **(L)**, L1-4910 **(M)**, L1-5151 **(N)**, L1-1469 **(O)**, L1-0482 **(P)**, L1-3525 **(Q)**, and L1-4180 **(R)** represent nine of the ten transitional loci (unexpressed loci overlapping with an ATAC peak) with the largest peaks produced by mapping of ATAC sequencing reads. Diagrams of L1-0518 **(S)**, L1-1501 **(T)**, L1-1960 **(U)**, L1-4228 **(V)**, L1-4249 **(W)**, L1-4821 **(X)**, L1-4938 **(Y)**, L1-5330 **(Z)**, and L1-5742 **(AA)** represent nine of ten random unexpressed loci in MCF7 cells. Random loci were selected to ensure representation from each L1PA1-8 subfamily. One transitional L1Hs locus, L1-1337, was excluded from this analysis due to poor mappability. Each diagram includes information regarding the locus location (chromosome, start site, end site, and orientation), RNA-seq reads, presence of an ATAC peak, L1 sub-family, and number of reads from ATAC sequencing. Loops indicate CTCF binding sites (purple, shaded) within 500 kb of the L1 start site (black arrow). RNA polymerase II (Pol II) loops (red) are only shown if the pol II binding site overlaps within 500bp of the L1 start site. The CTCF loops are shown at a scale of 500kb upstream and downstream of the L1 start site. Red arrows indicate magnification of the indicated genomic region, which contains information regarding the presence of the activating histone marks from previously described CHIP-seq data (Figure 3A). Results of analysis of all histone marks are shown from two experiments for both the L1 promoter and putative enhancer ends of the RNA polymerase II loops.


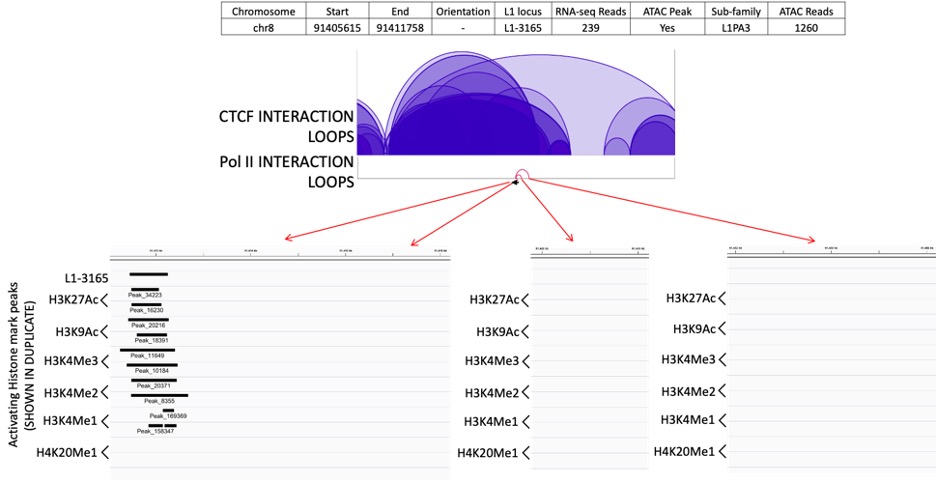


**Supplemental Figure 6E**

**Supplemental Figure 6. Long-distance interactions of individual L1-loci in MCF7 cells. (A-AA)** Diagrams of L1-0728 **(A)**, L1-3682 **(B)**, L1-1867 **(C)**, L1-2476 **(D)**, L1-3165 **(E)**, L1-0029 **(F)**, L1-3455 **(G)**, and L1-1685 **(H)**, and L1-3239 **(I)** represent nine of the ten highest expressed loci in MCF7 cells. Diagrams of L1-2855 **(J)**, L1-0225 **(K)**, L1-0986 **(L)**, L1-4910 **(M)**, L1-5151 **(N)**, L1-1469 **(O)**, L1-0482 **(P)**, L1-3525 **(Q)**, and L1-4180 **(R)** represent nine of the ten transitional loci (unexpressed loci overlapping with an ATAC peak) with the largest peaks produced by mapping of ATAC sequencing reads. Diagrams of L1-0518 **(S)**, L1-1501 **(T)**, L1-1960 **(U)**, L1-4228 **(V)**, L1-4249 **(W)**, L1-4821 **(X)**, L1-4938 **(Y)**, L1-5330 **(Z)**, and L1-5742 **(AA)** represent nine of ten random unexpressed loci in MCF7 cells. Random loci were selected to ensure representation from each L1PA1-8 subfamily. One transitional L1Hs locus, L1-1337, was excluded from this analysis due to poor mappability. Each diagram includes information regarding the locus location (chromosome, start site, end site, and orientation), RNA-seq reads, presence of an ATAC peak, L1 sub-family, and number of reads from ATAC sequencing. Loops indicate CTCF binding sites (purple, shaded) within 500 kb of the L1 start site (black arrow). RNA polymerase II (Pol II) loops (red) are only shown if the pol II binding site overlaps within 500bp of the L1 start site. The CTCF loops are shown at a scale of 500kb upstream and downstream of the L1 start site. Red arrows indicate magnification of the indicated genomic region, which contains information regarding the presence of the activating histone marks from previously described CHIP-seq data (Figure 3A). Results of analysis of all histone marks are shown from two experiments for both the L1 promoter and putative enhancer ends of the RNA polymerase II loops.


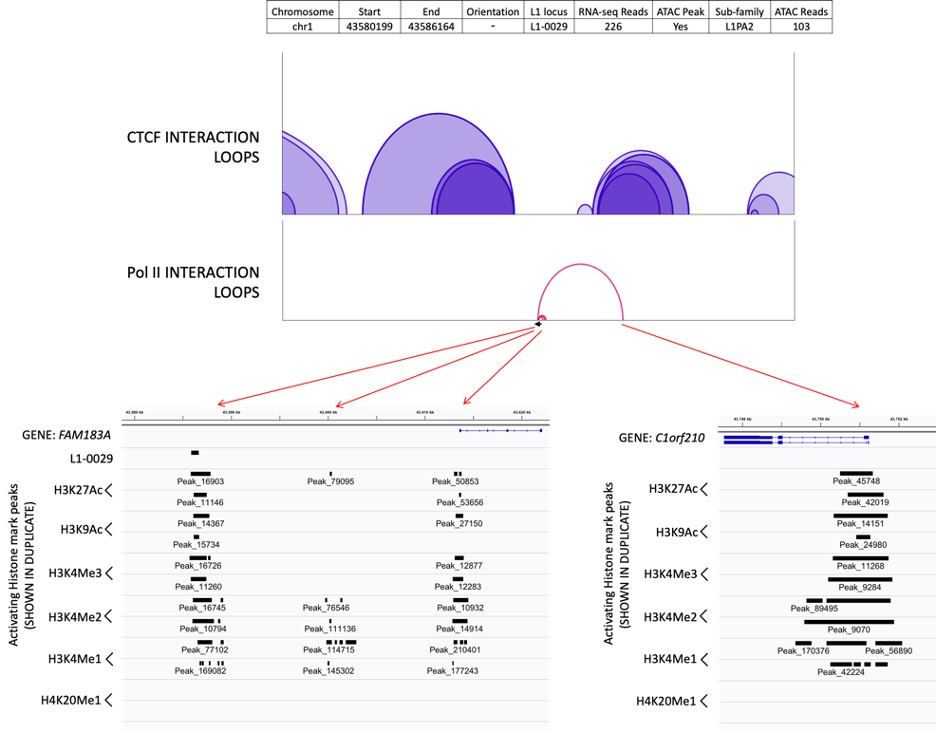


**Supplemental Figure 6F**

**Supplemental Figure 6. Long-distance interactions of individual L1-loci in MCF7 cells. (A-AA)** Diagrams of L1-0728 **(A)**, L1-3682 **(B)**, L1-1867 **(C)**, L1-2476 **(D)**, L1-3165 **(E)**, L1-0029 **(F)**, L1-3455 **(G)**, and L1-1685 **(H)**, and L1-3239 **(I)** represent nine of the ten highest expressed loci in MCF7 cells. Diagrams of L1-2855 **(J)**, L1-0225 **(K)**, L1-0986 **(L)**, L1-4910 **(M)**, L1-5151 **(N)**, L1-1469 **(O)**, L1-0482 **(P)**, L1-3525 **(Q)**, and L1-4180 **(R)** represent nine of the ten transitional loci (unexpressed loci overlapping with an ATAC peak) with the largest peaks produced by mapping of ATAC sequencing reads. Diagrams of L1-0518 **(S)**, L1-1501 **(T)**, L1-1960 **(U)**, L1-4228 **(V)**, L1-4249 **(W)**, L1-4821 **(X)**, L1-4938 **(Y)**, L1-5330 **(Z)**, and L1-5742 **(AA)** represent nine of ten random unexpressed loci in MCF7 cells. Random loci were selected to ensure representation from each L1PA1-8 subfamily. One transitional L1Hs locus, L1-1337, was excluded from this analysis due to poor mappability. Each diagram includes information regarding the locus location (chromosome, start site, end site, and orientation), RNA-seq reads, presence of an ATAC peak, L1 sub-family, and number of reads from ATAC sequencing. Loops indicate CTCF binding sites (purple, shaded) within 500 kb of the L1 start site (black arrow). RNA polymerase II (Pol II) loops (red) are only shown if the pol II binding site overlaps within 500bp of the L1 start site. The CTCF loops are shown at a scale of 500kb upstream and downstream of the L1 start site. Red arrows indicate magnification of the indicated genomic region, which contains information regarding the presence of the activating histone marks from previously described CHIP-seq data (Figure 3A). Results of analysis of all histone marks are shown from two experiments for both the L1 promoter and putative enhancer ends of the RNA polymerase II loops.


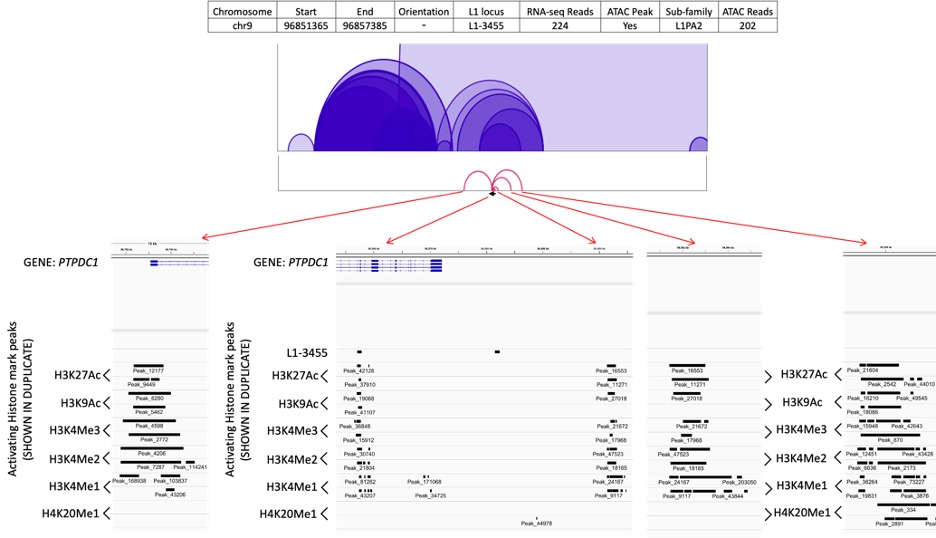


**Supplemental Figure 6G**

**Supplemental Figure 6. Long-distance interactions of individual L1-loci in MCF7 cells. (A-AA)** Diagrams of L1-0728 **(A)**, L1-3682 **(B)**, L1-1867 **(C)**, L1-2476 **(D)**, L1-3165 **(E)**, L1-0029 **(F)**, L1-3455 **(G)**, and L1-1685 **(H)**, and L1-3239 **(I)** represent nine of the ten highest expressed loci in MCF7 cells. Diagrams of L1-2855 **(J)**, L1-0225 **(K)**, L1-0986 **(L)**, L1-4910 **(M)**, L1-5151 **(N)**, L1-1469 **(O)**, L1-0482 **(P)**, L1-3525 **(Q)**, and L1-4180 **(R)** represent nine of the ten transitional loci (unexpressed loci overlapping with an ATAC peak) with the largest peaks produced by mapping of ATAC sequencing reads. Diagrams of L1-0518 **(S)**, L1-1501 **(T)**, L1-1960 **(U)**, L1-4228 **(V)**, L1-4249 **(W)**, L1-4821 **(X)**, L1-4938 **(Y)**, L1-5330 **(Z)**, and L1-5742 **(AA)** represent nine of ten random unexpressed loci in MCF7 cells. Random loci were selected to ensure representation from each L1PA1-8 subfamily. One transitional L1Hs locus, L1-1337, was excluded from this analysis due to poor mappability. Each diagram includes information regarding the locus location (chromosome, start site, end site, and orientation), RNA-seq reads, presence of an ATAC peak, L1 sub-family, and number of reads from ATAC sequencing. Loops indicate CTCF binding sites (purple, shaded) within 500 kb of the L1 start site (black arrow). RNA polymerase II (Pol II) loops (red) are only shown if the pol II binding site overlaps within 500bp of the L1 start site. The CTCF loops are shown at a scale of 500kb upstream and downstream of the L1 start site. Red arrows indicate magnification of the indicated genomic region, which contains information regarding the presence of the activating histone marks from previously described CHIP-seq data (Figure 3A). Results of analysis of all histone marks are shown from two experiments for both the L1 promoter and putative enhancer ends of the RNA polymerase II loops.


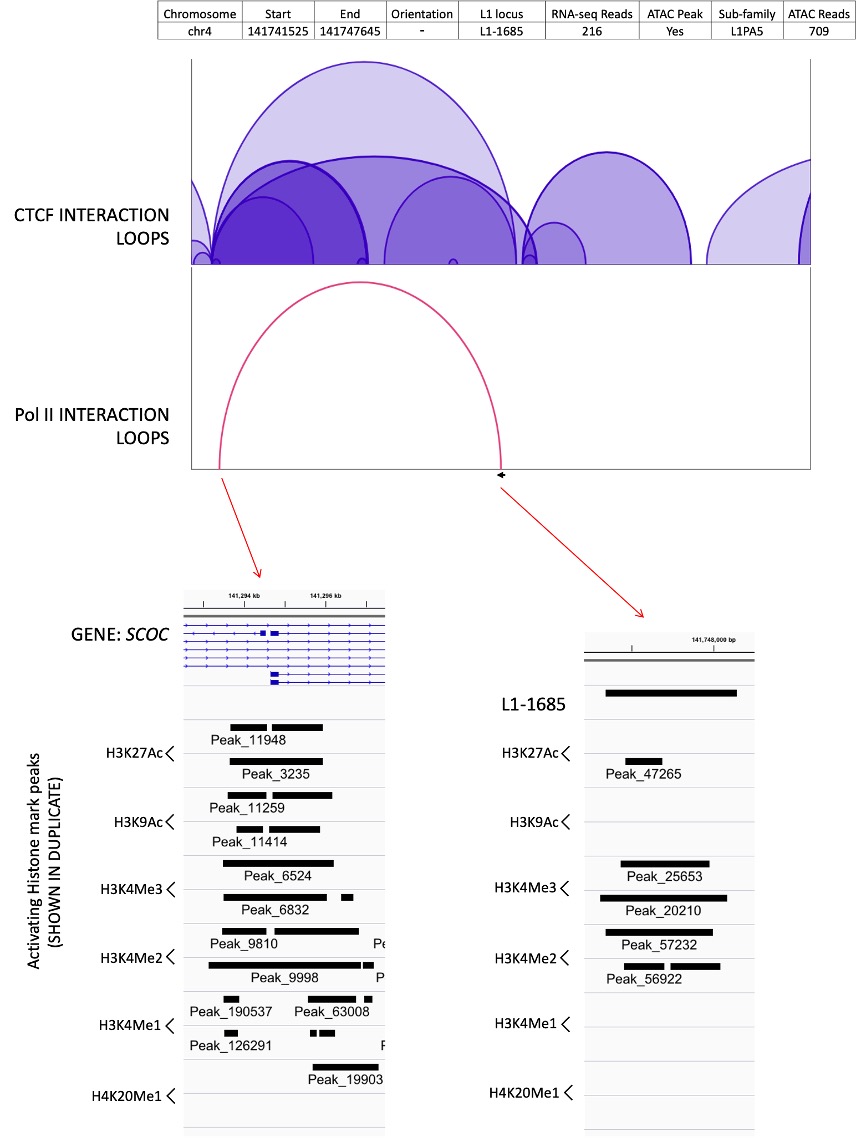


**Supplemental Figure 6H**

**Supplemental Figure 6. Long-distance interactions of individual L1-loci in MCF7 cells. (A-AA)** Diagrams of L1-0728 **(A)**, L1-3682 **(B)**, L1-1867 **(C)**, L1-2476 **(D)**, L1-3165 **(E)**, L1-0029 **(F)**, L1-3455 **(G)**, and L1-1685 **(H)**, and L1-3239 **(I)** represent nine of the ten highest expressed loci in MCF7 cells. Diagrams of L1-2855 **(J)**, L1-0225 **(K)**, L1-0986 **(L)**, L1-4910 **(M)**, L1-5151 **(N)**, L1-1469 **(O)**, L1-0482 **(P)**, L1-3525 **(Q)**, and L1-4180 **(R)** represent nine of the ten transitional loci (unexpressed loci overlapping with an ATAC peak) with the largest peaks produced by mapping of ATAC sequencing reads. Diagrams of L1-0518 **(S)**, L1-1501 **(T)**, L1-1960 **(U)**, L1-4228 **(V)**, L1-4249 **(W)**, L1-4821 **(X)**, L1-4938 **(Y)**, L1-5330 **(Z)**, and L1-5742 **(AA)** represent nine of ten random unexpressed loci in MCF7 cells. Random loci were selected to ensure representation from each L1PA1-8 subfamily. One transitional L1Hs locus, L1-1337, was excluded from this analysis due to poor mappability. Each diagram includes information regarding the locus location (chromosome, start site, end site, and orientation), RNA-seq reads, presence of an ATAC peak, L1 sub-family, and number of reads from ATAC sequencing. Loops indicate CTCF binding sites (purple, shaded) within 500 kb of the L1 start site (black arrow). RNA polymerase II (Pol II) loops (red) are only shown if the pol II binding site overlaps within 500bp of the L1 start site. The CTCF loops are shown at a scale of 500kb upstream and downstream of the L1 start site. Red arrows indicate magnification of the indicated genomic region, which contains information regarding the presence of the activating histone marks from previously described CHIP-seq data (Figure 3A). Results of analysis of all histone marks are shown from two experiments for both the L1 promoter and putative enhancer ends of the RNA polymerase II loops.


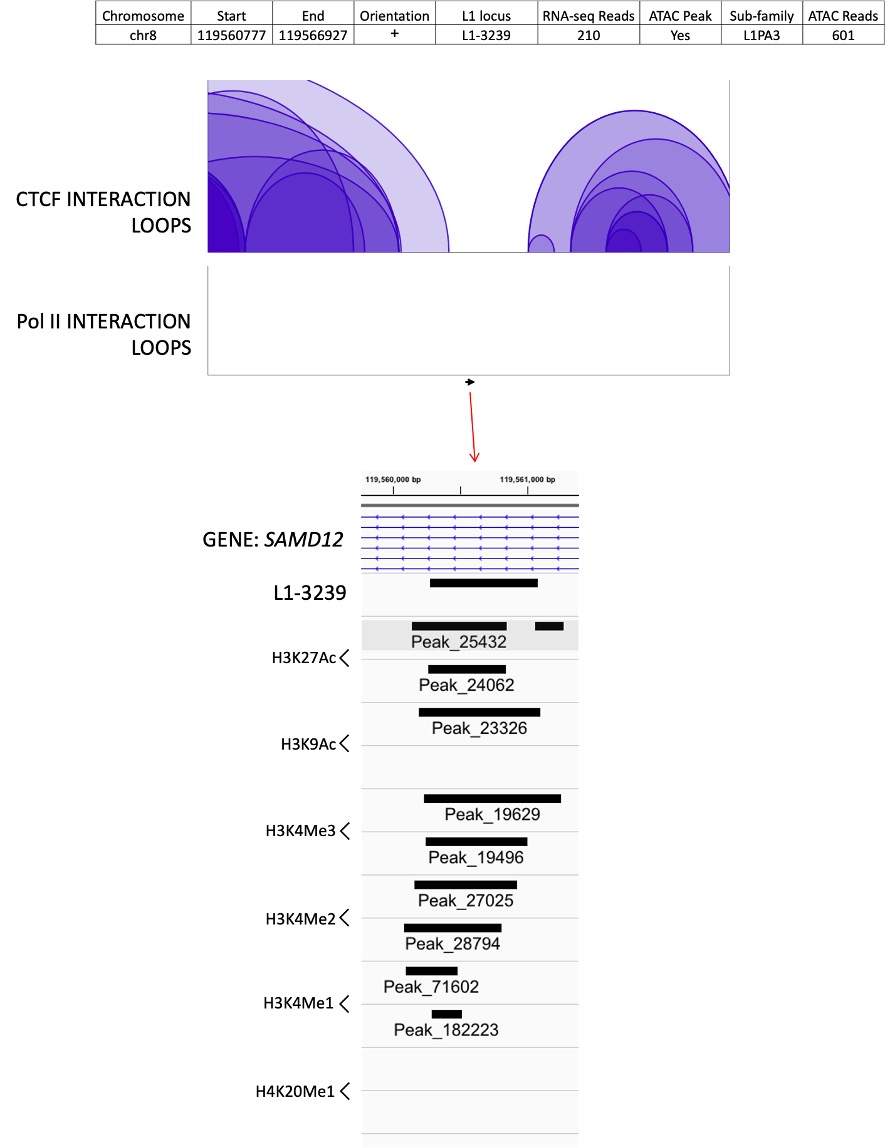


**Supplemental Figure 6I**

**Supplemental Figure 6. Long-distance interactions of individual L1-loci in MCF7 cells. (A-AA)** Diagrams of L1-0728 **(A)**, L1-3682 **(B)**, L1-1867 **(C)**, L1-2476 **(D)**, L1-3165 **(E)**, L1-0029 **(F)**, L1-3455 **(G)**, and L1-1685 **(H)**, and L1-3239 **(I)** represent nine of the ten highest expressed loci in MCF7 cells. Diagrams of L1-2855 **(J)**, L1-0225 **(K)**, L1-0986 **(L)**, L1-4910 **(M)**, L1-5151 **(N)**, L1-1469 **(O)**, L1-0482 **(P)**, L1-3525 **(Q)**, and L1-4180 **(R)** represent nine of the ten transitional loci (unexpressed loci overlapping with an ATAC peak) with the largest peaks produced by mapping of ATAC sequencing reads. Diagrams of L1-0518 **(S)**, L1-1501 **(T)**, L1-1960 **(U)**, L1-4228 **(V)**, L1-4249 **(W)**, L1-4821 **(X)**, L1-4938 **(Y)**, L1-5330 **(Z)**, and L1-5742 **(AA)** represent nine of ten random unexpressed loci in MCF7 cells. Random loci were selected to ensure representation from each L1PA1-8 subfamily. One transitional L1Hs locus, L1-1337, was excluded from this analysis due to poor mappability. Each diagram includes information regarding the locus location (chromosome, start site, end site, and orientation), RNA-seq reads, presence of an ATAC peak, L1 sub-family, and number of reads from ATAC sequencing. Loops indicate CTCF binding sites (purple, shaded) within 500 kb of the L1 start site (black arrow). RNA polymerase II (Pol II) loops (red) are only shown if the pol II binding site overlaps within 500bp of the L1 start site. The CTCF loops are shown at a scale of 500kb upstream and downstream of the L1 start site. Red arrows indicate magnification of the indicated genomic region, which contains information regarding the presence of the activating histone marks from previously described CHIP-seq data (Figure 3A). Results of analysis of all histone marks are shown from two experiments for both the L1 promoter and putative enhancer ends of the RNA polymerase II loops.


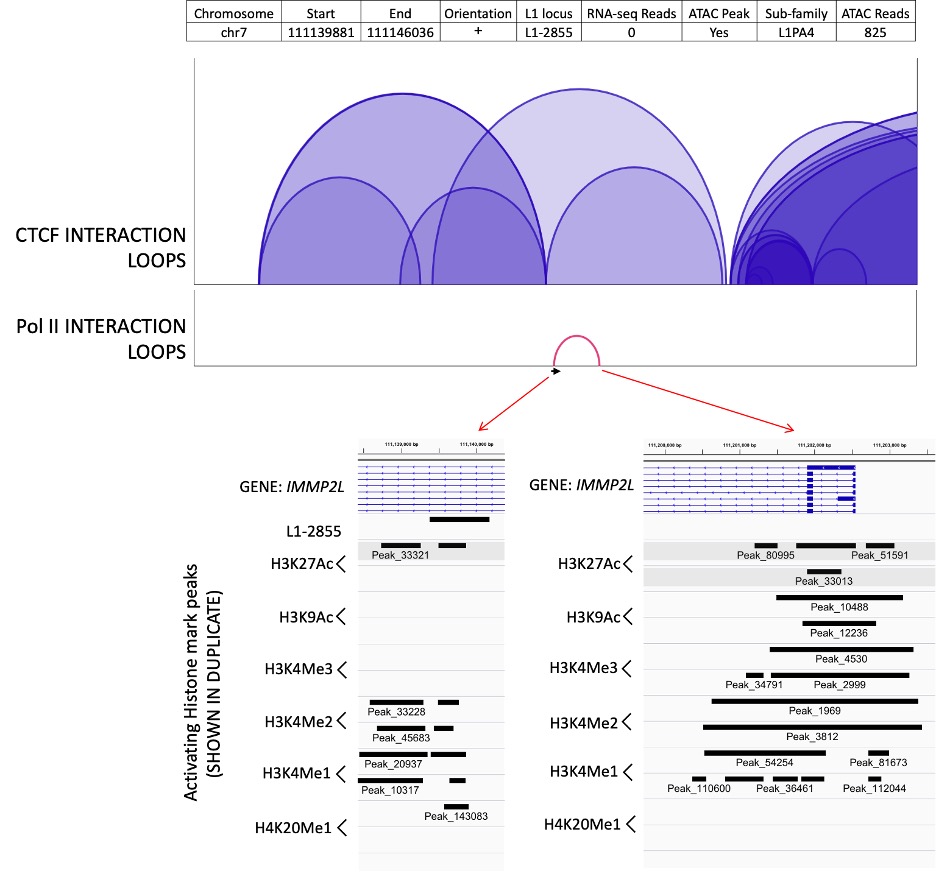


**Supplemental Figure 6J**

**Supplemental Figure 6. Long-distance interactions of individual L1-loci in MCF7 cells. (A-AA)** Diagrams of L1-0728 **(A)**, L1-3682 **(B)**, L1-1867 **(C)**, L1-2476 **(D)**, L1-3165 **(E)**, L1-0029 **(F)**, L1-3455 **(G)**, and L1-1685 **(H)**, and L1-3239 **(I)** represent nine of the ten highest expressed loci in MCF7 cells. Diagrams of L1-2855 **(J)**, L1-0225 **(K)**, L1-0986 **(L)**, L1-4910 **(M)**, L1-5151 **(N)**, L1-1469 **(O)**, L1-0482 **(P)**, L1-3525 **(Q)**, and L1-4180 **(R)** represent nine of the ten transitional loci (unexpressed loci overlapping with an ATAC peak) with the largest peaks produced by mapping of ATAC sequencing reads. Diagrams of L1-0518 **(S)**, L1-1501 **(T)**, L1-1960 **(U)**, L1-4228 **(V)**, L1-4249 **(W)**, L1-4821 **(X)**, L1-4938 **(Y)**, L1-5330 **(Z)**, and L1-5742 **(AA)** represent nine of ten random unexpressed loci in MCF7 cells. Random loci were selected to ensure representation from each L1PA1-8 subfamily. One transitional L1Hs locus, L1-1337, was excluded from this analysis due to poor mappability. Each diagram includes information regarding the locus location (chromosome, start site, end site, and orientation), RNA-seq reads, presence of an ATAC peak, L1 sub-family, and number of reads from ATAC sequencing. Loops indicate CTCF binding sites (purple, shaded) within 500 kb of the L1 start site (black arrow). RNA polymerase II (Pol II) loops (red) are only shown if the pol II binding site overlaps within 500bp of the L1 start site. The CTCF loops are shown at a scale of 500kb upstream and downstream of the L1 start site. Red arrows indicate magnification of the indicated genomic region, which contains information regarding the presence of the activating histone marks from previously described CHIP-seq data (Figure 3A). Results of analysis of all histone marks are shown from two experiments for both the L1 promoter and putative enhancer ends of the RNA polymerase II loops.


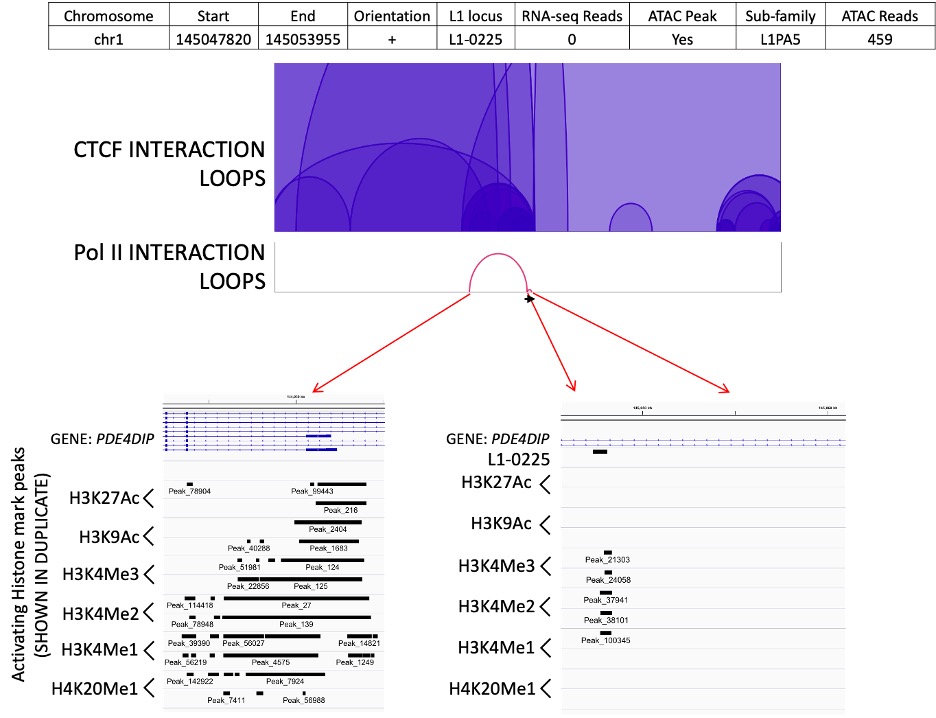


**Supplemental Figure 6K**

**Supplemental Figure 6. Long-distance interactions of individual L1-loci in MCF7 cells. (A-AA)** Diagrams of L1-0728 **(A)**, L1-3682 **(B)**, L1-1867 **(C)**, L1-2476 **(D)**, L1-3165 **(E)**, L1-0029 **(F)**, L1-3455 **(G)**, and L1-1685 **(H)**, and L1-3239 **(I)** represent nine of the ten highest expressed loci in MCF7 cells. Diagrams of L1-2855 **(J)**, L1-0225 **(K)**, L1-0986 **(L)**, L1-4910 **(M)**, L1-5151 **(N)**, L1-1469 **(O)**, L1-0482 **(P)**, L1-3525 **(Q)**, and L1-4180 **(R)** represent nine of the ten transitional loci (unexpressed loci overlapping with an ATAC peak) with the largest peaks produced by mapping of ATAC sequencing reads. Diagrams of L1-0518 **(S)**, L1-1501 **(T)**, L1-1960 **(U)**, L1-4228 **(V)**, L1-4249 **(W)**, L1-4821 **(X)**, L1-4938 **(Y)**, L1-5330 **(Z)**, and L1-5742 **(AA)** represent nine of ten random unexpressed loci in MCF7 cells. Random loci were selected to ensure representation from each L1PA1-8 subfamily. One transitional L1Hs locus, L1-1337, was excluded from this analysis due to poor mappability. Each diagram includes information regarding the locus location (chromosome, start site, end site, and orientation), RNA-seq reads, presence of an ATAC peak, L1 sub-family, and number of reads from ATAC sequencing. Loops indicate CTCF binding sites (purple, shaded) within 500 kb of the L1 start site (black arrow). RNA polymerase II (Pol II) loops (red) are only shown if the pol II binding site overlaps within 500bp of the L1 start site. The CTCF loops are shown at a scale of 500kb upstream and downstream of the L1 start site. Red arrows indicate magnification of the indicated genomic region, which contains information regarding the presence of the activating histone marks from previously described CHIP-seq data (Figure 3A). Results of analysis of all histone marks are shown from two experiments for both the L1 promoter and putative enhancer ends of the RNA polymerase II loops.


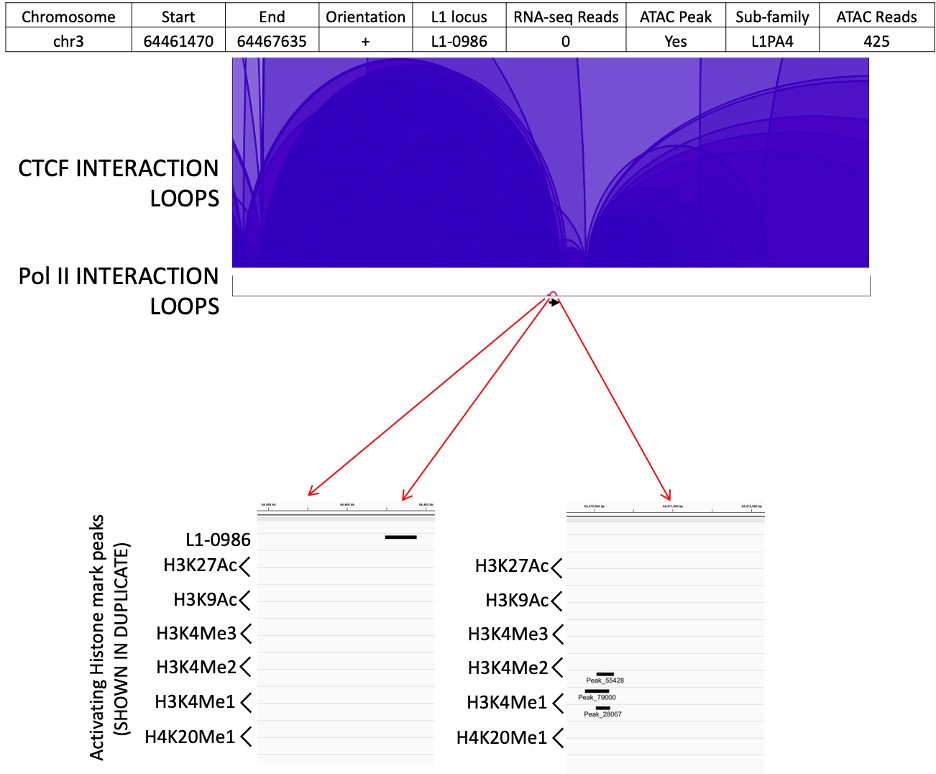


**Supplemental Figure 6L**

**Supplemental Figure 6. Long-distance interactions of individual L1-loci in MCF7 cells. (A-AA)** Diagrams of L1-0728 **(A)**, L1-3682 **(B)**, L1-1867 **(C)**, L1-2476 **(D)**, L1-3165 **(E)**, L1-0029 **(F)**, L1-3455 **(G)**, and L1-1685 **(H)**, and L1-3239 **(I)** represent nine of the ten highest expressed loci in MCF7 cells. Diagrams of L1-2855 **(J)**, L1-0225 **(K)**, L1-0986 **(L)**, L1-4910 **(M)**, L1-5151 **(N)**, L1-1469 **(O)**, L1-0482 **(P)**, L1-3525 **(Q)**, and L1-4180 **(R)** represent nine of the ten transitional loci (unexpressed loci overlapping with an ATAC peak) with the largest peaks produced by mapping of ATAC sequencing reads. Diagrams of L1-0518 **(S)**, L1-1501 **(T)**, L1-1960 **(U)**, L1-4228 **(V)**, L1-4249 **(W)**, L1-4821 **(X)**, L1-4938 **(Y)**, L1-5330 **(Z)**, and L1-5742 **(AA)** represent nine of ten random unexpressed loci in MCF7 cells. Random loci were selected to ensure representation from each L1PA1-8 subfamily. One transitional L1Hs locus, L1-1337, was excluded from this analysis due to poor mappability. Each diagram includes information regarding the locus location (chromosome, start site, end site, and orientation), RNA-seq reads, presence of an ATAC peak, L1 sub-family, and number of reads from ATAC sequencing. Loops indicate CTCF binding sites (purple, shaded) within 500 kb of the L1 start site (black arrow). RNA polymerase II (Pol II) loops (red) are only shown if the pol II binding site overlaps within 500bp of the L1 start site. The CTCF loops are shown at a scale of 500kb upstream and downstream of the L1 start site. Red arrows indicate magnification of the indicated genomic region, which contains information regarding the presence of the activating histone marks from previously described CHIP-seq data (Figure 3A). Results of analysis of all histone marks are shown from two experiments for both the L1 promoter and putative enhancer ends of the RNA polymerase II loops.


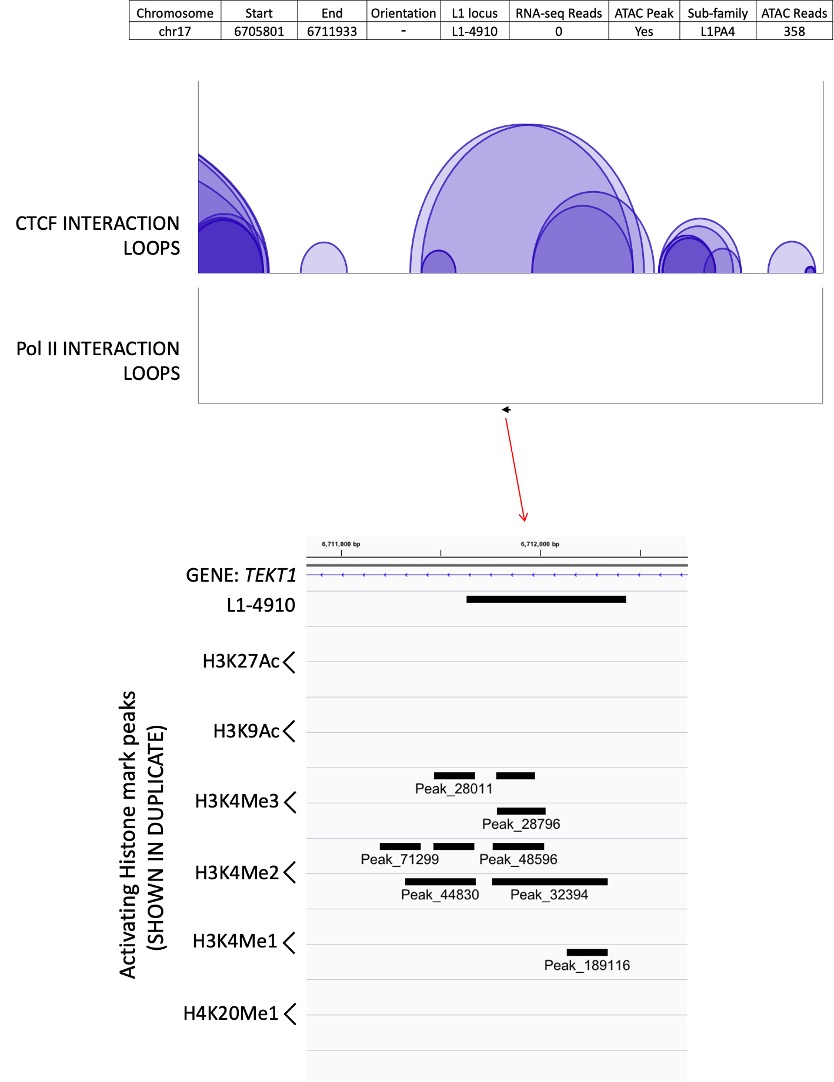


Supplemental Figure 6M

**Supplemental Figure 6. Long-distance interactions of individual L1-loci in MCF7 cells. (A-AA)** Diagrams of L1-0728 **(A)**, L1-3682 **(B)**, L1-1867 **(C)**, L1-2476 **(D)**, L1-3165 **(E)**, L1-0029 **(F)**, L1-3455 **(G)**, and L1-1685 **(H)**, and L1-3239 **(I)** represent nine of the ten highest expressed loci in MCF7 cells. Diagrams of L1-2855 **(J)**, L1-0225 **(K)**, L1-0986 **(L)**, L1-4910 **(M)**, L1-5151 **(N)**, L1-1469 **(O)**, L1-0482 **(P)**, L1-3525 **(Q)**, and L1-4180 **(R)** represent nine of the ten transitional loci (unexpressed loci overlapping with an ATAC peak) with the largest peaks produced by mapping of ATAC sequencing reads. Diagrams of L1-0518 **(S)**, L1-1501 **(T)**, L1-1960 **(U)**, L1-4228 **(V)**, L1-4249 **(W)**, L1-4821 **(X)**, L1-4938 **(Y)**, L1-5330 **(Z)**, and L1-5742 **(AA)** represent nine of ten random unexpressed loci in MCF7 cells. Random loci were selected to ensure representation from each L1PA1-8 subfamily. One transitional L1Hs locus, L1-1337, was excluded from this analysis due to poor mappability. Each diagram includes information regarding the locus location (chromosome, start site, end site, and orientation), RNA-seq reads, presence of an ATAC peak, L1 sub-family, and number of reads from ATAC sequencing. Loops indicate CTCF binding sites (purple, shaded) within 500 kb of the L1 start site (black arrow). RNA polymerase II (Pol II) loops (red) are only shown if the pol II binding site overlaps within 500bp of the L1 start site. The CTCF loops are shown at a scale of 500kb upstream and downstream of the L1 start site. Red arrows indicate magnification of the indicated genomic region, which contains information regarding the presence of the activating histone marks from previously described CHIP-seq data (Figure 3A). Results of analysis of all histone marks are shown from two experiments for both the L1 promoter and putative enhancer ends of the RNA polymerase II loops.


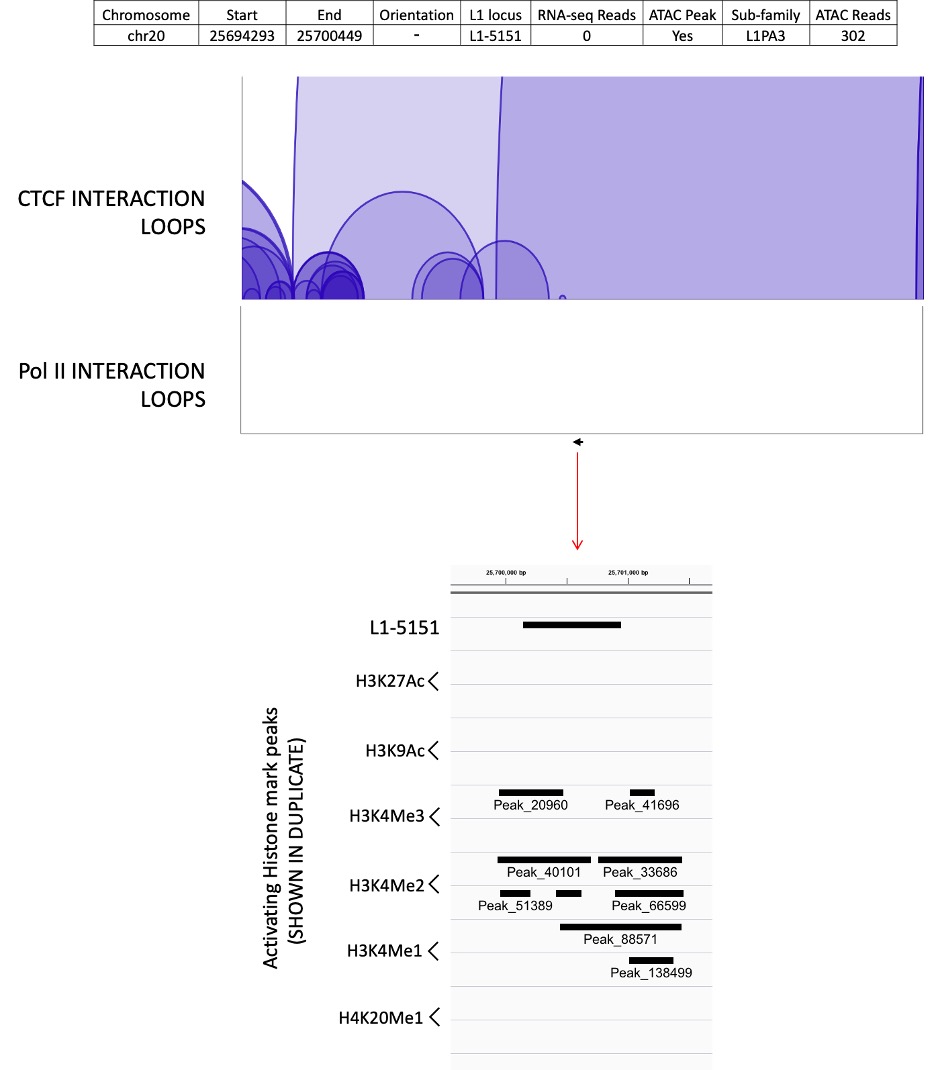


**Supplemental Figure 6N**

**Supplemental Figure 6. Long-distance interactions of individual L1-loci in MCF7 cells. (A-AA)** Diagrams of L1-0728 **(A)**, L1-3682 **(B)**, L1-1867 **(C)**, L1-2476 **(D)**, L1-3165 **(E)**, L1-0029 **(F)**, L1-3455 **(G)**, and L1-1685 **(H)**, and L1-3239 **(I)** represent nine of the ten highest expressed loci in MCF7 cells. Diagrams of L1-2855 **(J)**, L1-0225 **(K)**, L1-0986 **(L)**, L1-4910 **(M)**, L1-5151 **(N)**, L1-1469 **(O)**, L1-0482 **(P)**, L1-3525 **(Q)**, and L1-4180 **(R)** represent nine of the ten transitional loci (unexpressed loci overlapping with an ATAC peak) with the largest peaks produced by mapping of ATAC sequencing reads. Diagrams of L1-0518 **(S)**, L1-1501 **(T)**, L1-1960 **(U)**, L1-4228 **(V)**, L1-4249 **(W)**, L1-4821 **(X)**, L1-4938 **(Y)**, L1-5330 **(Z)**, and L1-5742 **(AA)** represent nine of ten random unexpressed loci in MCF7 cells. Random loci were selected to ensure representation from each L1PA1-8 subfamily. One transitional L1Hs locus, L1-1337, was excluded from this analysis due to poor mappability. Each diagram includes information regarding the locus location (chromosome, start site, end site, and orientation), RNA-seq reads, presence of an ATAC peak, L1 sub-family, and number of reads from ATAC sequencing. Loops indicate CTCF binding sites (purple, shaded) within 500 kb of the L1 start site (black arrow). RNA polymerase II (Pol II) loops (red) are only shown if the pol II binding site overlaps within 500bp of the L1 start site. The CTCF loops are shown at a scale of 500kb upstream and downstream of the L1 start site. Red arrows indicate magnification of the indicated genomic region, which contains information regarding the presence of the activating histone marks from previously described CHIP-seq data (Figure 3A). Results of analysis of all histone marks are shown from two experiments for both the L1 promoter and putative enhancer ends of the RNA polymerase II loops.


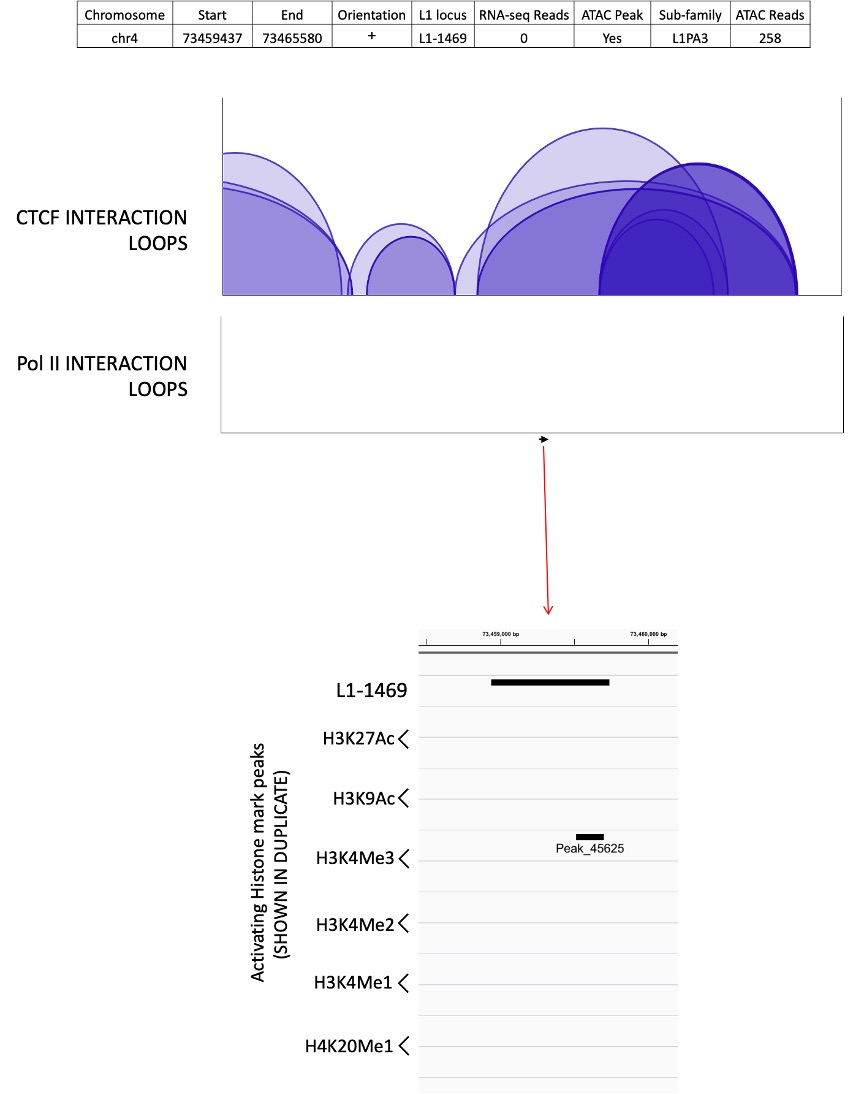


**Supplemental Figure 6O**

**Supplemental Figure 6. Long-distance interactions of individual L1-loci in MCF7 cells. (A-AA)** Diagrams of L1-0728 **(A)**, L1-3682 **(B)**, L1-1867 **(C)**, L1-2476 **(D)**, L1-3165 **(E)**, L1-0029 **(F)**, L1-3455 **(G)**, and L1-1685 **(H)**, and L1-3239 **(I)** represent nine of the ten highest expressed loci in MCF7 cells. Diagrams of L1-2855 **(J)**, L1-0225 **(K)**, L1-0986 **(L)**, L1-4910 **(M)**, L1-5151 **(N)**, L1-1469 **(O)**, L1-0482 **(P)**, L1-3525 **(Q)**, and L1-4180 **(R)** represent nine of the ten transitional loci (unexpressed loci overlapping with an ATAC peak) with the largest peaks produced by mapping of ATAC sequencing reads. Diagrams of L1-0518 **(S)**, L1-1501 **(T)**, L1-1960 **(U)**, L1-4228 **(V)**, L1-4249 **(W)**, L1-4821 **(X)**, L1-4938 **(Y)**, L1-5330 **(Z)**, and L1-5742 **(AA)** represent nine of ten random unexpressed loci in MCF7 cells. Random loci were selected to ensure representation from each L1PA1-8 subfamily. One transitional L1Hs locus, L1-1337, was excluded from this analysis due to poor mappability. Each diagram includes information regarding the locus location (chromosome, start site, end site, and orientation), RNA-seq reads, presence of an ATAC peak, L1 sub-family, and number of reads from ATAC sequencing. Loops indicate CTCF binding sites (purple, shaded) within 500 kb of the L1 start site (black arrow). RNA polymerase II (Pol II) loops (red) are only shown if the pol II binding site overlaps within 500bp of the L1 start site. The CTCF loops are shown at a scale of 500kb upstream and downstream of the L1 start site. Red arrows indicate magnification of the indicated genomic region, which contains information regarding the presence of the activating histone marks from previously described CHIP-seq data (Figure 3A). Results of analysis of all histone marks are shown from two experiments for both the L1 promoter and putative enhancer ends of the RNA polymerase II loops.


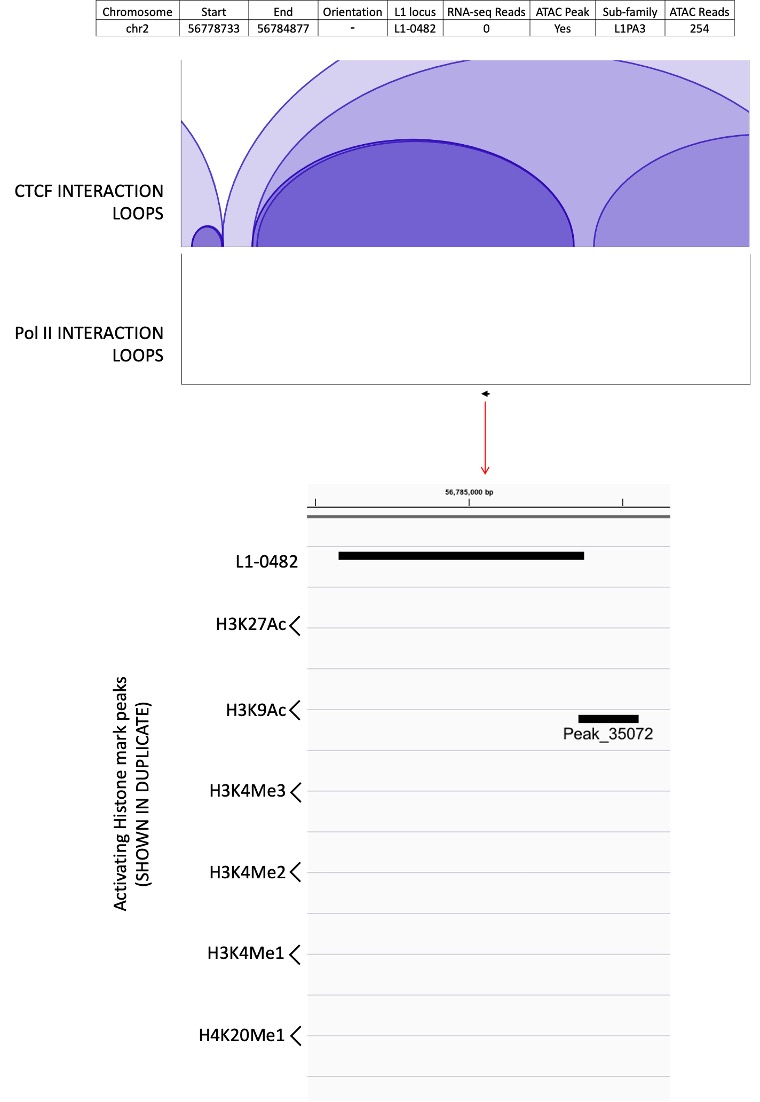


**Supplemental Figure 6P**

**Supplemental Figure 6. Long-distance interactions of individual L1-loci in MCF7 cells. (A-AA)** Diagrams of L1-0728 **(A)**, L1-3682 **(B)**, L1-1867 **(C)**, L1-2476 **(D)**, L1-3165 **(E)**, L1-0029 **(F)**, L1-3455 **(G)**, and L1-1685 **(H)**, and L1-3239 **(I)** represent nine of the ten highest expressed loci in MCF7 cells. Diagrams of L1-2855 **(J)**, L1-0225 **(K)**, L1-0986 **(L)**, L1-4910 **(M)**, L1-5151 **(N)**, L1-1469 **(O)**, L1-0482 **(P)**, L1-3525 **(Q)**, and L1-4180 **(R)** represent nine of the ten transitional loci (unexpressed loci overlapping with an ATAC peak) with the largest peaks produced by mapping of ATAC sequencing reads. Diagrams of L1-0518 **(S)**, L1-1501 **(T)**, L1-1960 **(U)**, L1-4228 **(V)**, L1-4249 **(W)**, L1-4821 **(X)**, L1-4938 **(Y)**, L1-5330 **(Z)**, and L1-5742 **(AA)** represent nine of ten random unexpressed loci in MCF7 cells. Random loci were selected to ensure representation from each L1PA1-8 subfamily. One transitional L1Hs locus, L1-1337, was excluded from this analysis due to poor mappability. Each diagram includes information regarding the locus location (chromosome, start site, end site, and orientation), RNA-seq reads, presence of an ATAC peak, L1 sub-family, and number of reads from ATAC sequencing. Loops indicate CTCF binding sites (purple, shaded) within 500 kb of the L1 start site (black arrow). RNA polymerase II (Pol II) loops (red) are only shown if the pol II binding site overlaps within 500bp of the L1 start site. The CTCF loops are shown at a scale of 500kb upstream and downstream of the L1 start site. Red arrows indicate magnification of the indicated genomic region, which contains information regarding the presence of the activating histone marks from previously described CHIP-seq data (Figure 3A). Results of analysis of all histone marks are shown from two experiments for both the L1 promoter and putative enhancer ends of the RNA polymerase II loops.


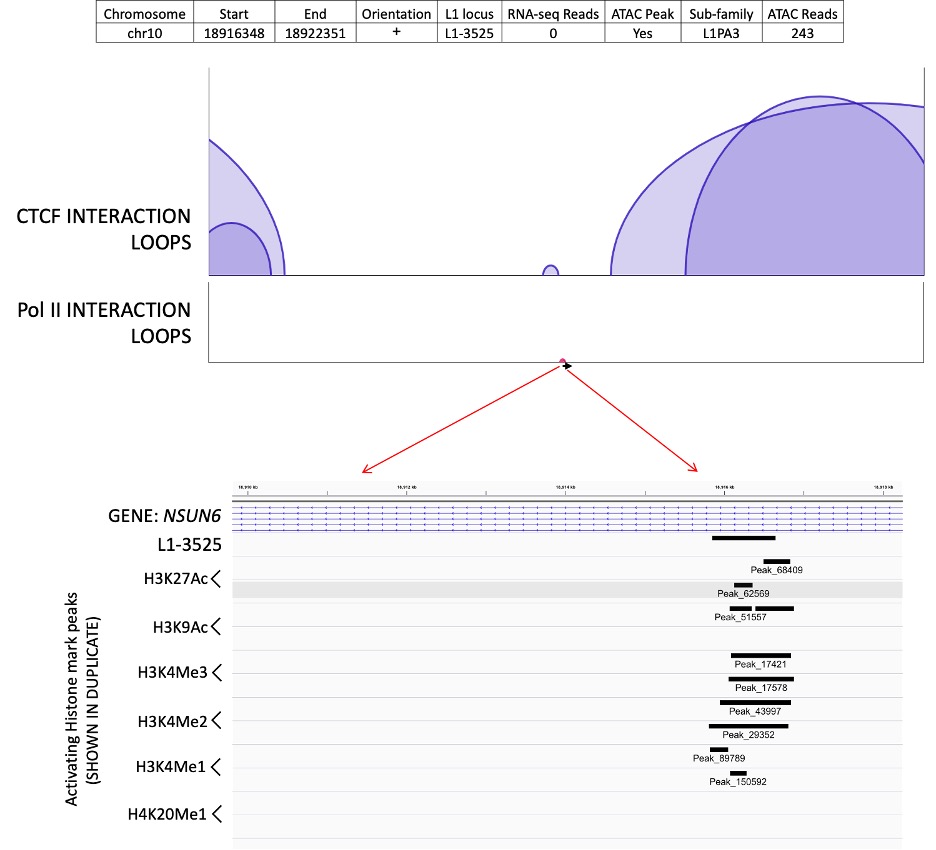


**Supplemental Figure 6Q**

**Supplemental Figure 6. Long-distance interactions of individual L1-loci in MCF7 cells. (A-AA)** Diagrams of L1-0728 **(A)**, L1-3682 **(B)**, L1-1867 **(C)**, L1-2476 **(D)**, L1-3165 **(E)**, L1-0029 **(F)**, L1-3455 **(G)**, and L1-1685 **(H)**, and L1-3239 **(I)** represent nine of the ten highest expressed loci in MCF7 cells. Diagrams of L1-2855 **(J)**, L1-0225 **(K)**, L1-0986 **(L)**, L1-4910 **(M)**, L1-5151 **(N)**, L1-1469 **(O)**, L1-0482 **(P)**, L1-3525 **(Q)**, and L1-4180 **(R)** represent nine of the ten transitional loci (unexpressed loci overlapping with an ATAC peak) with the largest peaks produced by mapping of ATAC sequencing reads. Diagrams of L1-0518 **(S)**, L1-1501 **(T)**, L1-1960 **(U)**, L1-4228 **(V)**, L1-4249 **(W)**, L1-4821 **(X)**, L1-4938 **(Y)**, L1-5330 **(Z)**, and L1-5742 **(AA)** represent nine of ten random unexpressed loci in MCF7 cells. Random loci were selected to ensure representation from each L1PA1-8 subfamily. One transitional L1Hs locus, L1-1337, was excluded from this analysis due to poor mappability. Each diagram includes information regarding the locus location (chromosome, start site, end site, and orientation), RNA-seq reads, presence of an ATAC peak, L1 sub-family, and number of reads from ATAC sequencing. Loops indicate CTCF binding sites (purple, shaded) within 500 kb of the L1 start site (black arrow). RNA polymerase II (Pol II) loops (red) are only shown if the pol II binding site overlaps within 500bp of the L1 start site. The CTCF loops are shown at a scale of 500kb upstream and downstream of the L1 start site. Red arrows indicate magnification of the indicated genomic region, which contains information regarding the presence of the activating histone marks from previously described CHIP-seq data (Figure 3A). Results of analysis of all histone marks are shown from two experiments for both the L1 promoter and putative enhancer ends of the RNA polymerase II loops.


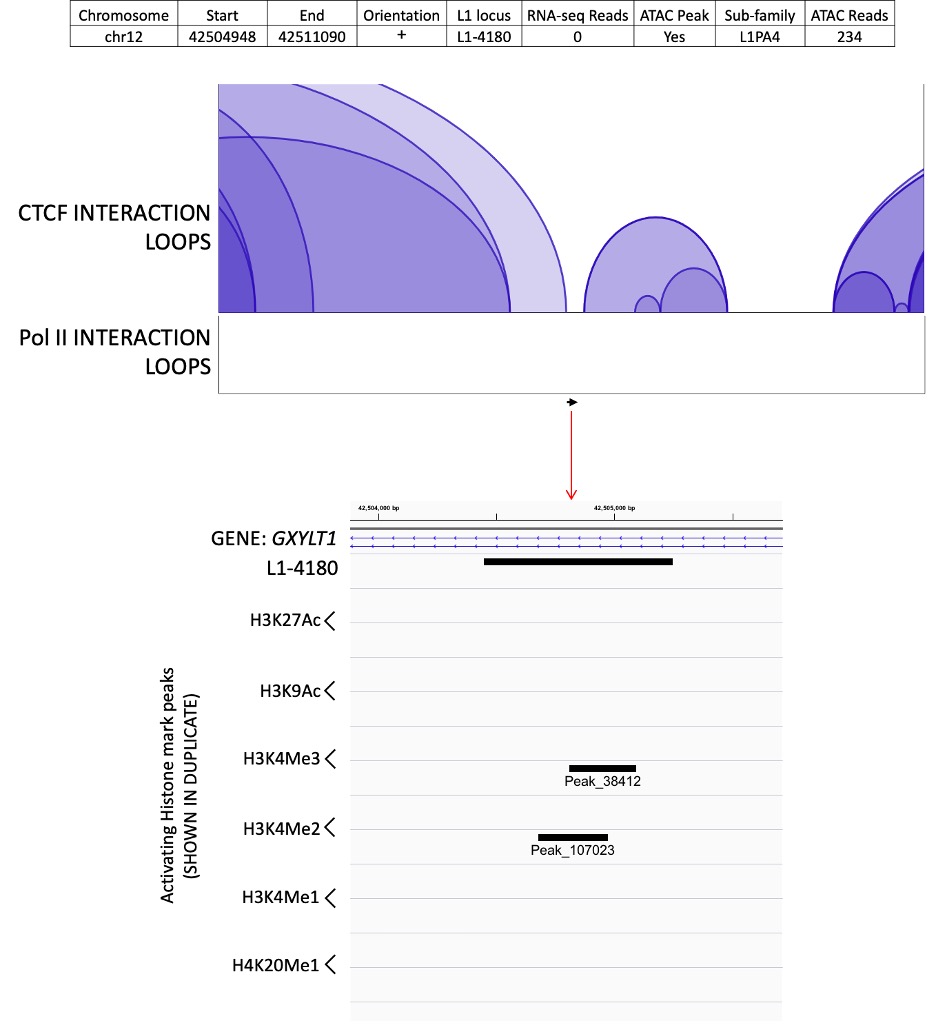


**Supplemental Figure 6R**

**Supplemental Figure 6. Long-distance interactions of individual L1-loci in MCF7 cells. (A-AA)** Diagrams of L1-0728 **(A)**, L1-3682 **(B)**, L1-1867 **(C)**, L1-2476 **(D)**, L1-3165 **(E)**, L1-0029 **(F)**, L1-3455 **(G)**, and L1-1685 **(H)**, and L1-3239 **(I)** represent nine of the ten highest expressed loci in MCF7 cells. Diagrams of L1-2855 **(J)**, L1-0225 **(K)**, L1-0986 **(L)**, L1-4910 **(M)**, L1-5151 **(N)**, L1-1469 **(O)**, L1-0482 **(P)**, L1-3525 **(Q)**, and L1-4180 **(R)** represent nine of the ten transitional loci (unexpressed loci overlapping with an ATAC peak) with the largest peaks produced by mapping of ATAC sequencing reads. Diagrams of L1-0518 **(S)**, L1-1501 **(T)**, L1-1960 **(U)**, L1-4228 **(V)**, L1-4249 **(W)**, L1-4821 **(X)**, L1-4938 **(Y)**, L1-5330 **(Z)**, and L1-5742 **(AA)** represent nine of ten random unexpressed loci in MCF7 cells. Random loci were selected to ensure representation from each L1PA1-8 subfamily. One transitional L1Hs locus, L1-1337, was excluded from this analysis due to poor mappability. Each diagram includes information regarding the locus location (chromosome, start site, end site, and orientation), RNA-seq reads, presence of an ATAC peak, L1 sub-family, and number of reads from ATAC sequencing. Loops indicate CTCF binding sites (purple, shaded) within 500 kb of the L1 start site (black arrow). RNA polymerase II (Pol II) loops (red) are only shown if the pol II binding site overlaps within 500bp of the L1 start site. The CTCF loops are shown at a scale of 500kb upstream and downstream of the L1 start site. Red arrows indicate magnification of the indicated genomic region, which contains information regarding the presence of the activating histone marks from previously described CHIP-seq data (Figure 3A). Results of analysis of all histone marks are shown from two experiments for both the L1 promoter and putative enhancer ends of the RNA polymerase II loops.


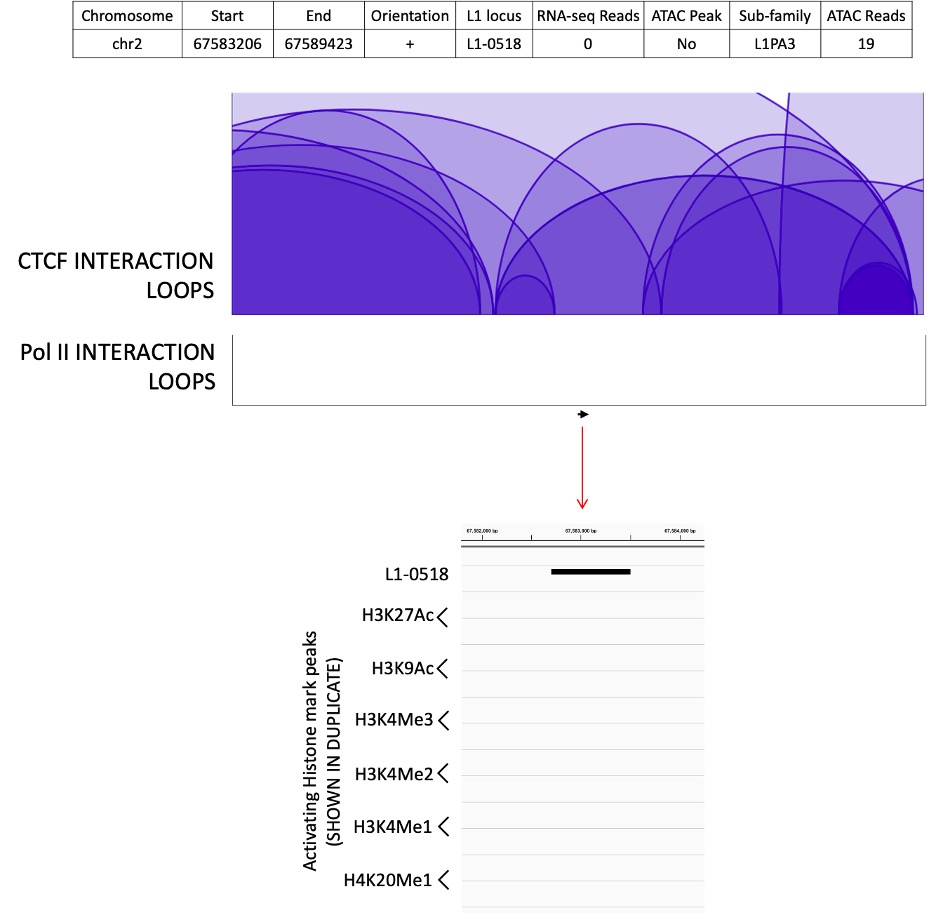


**Supplemental Figure 6S**

**Supplemental Figure 6. Long-distance interactions of individual L1-loci in MCF7 cells. (A-AA)** Diagrams of L1-0728 **(A)**, L1-3682 **(B)**, L1-1867 **(C)**, L1-2476 **(D)**, L1-3165 **(E)**, L1-0029 **(F)**, L1-3455 **(G)**, and L1-1685 **(H)**, and L1-3239 **(I)** represent nine of the ten highest expressed loci in MCF7 cells. Diagrams of L1-2855 **(J)**, L1-0225 **(K)**, L1-0986 **(L)**, L1-4910 **(M)**, L1-5151 **(N)**, L1-1469 **(O)**, L1-0482 **(P)**, L1-3525 **(Q)**, and L1-4180 **(R)** represent nine of the ten transitional loci (unexpressed loci overlapping with an ATAC peak) with the largest peaks produced by mapping of ATAC sequencing reads. Diagrams of L1-0518 **(S)**, L1-1501 **(T)**, L1-1960 **(U)**, L1-4228 **(V)**, L1-4249 **(W)**, L1-4821 **(X)**, L1-4938 **(Y)**, L1-5330 **(Z)**, and L1-5742 **(AA)** represent nine of ten random unexpressed loci in MCF7 cells. Random loci were selected to ensure representation from each L1PA1-8 subfamily. One transitional L1Hs locus, L1-1337, was excluded from this analysis due to poor mappability. Each diagram includes information regarding the locus location (chromosome, start site, end site, and orientation), RNA-seq reads, presence of an ATAC peak, L1 sub-family, and number of reads from ATAC sequencing. Loops indicate CTCF binding sites (purple, shaded) within 500 kb of the L1 start site (black arrow). RNA polymerase II (Pol II) loops (red) are only shown if the pol II binding site overlaps within 500bp of the L1 start site. The CTCF loops are shown at a scale of 500kb upstream and downstream of the L1 start site. Red arrows indicate magnification of the indicated genomic region, which contains information regarding the presence of the activating histone marks from previously described CHIP-seq data (Figure 3A). Results of analysis of all histone marks are shown from two experiments for both the L1 promoter and putative enhancer ends of the RNA polymerase II loops.


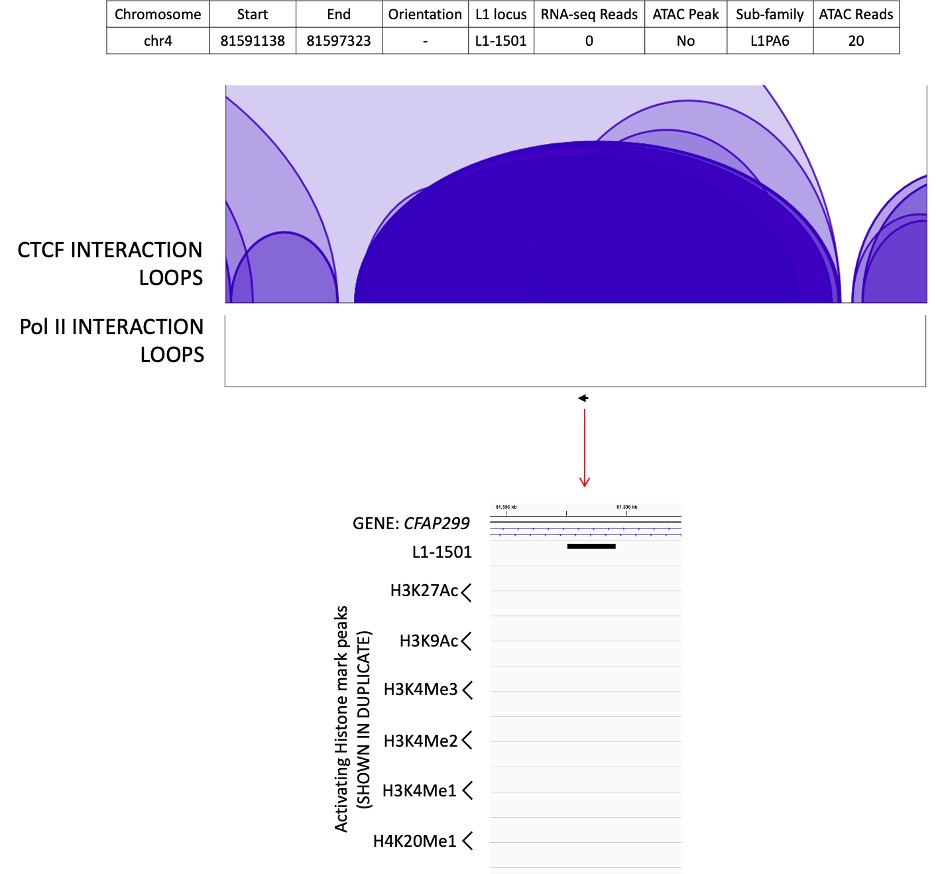


**Supplemental Figure 6T**

**Supplemental Figure 6. Long-distance interactions of individual L1-loci in MCF7 cells. (A-AA)** Diagrams of L1-0728 **(A)**, L1-3682 **(B)**, L1-1867 **(C)**, L1-2476 **(D)**, L1-3165 **(E)**, L1-0029 **(F)**, L1-3455 **(G)**, and L1-1685 **(H)**, and L1-3239 **(I)** represent nine of the ten highest expressed loci in MCF7 cells. Diagrams of L1-2855 **(J)**, L1-0225 **(K)**, L1-0986 **(L)**, L1-4910 **(M)**, L1-5151 **(N)**, L1-1469 **(O)**, L1-0482 **(P)**, L1-3525 **(Q)**, and L1-4180 **(R)** represent nine of the ten transitional loci (unexpressed loci overlapping with an ATAC peak) with the largest peaks produced by mapping of ATAC sequencing reads. Diagrams of L1-0518 **(S)**, L1-1501 **(T)**, L1-1960 **(U)**, L1-4228 **(V)**, L1-4249 **(W)**, L1-4821 **(X)**, L1-4938 **(Y)**, L1-5330 **(Z)**, and L1-5742 **(AA)** represent nine of ten random unexpressed loci in MCF7 cells. Random loci were selected to ensure representation from each L1PA1-8 subfamily. One transitional L1Hs locus, L1-1337, was excluded from this analysis due to poor mappability. Each diagram includes information regarding the locus location (chromosome, start site, end site, and orientation), RNA-seq reads, presence of an ATAC peak, L1 sub-family, and number of reads from ATAC sequencing. Loops indicate CTCF binding sites (purple, shaded) within 500 kb of the L1 start site (black arrow). RNA polymerase II (Pol II) loops (red) are only shown if the pol II binding site overlaps within 500bp of the L1 start site. The CTCF loops are shown at a scale of 500kb upstream and downstream of the L1 start site. Red arrows indicate magnification of the indicated genomic region, which contains information regarding the presence of the activating histone marks from previously described CHIP-seq data (Figure 3A). Results of analysis of all histone marks are shown from two experiments for both the L1 promoter and putative enhancer ends of the RNA polymerase II loops.


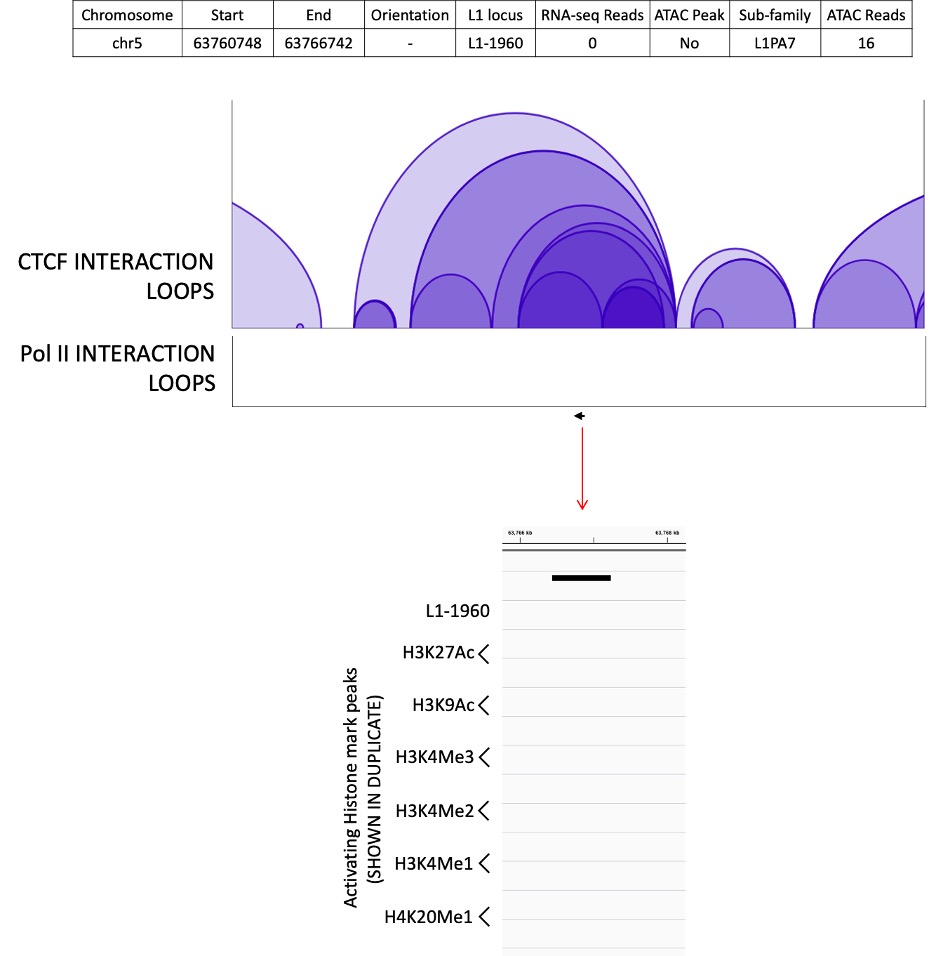


**Supplemental Figure 6U**

**Supplemental Figure 6. Long-distance interactions of individual L1-loci in MCF7 cells. (A-AA)** Diagrams of L1-0728 **(A)**, L1-3682 **(B)**, L1-1867 **(C)**, L1-2476 **(D)**, L1-3165 **(E)**, L1-0029 **(F)**, L1-3455 **(G)**, and L1-1685 **(H)**, and L1-3239 **(I)** represent nine of the ten highest expressed loci in MCF7 cells. Diagrams of L1-2855 **(J)**, L1-0225 **(K)**, L1-0986 **(L)**, L1-4910 **(M)**, L1-5151 **(N)**, L1-1469 **(O)**, L1-0482 **(P)**, L1-3525 **(Q)**, and L1-4180 **(R)** represent nine of the ten transitional loci (unexpressed loci overlapping with an ATAC peak) with the largest peaks produced by mapping of ATAC sequencing reads. Diagrams of L1-0518 **(S)**, L1-1501 **(T)**, L1-1960 **(U)**, L1-4228 **(V)**, L1-4249 **(W)**, L1-4821 **(X)**, L1-4938 **(Y)**, L1-5330 **(Z)**, and L1-5742 **(AA)** represent nine of ten random unexpressed loci in MCF7 cells. Random loci were selected to ensure representation from each L1PA1-8 subfamily. One transitional L1Hs locus, L1-1337, was excluded from this analysis due to poor mappability. Each diagram includes information regarding the locus location (chromosome, start site, end site, and orientation), RNA-seq reads, presence of an ATAC peak, L1 sub-family, and number of reads from ATAC sequencing. Loops indicate CTCF binding sites (purple, shaded) within 500 kb of the L1 start site (black arrow). RNA polymerase II (Pol II) loops (red) are only shown if the pol II binding site overlaps within 500bp of the L1 start site. The CTCF loops are shown at a scale of 500kb upstream and downstream of the L1 start site. Red arrows indicate magnification of the indicated genomic region, which contains information regarding the presence of the activating histone marks from previously described CHIP-seq data (Figure 3A). Results of analysis of all histone marks are shown from two experiments for both the L1 promoter and putative enhancer ends of the RNA polymerase II loops.


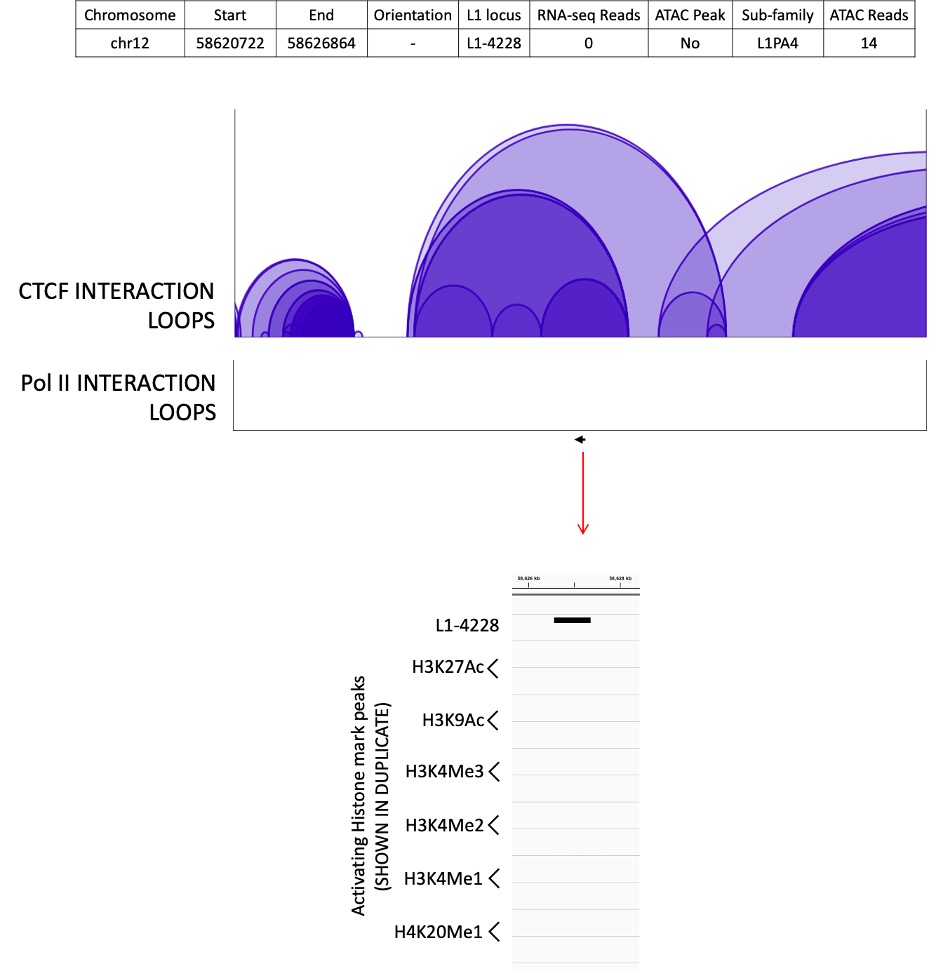


**Supplemental Figure 6V**

**Supplemental Figure 6. Long-distance interactions of individual L1-loci in MCF7 cells. (A-AA)** Diagrams of L1-0728 **(A)**, L1-3682 **(B)**, L1-1867 **(C)**, L1-2476 **(D)**, L1-3165 **(E)**, L1-0029 **(F)**, L1-3455 **(G)**, and L1-1685 **(H)**, and L1-3239 **(I)** represent nine of the ten highest expressed loci in MCF7 cells. Diagrams of L1-2855 **(J)**, L1-0225 **(K)**, L1-0986 **(L)**, L1-4910 **(M)**, L1-5151 **(N)**, L1-1469 **(O)**, L1-0482 **(P)**, L1-3525 **(Q)**, and L1-4180 **(R)** represent nine of the ten transitional loci (unexpressed loci overlapping with an ATAC peak) with the largest peaks produced by mapping of ATAC sequencing reads. Diagrams of L1-0518 **(S)**, L1-1501 **(T)**, L1-1960 **(U)**, L1-4228 **(V)**, L1-4249 **(W)**, L1-4821 **(X)**, L1-4938 **(Y)**, L1-5330 **(Z)**, and L1-5742 **(AA)** represent nine of ten random unexpressed loci in MCF7 cells. Random loci were selected to ensure representation from each L1PA1-8 subfamily. One transitional L1Hs locus, L1-1337, was excluded from this analysis due to poor mappability. Each diagram includes information regarding the locus location (chromosome, start site, end site, and orientation), RNA-seq reads, presence of an ATAC peak, L1 sub-family, and number of reads from ATAC sequencing. Loops indicate CTCF binding sites (purple, shaded) within 500 kb of the L1 start site (black arrow). RNA polymerase II (Pol II) loops (red) are only shown if the pol II binding site overlaps within 500bp of the L1 start site. The CTCF loops are shown at a scale of 500kb upstream and downstream of the L1 start site. Red arrows indicate magnification of the indicated genomic region, which contains information regarding the presence of the activating histone marks from previously described CHIP-seq data (Figure 3A). Results of analysis of all histone marks are shown from two experiments for both the L1 promoter and putative enhancer ends of the RNA polymerase II loops.


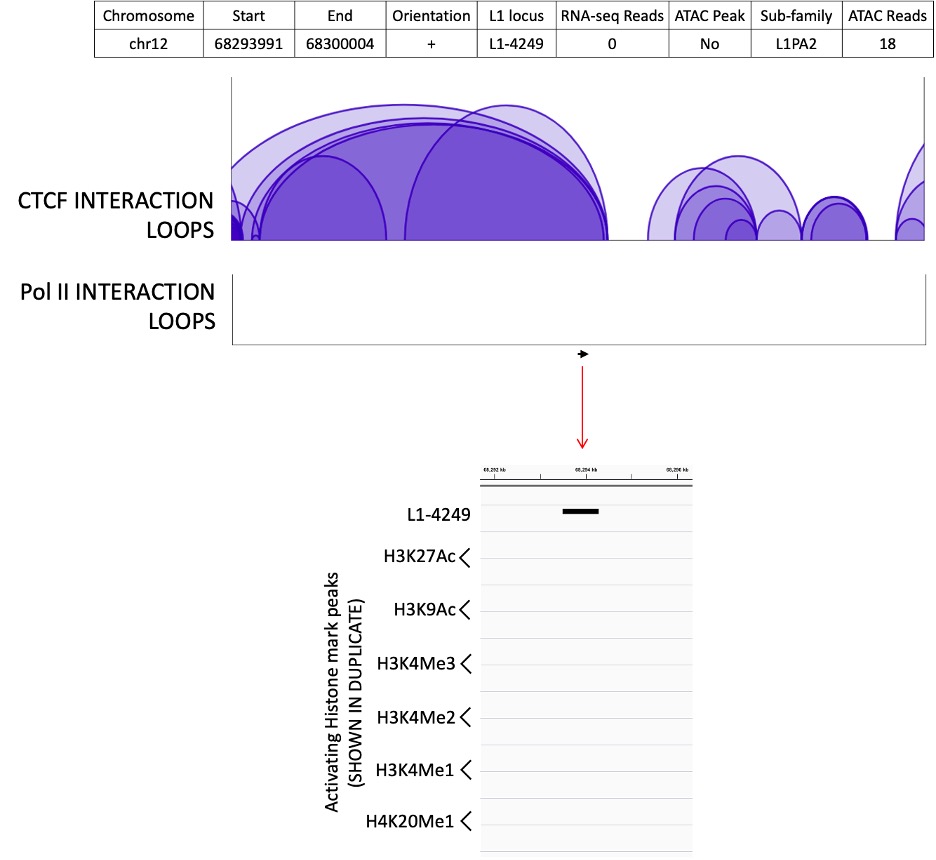


**Supplemental Figure 6W**

**Supplemental Figure 6. Long-distance interactions of individual L1-loci in MCF7 cells. (A-AA)** Diagrams of L1-0728 **(A)**, L1-3682 **(B)**, L1-1867 **(C)**, L1-2476 **(D)**, L1-3165 **(E)**, L1-0029 **(F)**, L1-3455 **(G)**, and L1-1685 **(H)**, and L1-3239 **(I)** represent nine of the ten highest expressed loci in MCF7 cells. Diagrams of L1-2855 **(J)**, L1-0225 **(K)**, L1-0986 **(L)**, L1-4910 **(M)**, L1-5151 **(N)**, L1-1469 **(O)**, L1-0482 **(P)**, L1-3525 **(Q)**, and L1-4180 **(R)** represent nine of the ten transitional loci (unexpressed loci overlapping with an ATAC peak) with the largest peaks produced by mapping of ATAC sequencing reads. Diagrams of L1-0518 **(S)**, L1-1501 **(T)**, L1-1960 **(U)**, L1-4228 **(V)**, L1-4249 **(W)**, L1-4821 **(X)**, L1-4938 **(Y)**, L1-5330 **(Z)**, and L1-5742 **(AA)** represent nine of ten random unexpressed loci in MCF7 cells. Random loci were selected to ensure representation from each L1PA1-8 subfamily. One transitional L1Hs locus, L1-1337, was excluded from this analysis due to poor mappability. Each diagram includes information regarding the locus location (chromosome, start site, end site, and orientation), RNA-seq reads, presence of an ATAC peak, L1 sub-family, and number of reads from ATAC sequencing. Loops indicate CTCF binding sites (purple, shaded) within 500 kb of the L1 start site (black arrow). RNA polymerase II (Pol II) loops (red) are only shown if the pol II binding site overlaps within 500bp of the L1 start site. The CTCF loops are shown at a scale of 500kb upstream and downstream of the L1 start site. Red arrows indicate magnification of the indicated genomic region, which contains information regarding the presence of the activating histone marks from previously described CHIP-seq data (Figure 3A). Results of analysis of all histone marks are shown from two experiments for both the L1 promoter and putative enhancer ends of the RNA polymerase II loops.


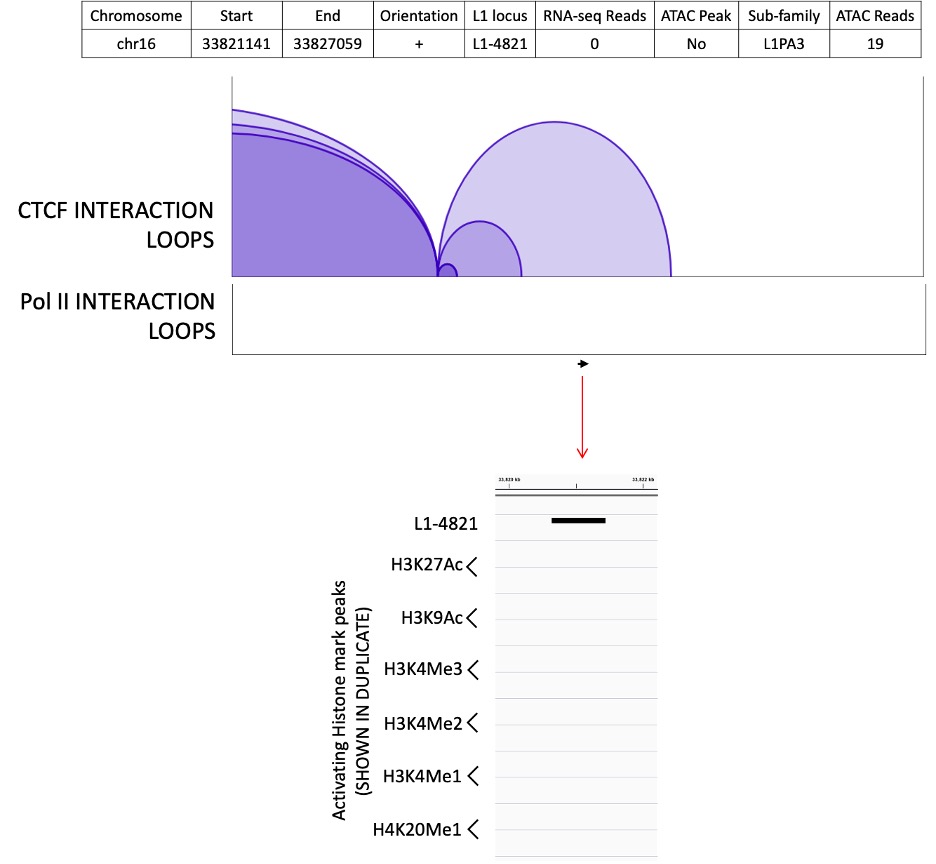


**Supplemental Figure 6X**

**Supplemental Figure 6. Long-distance interactions of individual L1-loci in MCF7 cells. (A-AA)** Diagrams of L1-0728 **(A)**, L1-3682 **(B)**, L1-1867 **(C)**, L1-2476 **(D)**, L1-3165 **(E)**, L1-0029 **(F)**, L1-3455 **(G)**, and L1-1685 **(H)**, and L1-3239 **(I)** represent nine of the ten highest expressed loci in MCF7 cells. Diagrams of L1-2855 **(J)**, L1-0225 **(K)**, L1-0986 **(L)**, L1-4910 **(M)**, L1-5151 **(N)**, L1-1469 **(O)**, L1-0482 **(P)**, L1-3525 **(Q)**, and L1-4180 **(R)** represent nine of the ten transitional loci (unexpressed loci overlapping with an ATAC peak) with the largest peaks produced by mapping of ATAC sequencing reads. Diagrams of L1-0518 **(S)**, L1-1501 **(T)**, L1-1960 **(U)**, L1-4228 **(V)**, L1-4249 **(W)**, L1-4821 **(X)**, L1-4938 **(Y)**, L1-5330 **(Z)**, and L1-5742 **(AA)** represent nine of ten random unexpressed loci in MCF7 cells. Random loci were selected to ensure representation from each L1PA1-8 subfamily. One transitional L1Hs locus, L1-1337, was excluded from this analysis due to poor mappability. Each diagram includes information regarding the locus location (chromosome, start site, end site, and orientation), RNA-seq reads, presence of an ATAC peak, L1 sub-family, and number of reads from ATAC sequencing. Loops indicate CTCF binding sites (purple, shaded) within 500 kb of the L1 start site (black arrow). RNA polymerase II (Pol II) loops (red) are only shown if the pol II binding site overlaps within 500bp of the L1 start site. The CTCF loops are shown at a scale of 500kb upstream and downstream of the L1 start site. Red arrows indicate magnification of the indicated genomic region, which contains information regarding the presence of the activating histone marks from previously described CHIP-seq data (Figure 3A). Results of analysis of all histone marks are shown from two experiments for both the L1 promoter and putative enhancer ends of the RNA polymerase II loops.


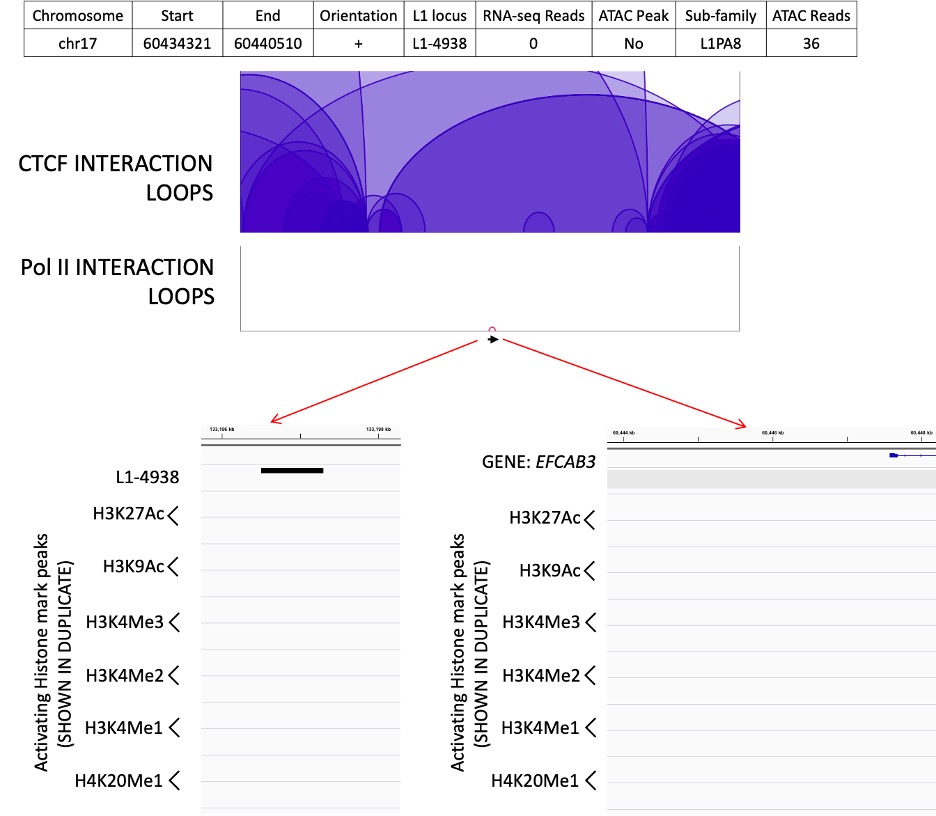


**Supplemental Figure 6Y**

**Supplemental Figure 6. Long-distance interactions of individual L1-loci in MCF7 cells. (A-AA)** Diagrams of L1-0728 **(A)**, L1-3682 **(B)**, L1-1867 **(C)**, L1-2476 **(D)**, L1-3165 **(E)**, L1-0029 **(F)**, L1-3455 **(G)**, and L1-1685 **(H)**, and L1-3239 **(I)** represent nine of the ten highest expressed loci in MCF7 cells. Diagrams of L1-2855 **(J)**, L1-0225 **(K)**, L1-0986 **(L)**, L1-4910 **(M)**, L1-5151 **(N)**, L1-1469 **(O)**, L1-0482 **(P)**, L1-3525 **(Q)**, and L1-4180 **(R)** represent nine of the ten transitional loci (unexpressed loci overlapping with an ATAC peak) with the largest peaks produced by mapping of ATAC sequencing reads. Diagrams of L1-0518 **(S)**, L1-1501 **(T)**, L1-1960 **(U)**, L1-4228 **(V)**, L1-4249 **(W)**, L1-4821 **(X)**, L1-4938 **(Y)**, L1-5330 **(Z)**, and L1-5742 **(AA)** represent nine of ten random unexpressed loci in MCF7 cells. Random loci were selected to ensure representation from each L1PA1-8 subfamily. One transitional L1Hs locus, L1-1337, was excluded from this analysis due to poor mappability. Each diagram includes information regarding the locus location (chromosome, start site, end site, and orientation), RNA-seq reads, presence of an ATAC peak, L1 sub-family, and number of reads from ATAC sequencing. Loops indicate CTCF binding sites (purple, shaded) within 500 kb of the L1 start site (black arrow). RNA polymerase II (Pol II) loops (red) are only shown if the pol II binding site overlaps within 500bp of the L1 start site. The CTCF loops are shown at a scale of 500kb upstream and downstream of the L1 start site. Red arrows indicate magnification of the indicated genomic region, which contains information regarding the presence of the activating histone marks from previously described CHIP-seq data (Figure 3A). Results of analysis of all histone marks are shown from two experiments for both the L1 promoter and putative enhancer ends of the RNA polymerase II loops.


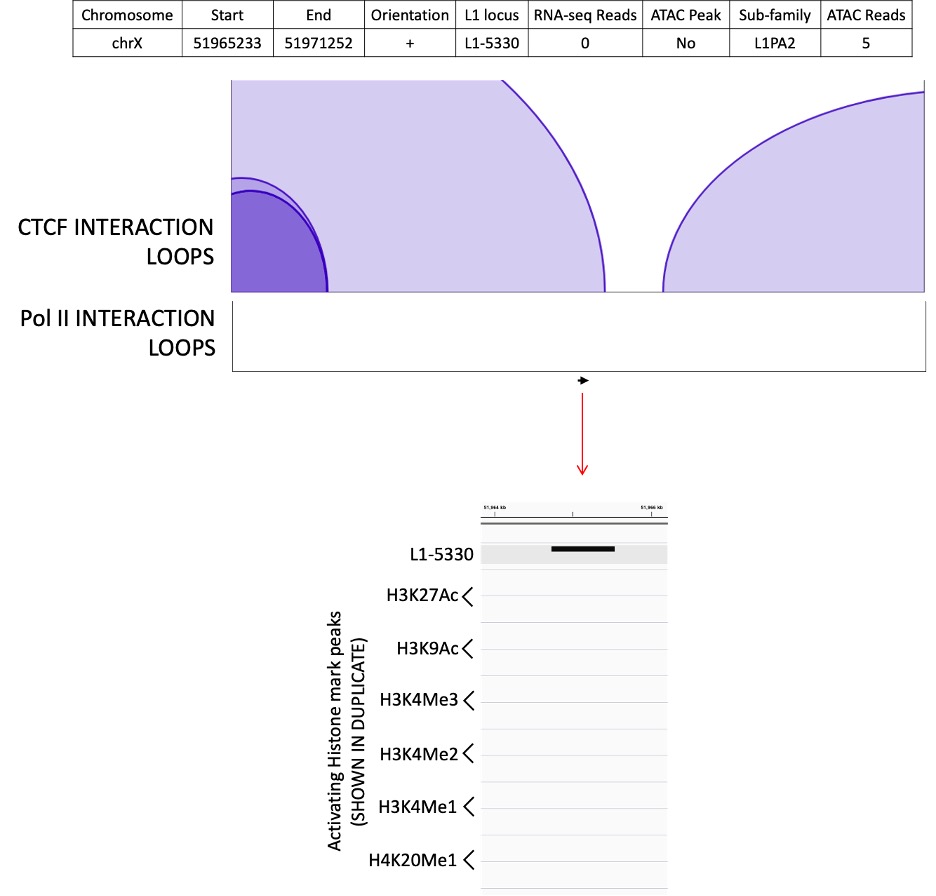


**Supplemental Figure 6Z**

**Supplemental Figure 6. Long-distance interactions of individual L1-loci in MCF7 cells. (A-AA)** Diagrams of L1-0728 **(A)**, L1-3682 **(B)**, L1-1867 **(C)**, L1-2476 **(D)**, L1-3165 **(E)**, L1-0029 **(F)**, L1-3455 **(G)**, and L1-1685 **(H)**, and L1-3239 **(I)** represent nine of the ten highest expressed loci in MCF7 cells. Diagrams of L1-2855 **(J)**, L1-0225 **(K)**, L1-0986 **(L)**, L1-4910 **(M)**, L1-5151 **(N)**, L1-1469 **(O)**, L1-0482 **(P)**, L1-3525 **(Q)**, and L1-4180 **(R)** represent nine of the ten transitional loci (unexpressed loci overlapping with an ATAC peak) with the largest peaks produced by mapping of ATAC sequencing reads. Diagrams of L1-0518 **(S)**, L1-1501 **(T)**, L1-1960 **(U)**, L1-4228 **(V)**, L1-4249 **(W)**, L1-4821 **(X)**, L1-4938 **(Y)**, L1-5330 **(Z)**, and L1-5742 **(AA)** represent nine of ten random unexpressed loci in MCF7 cells. Random loci were selected to ensure representation from each L1PA1-8 subfamily. One transitional L1Hs locus, L1-1337, was excluded from this analysis due to poor mappability. Each diagram includes information regarding the locus location (chromosome, start site, end site, and orientation), RNA-seq reads, presence of an ATAC peak, L1 sub-family, and number of reads from ATAC sequencing. Loops indicate CTCF binding sites (purple, shaded) within 500 kb of the L1 start site (black arrow). RNA polymerase II (Pol II) loops (red) are only shown if the pol II binding site overlaps within 500bp of the L1 start site. The CTCF loops are shown at a scale of 500kb upstream and downstream of the L1 start site. Red arrows indicate magnification of the indicated genomic region, which contains information regarding the presence of the activating histone marks from previously described CHIP-seq data (Figure 3A). Results of analysis of all histone marks are shown from two experiments for both the L1 promoter and putative enhancer ends of the RNA polymerase II loops.


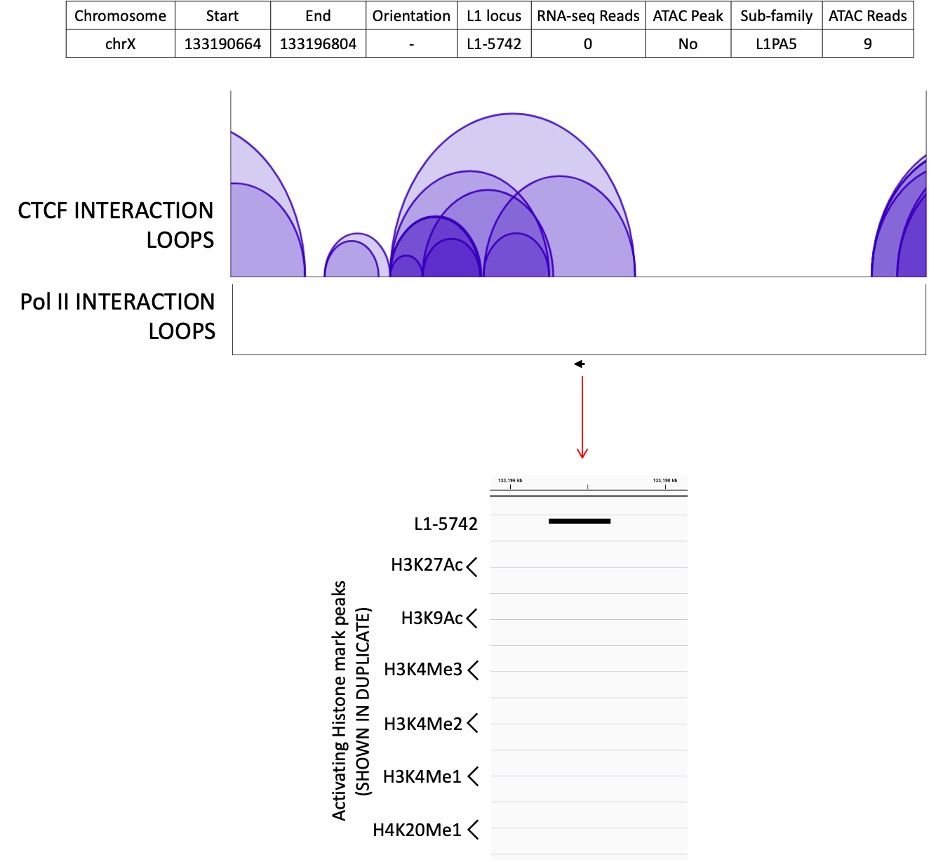


**Supplemental Figure 6AA**

**Supplemental Figure 6. Long-distance interactions of individual L1-loci in MCF7 cells. (A-AA)** Diagrams of L1-0728 **(A)**, L1-3682 **(B)**, L1-1867 **(C)**, L1-2476 **(D)**, L1-3165 **(E)**, L1-0029 **(F)**, L1-3455 **(G)**, and L1-1685 **(H)**, and L1-3239 **(I)** represent nine of the ten highest expressed loci in MCF7 cells. Diagrams of L1-2855 **(J)**, L1-0225 **(K)**, L1-0986 **(L)**, L1-4910 **(M)**, L1-5151 **(N)**, L1-1469 **(O)**, L1-0482 **(P)**, L1-3525 **(Q)**, and L1-4180 **(R)** represent nine of the ten transitional loci (unexpressed loci overlapping with an ATAC peak) with the largest peaks produced by mapping of ATAC sequencing reads. Diagrams of L1-0518 **(S)**, L1-1501 **(T)**, L1-1960 **(U)**, L1-4228 **(V)**, L1-4249 **(W)**, L1-4821 **(X)**, L1-4938 **(Y)**, L1-5330 **(Z)**, and L1-5742 **(AA)** represent nine of ten random unexpressed loci in MCF7 cells. Random loci were selected to ensure representation from each L1PA1-8 subfamily. One transitional L1Hs locus, L1-1337, was excluded from this analysis due to poor mappability. Each diagram includes information regarding the locus location (chromosome, start site, end site, and orientation), RNA-seq reads, presence of an ATAC peak, L1 sub-family, and number of reads from ATAC sequencing. Loops indicate CTCF binding sites (purple, shaded) within 500 kb of the L1 start site (black arrow). RNA polymerase II (Pol II) loops (red) are only shown if the pol II binding site overlaps within 500bp of the L1 start site. The CTCF loops are shown at a scale of 500kb upstream and downstream of the L1 start site. Red arrows indicate magnification of the indicated genomic region, which contains information regarding the presence of the activating histone marks from previously described CHIP-seq data (Figure 3A). Results of analysis of all histone marks are shown from two experiments for both the L1 promoter and putative enhancer ends of the RNA polymerase II loops.


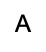

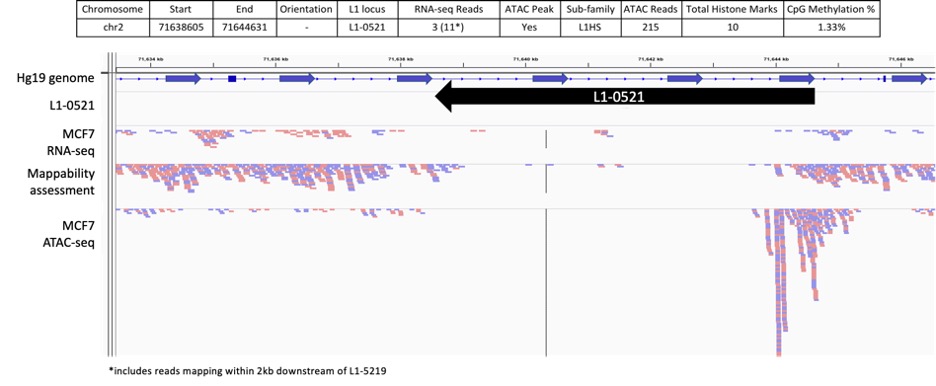


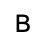


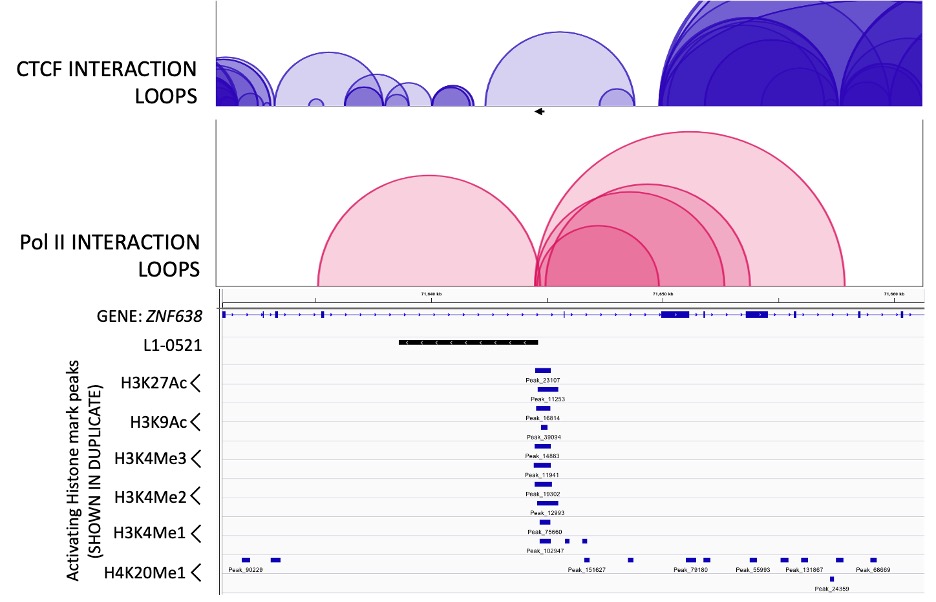


**Supplemental Figure 7. L1-0521 is a poorly mappable L1Hs element that is likely expressed in MCF7 cells. (A)** Manual assessment of L1-0521 locus status in IGV. L1-0521 genomic location, expression, sub-family, and epigenetic data are displayed above the image. Alignments of RNA-seq and ATAC-seq reads mapped to L1-0521 in MCF7 cells and mappability assessment are visualized in IGV. Note poor mappability of this locus, characteristic of many L1Hs elements. **(B)** Long-distance interactions of L1-0521. Loops indicate CTCF binding sites (purple, shaded) within 500kb of the L1 start site (black arrow). RNA polymerase II (Pol II) loops (red) are only shown if the pol II binding site is within 500bp of the L1 start site. Visualization of the pol II loop is shown at a scale of 15kb upstream and downstream of the L1 start site. The pol II loops align with the IGV screenshot displaying the genomic context of L1-0521 and presence of the activating histone marks from previously described CHIP-seq data (Figure 3A) is visualized. The presence of each histone mark is determined from two experiments.


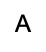

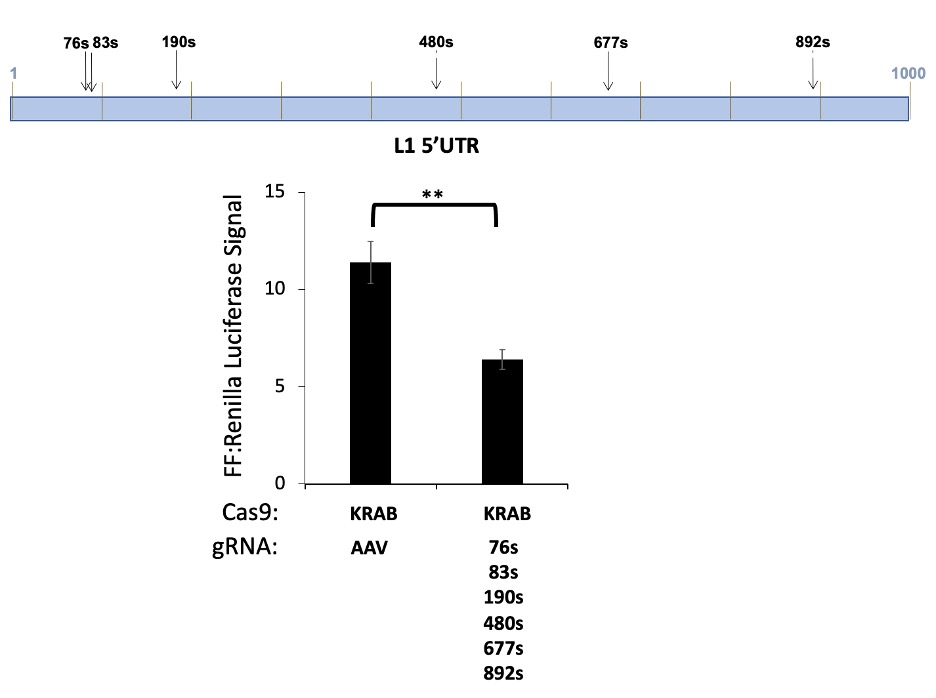


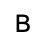


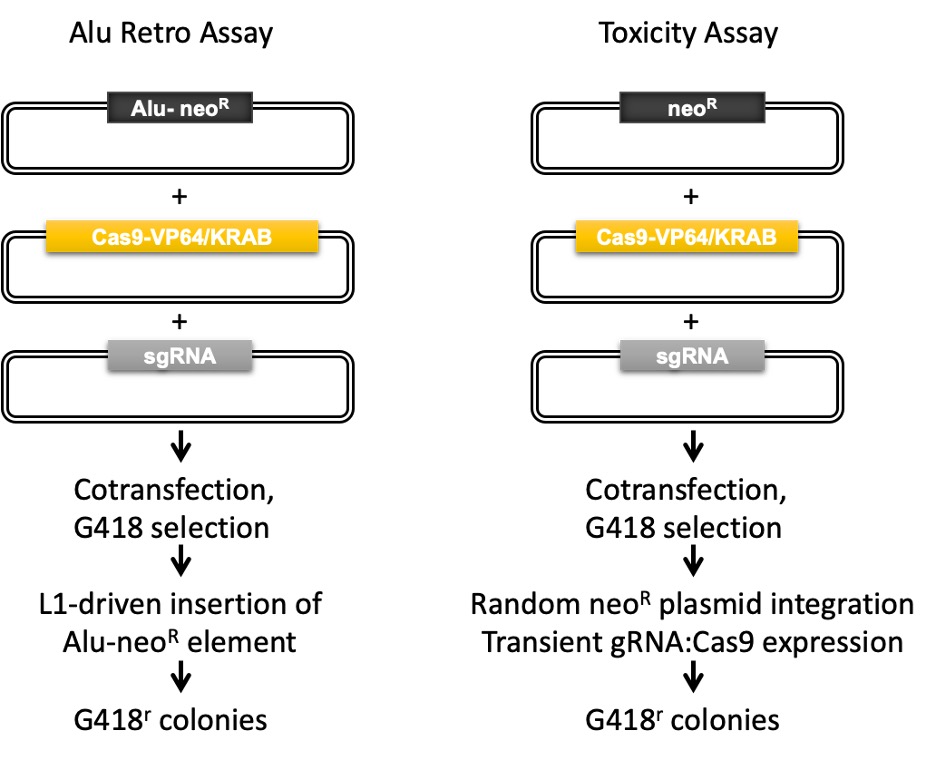


**Supplemental Figure 8. Manipulation of endogenous L1 expression by a CRISPR/Cas9i-based inactivating system. (A)** Top: L1 promoter schematic with vertical arrows indicating the locations of gRNAs used with the CRISPR/Cas9i system. Bottom: CAS9-KRAB fusion system and gRNAs targeting L1 5’UTR are used to test their effect on expression of Firefly (FF) luciferase driven by the L1 promoter in transient transfections Significance determined by student’s t-test (**, *p* < .01) (n = 3). **(B)** Schematic of Alu retrotransposition assay and toxicity assay. Plasmids expressing CAS9-VP64 (or CAS9-KRAB) and gRNAs are co-transfected with a plasmid expressing either Alu reporter plasmid (Alu retro assay) or a plasmid expressing neomycin resistance (toxicity assay). After two weeks of selection with G418 media, resistant colonies are stained and counted.


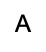

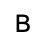

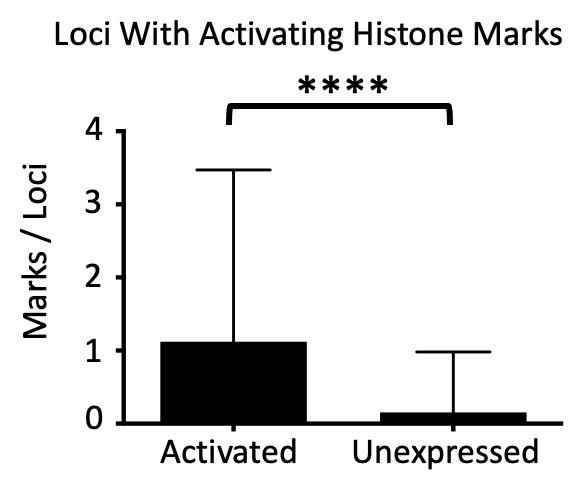

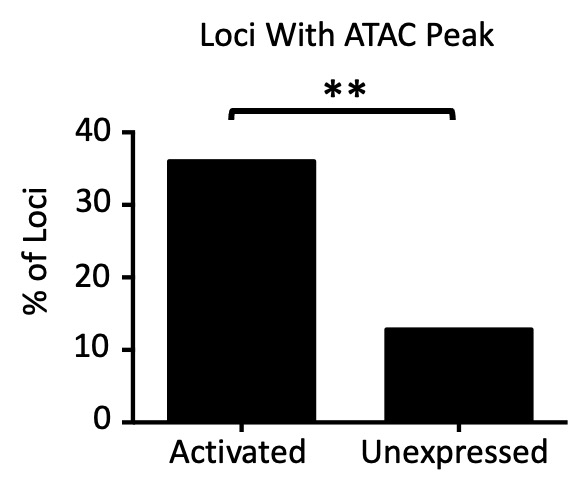


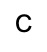


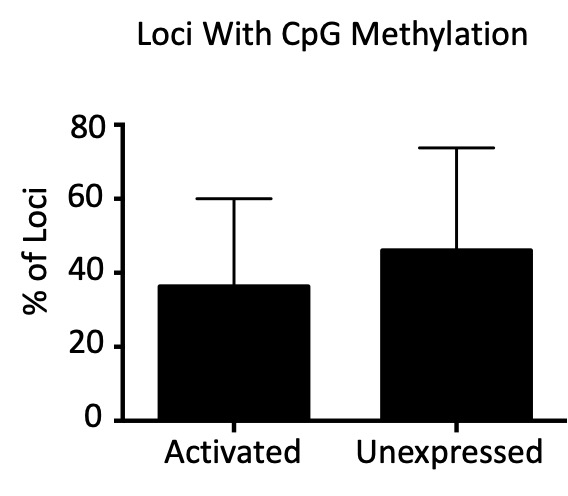


**Supplemental Figure 9. Epigenetic features of endogenous L1 loci activated by CRISPR/Cas9a system.** Epigenetic analysis of transactivated loci shown in Figure 10. We defined activated loci as loci with 0 mapped reads in the control sample and 5+ reads in the pool 2 sample of Figure 10 (n = 25). We defined unexpressed loci as having 0 mapped reads in both the pool 2 and control samples of Figure 10 (n = 3,638). **(A)** We compared activated and unexpressed loci in regard to their ATAC-peak status and found a significantly higher association of expressed L1 loci with an ATAC peak (36% vs. 12.81%; **, *p* = .0017)**.** Significance was determined by Chi Square analysis with Yates’ correction. **(B)** We compared activated and unexpressed loci in regard to the number of active histone marks at their promoters and found a significant difference in the average number of activating histone marks between the two groups (1.12 marks vs. .15 marks, *p* < .0001). Significance determined by student’s t-test. **(C)** We compared activated and unexpressed loci in regard to percent of methylated CpGs and found no significance between the two groups (36.28% vs. 46.01%; *p* = .087). Significance determined by student’s t-test. For the CpG methylation analysis, one activated locus and 146 unexpressed loci were unmapped following bisulfite sequencing and thus excluded from this analysis.
