# Supplementary material for: Analysis of epigenetic features characteristic of L1 loci expressed in human cells
Source: Nucleic Acids Res. 2022 Jan 31;50(4):1888–907. doi: 10.1093/nar/gkac013 (PMC8887483; doi:10.1093/nar/gkac013)
Supplement: gkac013_Supplemental_Files [file gkac013_supplemental_files.zip › SUPP_TABLE_1_-_Revised.docx]

Supplemental Table 1. Genomic information on the full length L1 loci present in the TTC28 locus and their epigenetic features determined in MCF7, HeLa, and HEK293 cells.
